# Supplementary material for: Investigating the impact of physical activity on mitochondrial function in Parkinson’s disease (PARKEX): Study protocol for A randomized controlled clinical trial
Source: PLoS One. 2023 Nov 22;18(11):e0293774. doi: 10.1371/journal.pone.0293774 (PMC10664890; doi:10.1371/journal.pone.0293774)
Supplement: S1 File — (DOCX) [file pone.0293774.s003.docx]

**Faculty of Psychology, Education and Sport Sciences Blanquerna.**

**Ramon Llull University**

**Department of Sport and Exercise Sciences**

**Doctoral Program in Education Sciences and Sports (Academic Year 2020-2021)**

**Title**

**“Assessment of mitochondrial function, using skin fibroblasts as a biomarker, in patients with Parkinson's disease: effects of two Physical Activity programs on motor function, quality of life, sleep, cognitive aspects and mood.”**

*The nature of this thesis arises from the main pillars of international cooperation in research networks and has its origin in the international collaboration between the University of Blanquerna (Barcelona-Spain) and the Center for Neuroscience and Cell Biology at the University of Coimbra (CNC-UC), Portugal. He will undoubtedly contribute extensively to uniting the important research areas of Mitochondrial Health Benefits of Physical Exercise and Parkinson Disease. These research areas are fundamental to human health and uniting them will be very important. There is a great need for knowledge on the role of mitochondria in disease developmental and how mitochondrial activity can be modulated in Parkinson by exercise. I feel I can say this with certainty as I was one of authors uncovering the mitochondrial bioenergetic defects in human skin fibroblasts from sporadic Parkinson’s disease patients.*

**Co-supervisor of the PhD Thesis:**

PhD Susana P. Pereira CNC - Center for Neuroscience and Cell Biology, CIBB - Centre for Innovative Biomedicine and Biotechnology, University of Coimbra, IIIUC - Institute for Interdisciplinary Research, University of

Coimbra, Coimbra, Portugal ITR – Laboratory for Integrative and Translational Research in Population Health, LaMetEx - Laboratory of Metabolism and Exercise, CIAFEL - Research Center in Physical Activity, health and Leisure, Faculty of

Sports, University of Porto, Portugal

*Biomedicine is a field dedicated to the advancement of human medicine. The role of research in Biomedicine in this project has a very relevant role, since different disciplines are mixed such as Physiology, Medicine, Biochemistry, Physical Activity Sciences, Neuropsychology, among others. In this doctoral thesis, Biomedicine will be used as a tool to determine if two different physical activity programs improve Mitochondrial function, motor function, quality of life, sleep, cognitive aspects and mood in patients with Parkinson's disease. This great step is possible thanks to the international collaboration between the Center for Neuroscience and Cell Biology, of the University of Coimbra, and the SAFE research group, of the Universitat Blanquerna-URL (FPCEE).*

*Two leading and multidisciplinary groups that have come together to address the assessment of mitochondrial function using skin fibroblasts as a biomarker. These results will contribute to the field of Biomedicine, and will undoubtedly help to improve the quality of life and diagnosis of patients with Parkinson's.*

**Co-supervisor of the PhD Tesis:**

PhD Joel Montané Facultat Ciències de la Salut Universitat Blanquerna-URL

Barcelona

##### PhD candidate: Juan Carlos Magaña Gallardo

**December 5th 2022**

Table of contents

[1. Abstract 3](#_Toc149815767)

[2. Justification 4](#_Toc149815768)

[3. The topic and the objectives 7](#_Toc149815769)

[4. Theoretical Background and Current State of the Topic 8](#_Toc149815770)

[5. Hypothesis and Research Question 9](#_Toc149815771)

[6. Design 10](#_Toc149815772)

[7. Ethics 18](#_Toc149815773)

[8. Applicability, Limitations, and Future Directions 19](#_Toc149815774)

[9. Chronogram 20](#_Toc149815775)

[10. Resources 21](#_Toc149815776)

[11. Investigators 23](#_Toc149815777)

[12. Difusion plan, communication, oral presentations and prizes, 24](#_Toc149815778)

[13. References 26](#_Toc149815779)

[14. Annexes 28](#_Toc149815780)

# Abstract

**Introduction:** Parkinson's disease (PD) is a disorder characterized by the progressive degeneration of dopaminergic neurons resulting in dopamine deficiency in the striatum. Mitochondrial dysfunction and oxidative stress are associated with PD and are intrinsic factors related to its pathogenesis. Physical activity (PA) increases cognitive ability in older adults, attenuating motor deficits, increasing new neuron formation, ameliorating neurological impairments, and impeding age-related neuronal loss. In addition, skin fibroblasts have been identified as surrogate indicators of pathogenic processes correlating with clinical measures.

Thus, the present study aims to compare the effects of two different PA programs in PD patients analyzing relevant clinical aspects and the impact on mitochondrial function in patients' skin fibroblasts, used here as biomarker for metabolism improvement and disease progression.

**Methods:** Patients with clinical diagnosis of PD in the stages from 1 (mild) to 3 (moderate) according to Hoehn and Yahr Scale (n=24) will be recruited and randomized into three matched groups. The effects of two different PA programs will be compared. The first group (n=8) will perform basic physical training (BPT) based on strength and resistance; a second group will perform BPT combined with functional exercises (BPTFE), including exercises aimed to stimulate the specific sensorimotor pathways that are most affected in PD (proprioception-balance-coordination) together with cognitive and motor training; a third group will serve as control (sedentary group; Sed). Subjects will perform 3 sessions per week for 16 weeks. Motor function, quality of life, sleep quality, cognitive aspects and humor will be evaluated before and after intervention. A metabolic characterization of skin fibroblast will be performed by respirometry using the Seahorse XFe96, and by measuring ATP levels and mitochondria-related transcripts and proteins to determine the bioenergetic cellular deficits and characterize the metabolic remodeling induced by the exercise programs.

**Results:** We predict that the application of BPT and BPTFE programs will ameliorate relevant clinical aspects of the disease by improving systemic mitochondrial function, restoring mitochondrial metabolism, gene expression patterns, and ultimately, translating into mitochondrial neuroprotective effects.

**Conclusions:** The comparison of BPT and BPTFE PA programs will provide insights into the degree of amelioration in several relevant aspects of PD, including motor function, quality of life, quality of sleep, cognitive aspects, humour, and mitochondrial function. Such changes can have a positive clinical impact and PD skin fibroblasts may be used as a biomarker for PD diagnosis and disease progression assessment.

# Justification

According to the WHO [1] neurological disorders (ND) are present in pathologies and diseases of millions of people around the world. TN are diseases of the central nervous system (CNS) and peripheral [2]. Neurodegenerative Diseases (ND) are found within TN, among other diseases. It is estimated that in Spain between 6 and 7.5 million citizens suffer from some type of NT (13-16% of the total population): around one and a half million with a serious neurological disease [3]. NEs have great consequences on the public health system and society. “To the disease process itself, we must add the psychological impact, the decrease in quality of life, the inability to work, the loss of social skills, the burden on caregivers and dependency situations” [4]

A study carried out by Neuroalianza and the Complutense University of Madrid [5] (Study on NE in Spain and its economic and social impact) indicates that each patient spends on average more than 23,000 euros per year.

*Table 1: Global prevalence of the three main neurodegenerative diseases. [5]*

| **Disease** | **Cases per 100.000**  **Hab** | **Prevalence** | **Affected population** | **Reference** |
| --- | --- | --- | --- | --- |
| **Alzheimer and other dementia** | 400 | 0,5% | 35.600.000 | (Prince et al., 2013, p. 63) |
| **Parkinson disease** | 315 | 0,34% | 23.800.000 | (Pringsheim, Jette, Frolkis, & Steeves, 2014, p. 1586) |
| **Multiple sclerosis** | 30 | 0,03% | 2.280.000 | (Trisolini, Honeycutt, Wiener, & Lesesne, 2010, p. 6) |

*Table 2: Prevalence of neurodegenerative diseases in Europe (EU-27). [5]*

| **Disease** | **Cases per 100.000**  **Hab** | **Prevalence** | **Affected population** | **Reference** |
| --- | --- | --- | --- | --- |
|  | **Hab** |  | **EUROPE** |  |
| **Alzheimer and other dementia** | 854 | 1,23% | 6.341.179 | (Gustavsson et al., 2011, p. 729) |
| **Parkinson disease** | 168 | 0,24% | 1.249.312 | (Gustavsson et al., 2011, p. 729) |
| **Multiple sclerosis** | 108 | 0,1% | 801.900 | (MSIF, 2013, p. 9) |
| **Neuromuscular diseases** | 29 | 0,041% | 149.079 | (Olesen et al., 2012) |
| **Sclerosis Lat. Amyotrophic** | 2-5 | 0,002% -  0,005% | 10.281-25.703 | (Camacho et al., 2014; Gustavsson et al., 2011) |

NDs such as Alzheimer's and other dementias, Parkinson's disease (PD), multiple sclerosis (MS), amyotrophic lateral sclerosis (ALS) and neuromuscular pathologies represent a total cost of 32,372 million euros annually in Spain, where people live with 988,000 people have this type of problem. 40% of those affected stop working due to the disease, and 53% have economic difficulty because of it (this without taking into account that today we would have to add the effects of COVID-19). PD is the second most prevalent NC today, after Alzheimer's [6], and belongs to the so-called Movement Disorders. It is a chronic disease, and is characterized by the loss (or degeneration) of dopaminergic neurons in the substantia nigra pars compacta (SNpc) of the midbrain. This loss causes a lack of dopamine in the body, which affects the control of movement, giving rise to typical motor symptoms, such as tremor at rest or rigidity, slowness in voluntary movements, difficulty pronunciation when speaking or muscle weakness. Although today it is still complex to classify the PD, and there is still a long way to go to investigate its pathophysiology, which is why it is also identified as one of the diseases within the α-synucleopathies, due to the pathological deposition of α-synuclein in the cytoplasm of neurons or glial cells. [7]. These deposits form intracellular inclusions called Lewy bodies [8], which together with dopaminergic degeneration of the SNpc form the pathological basis of PD.

FA has a very important role and a great impact on cortical activity in PD patients [9]. In his study, Carvalho was able to confirm that “during the execution of physical exercise, more cortical areas were activated, and the main hypothesis of this phenomenon is the increase in cerebral blood flow during exercise.” In this study, the motor symptoms of PD (UPDRS-III) were evaluated, comparing 3 groups of patients, where 2 groups performed different PA programs, in contrast to a group that performed physical therapy. Both groups that performed PA improved their motor symptoms by 27.5% (assessed Pre and Post with UPDRS-III), while the physiotherapy group showed an improvement of 2.9%. At the end of the intervention, all three groups improved their functional capacity.

This research arises from the need to connect cell biology research, especially those that refer to mitochondrial dysfunction and oxidative stress (common characteristics of several NEs), together with metabolic bioenergetic processes; and the symptoms presented by patients with PD in different physical activity (PA) programs.

The relevance of skin fibroblasts as a surrogate pattern in this research is given by their ability to reflect the typical biochemical deficits of nigral neurons [10]. Going forward to the “theoretical framework,” we quote Milanese who tells us that these deficits “are factors that support the neurodegenerative process of PD, and can be detected in fibroblasts from patients with sporadic Parkinson's Disease (PD).” Hence the importance as a surrogate biomarker for pathogenic processes, since it would be possible to correlate with clinical measures, such as motor function through the MDS-UPDRS III. Therefore, we seek to correlate and understand what processes occur at the mitochondrial level due to PA, which causes motor and non-motor symptoms to improve and/or slow their progression after an intervention.

There are recent studies (Deus et, al; 2020) in which skin fibroblasts from patients with PD were analyzed (non-neuronal cells) to detect metabolic and mitochondrial alterations that would also exist in a type of non-neuronal cell [11]. Studies like the one we just mentioned are those that have broadened the path of our research, and have further reinforced the certainty of using skin fibroblasts in patients with PD; and thus, be able to investigate the benefits of physical exercise for mitochondrial health in PD. All this has led us to begin research on the effects of PA, and the analysis of skin fibroblasts, in order to assess the mitochondrial function of patients; and also, how PA influences cellular proteolytic and bioenergetic deficits, present in every neurodegenerative process of PD [10].

This research aims to be crucial in assessing mitochondrial function in a way that has not been done before, i.e., before and after physical activity (AF) intervention in patients with Parkinson's disease (EP). Mitochondria have the ability to oxidize substrates and produce energy in the Krebs cycle and the electron transport chain (ETC). When mitochondrial metabolism of substrates is more efficient, energy production is optimized, and a greater amount of lactate is oxidized within them [12]. Mitochondria consume most of the oxygen (90%, according to R. A‐Perez 2020) through ETC Complex IV [13]. The entire complex mechanism of the ETC is essential for cellular metabolism dependent on these organelles. The ETC transforms the energy from nutrients and creates a route of proton and electron gradients to produce ATP.

The importance of mitochondria is primarily due to their function; they act as an energy factory in almost all cells and regulate the production of reactive oxygen species (ROS) and cell death processes. They do this by combining oxygen with fuel molecules (sugars and fats). This is why we are interested in measuring oxygen consumption in skin fibroblasts, known as respirometry, which aims to measure mitochondrial function. This is what N. Carter calls the gold standard measurement [14].

In Parkinson's disease, mitochondrial function is affected, cells do not have enough energy, and unused oxygen and fuel molecules accumulate in cells, causing damage. Muscle and nerve cells have high energy needs, so muscle and neurological problems are common. In healthy individuals, it is known that higher mitochondrial efficiency reduces the production of free radicals and maintains the integrity of proteins, lipids, and mitochondrial DNA [15]. Considering that mitochondrial function is a fundamental reference for understanding metabolism in health and disease. One of the "bridges" we find (among the multiple connections found) in the triad Mitochondrial Function-Parkinson's Disease-Physical Activity is mitochondria. Therefore, in the early stages of this research plan, we have delved into health, focusing on normal physiological values and the various pathways and impacts of physical activity. We have seen that there is a relationship between mitochondrial biogenesis and physical exercise. For example, one of the adaptations produced by aerobic training is the increase in the number and size of mitochondria.

Hoppeler (1985) demonstrated that with aerobic training lasting 6 weeks, 5 times a week, 30 minutes each session, with an intensity of 4 mMol of lactate, there was an average increase of 40% in these organelles, both subsarcolemmal and intermyofibrillar [16]. In trained subjects, twice the number of mitochondria per mm3 was found compared to untrained subjects. It is noteworthy that the signal to initiate mitochondrial biogenesis is muscle contraction; mitochondria will not increase if the muscle fiber is not recruited. This is one of the reasons why we have chosen, as a cross-cutting objective in both training programs, not only aerobic work but also strength training. The objectives are to improve the reduction of oxidative stress and the increase in antioxidant capacity.

On the other hand, the relationship between physical activity and certain positive neurological aspects [17] suggests that the exercise of the human body "appears to activate a series of processes responsible for maintaining and protecting nerve cells, which we can call physiological neuroprotection systems." Physical activity promotes the occurrence of compensation mechanisms through a reorganization of damaged neural circuits. Vera Hinojosa and Flores [18] explain that "physical activity is necessary in the human body to support a series of basic functions because, for the movement of the body through exercise, the brain is activated in multiple areas, basically associated with: the coordination of the correct movement of the muscles involved in the exercise, production and release of Myokines [19]; the increase in blood flow; the administration of glucose consumption; control of breathing and heart rate, the capacity of the sensory system, among many others [14]. Therefore, a sedentary lifestyle is a risk factor for many chronic pathologies. It is vital to increase levels of physical fitness in people with neurological diseases, particularly in individuals with Parkinson's disease (PD). This leads us to study the effectiveness of physical activity as a therapeutic intervention and to investigate how the benefits of exercise influence mitochondrial function in this type of population.

Having outlined the three main areas of knowledge that interact in this thesis (Exercise Science - Health Sciences (PD) - Cellular and Molecular Biology) and the brief introduction regarding the prevalence of PD, we can only add in this section the role of Biomedicine. Dr. Soria Bernat [20] anticipates that biomedical research in Spain will play a significant role in the scenario of major advances in this century. Despite the current growing demand for scientific research, Dr. Soria has warned us about the "discrepancy in the position that Spain occupies in Europe from an economic point of view and the place it occupies in research, in general, and in biomedicine, in particular" [20]. Currently, initiatives to counteract this discrepancy are based on promoting and developing research in Biomedicine that includes synergies and collaborations between universities, institutions, and other organizations such as the Carlos III Health Institute and the Biomedical Research Networking Centers (CIBER). These actions and such centers have allowed Spain to reduce the gap compared to other European countries. Quoting Dr. Soria, "it is essential that the result of knowledge reaches clinical practice and becomes effective therapies." Thus, to achieve the result of knowledge, this thesis proposes the path of physical activity in its form of therapeutic exercise to analyze its effectiveness (improvement of mitochondrial function) in close relation to clinical practice and to be returned to society in effective approaches for patients with PD. The unraveling of this knowledge can also serve as a reference for other neurological diseases.

# The topic and the objectives

**Thesis topic**

The theme of this thesis involves analyzing the effects of the intervention of two different physical activity (PA) programs in patients with Parkinson's disease (PD). The goal is to understand the repercussions of the benefits of physical exercise at the mitochondrial level, in order to compare with mitochondrial dysfunction in sedentary patients (control group).

It has been demonstrated that physical exercise is a therapeutic tool that slows down the pace of deterioration and progression of Parkinson's disease (PD). Given that mitochondrial dysfunction is present in the pathophysiology of PD, we have identified the next step in research, following the study by Deus et al., 2020 [11], as investigating, using skin fibroblasts as an assessment tool, but in this case with patients who have followed a physical activity (PA) plan. Our goal is to analyze the metabolic pathways of exercise. Scientific evidence has shown that the cellular energy state controls many metabolic reactions (Gomes et al., 2011) and that physical activity has beneficial effects on this energy state and, consequently, on its metabolic reactions.

To the best of our knowledge, this is the first research study examining the effects of supervised PA on mitochondrial function, using skin fibroblasts as biomarkers for comprehensive mitochondrial respirometry, in individuals with PD.

**General Objective:**

To assess the effects of physical activity on mitochondrial function in patients with Parkinson's disease who undergo different physical activity programs, and its impact on motor function, quality of life, sleep, cognitive aspects, and mood.

**Specific Objectives:**

- Assess Potential Changes in Proteolytic and Bioenergetic Cellular Deficits:
  - Evaluate potential alterations in proteolytic and bioenergetic cellular deficits resulting from different physical activity (PA) programs.
- Correlate Clinical Presentation with PD Patients' Evolution in Various PA Programs:
  - Relate the clinical manifestations of patients with Parkinson's disease (PD) to assess their evolution in different PA programs, considering the Hoehn and Yahr stages and motor function (MDS-UPDRS III).
- Evaluate Positive Effects of Improved Physical Fitness and Cardiovascular System on Multiple Aspects:
  - Assess the positive effects of enhanced physical fitness and cardiovascular health on motor function, quality of life, sleep, cognitive aspects, and mood.
- Determine Optimal PA Programs Through Literature Review:
  - Conduct a literature review to determine the most beneficial PA programs for improving mitochondrial function, motor function, quality of life, sleep, cognitive aspects, and mood in patients with PD.
- Conduct Metabolic Characterization of Cutaneous Fibroblasts in PD Patients:
  - Perform a metabolic characterization of cutaneous fibroblasts from patients with PD.
  - Evaluate the effects of metabolic remodeling through exercise on oxidative stress, mitochondrial quality control, mitochondrial DNA copy number, proteins, and transcripts in cutaneous fibroblasts of PD patients.
- Correlate Mitochondrial Function in Skin Fibroblasts as a Non-invasive Biomarker:
  - Establish correlations in mitochondrial function among skin fibroblasts from PD patients.
  - Develop a minimally invasive biological biomarker for the diagnosis and progression of PD based on mitochondrial function in skin fibroblasts.

# Theoretical Background and Current State of the Topic

In the context of our research and regarding the clinical approach to Parkinson's Disease (PD), we focus on one of the three areas of clinical intervention, which is Physical Activity (in the form of therapeutic exercise). The other two areas are Pharmacological and Surgical interventions. Currently, there is a significant boom and increased progress in studies of cellular and molecular biology, mitochondrial mechanisms (close relationship between structure and function), signaling, and metabolic processes. We have already made progress in sections 2 and 3 ("Justification" and "Topic"), providing important data regarding the state of the issue. In Bloomer's conclusions [21], there is a call to investigate in this area, as they are surprised that no study to date has explored the role of structured exercise in improving oxidative status in people with PD. Bloomer focused on studying the blood-level effects of resistance training on oxidative stress in PD.

So far, no studies have been found that relate the clinical aspects of PD, using cellular organelles (through fibroblasts) to analyze the impact of different types of Physical Activity (PA) and how they influence deficits in cellular bioenergetics. This is the first research that will study the effects of PA on mitochondrial dysfunction, using the study model of skin fibroblasts in people with PD. "Skeletal muscle functions as an endocrine organ; it can produce and secrete hundreds of myokines with autocrine, paracrine, or endocrine signals. Recent advances show that skeletal muscle produces myokines in response to exercise, allowing cross-talk between muscle and other organs, including the brain. Currently, it has been identified that the biological functions of myokines have effects on cognition, lipid metabolism, glucose, among others. In relation to this thesis, it suggests that myokines can be useful biomarkers for monitoring the prescription of PA for people with PD [19]."

The study of the human nervous system is complex and challenging due to the inaccessibility of tissue, coupled with the limitations of cellular and animal models to fully reproduce its physiology. Concerning research on molecular mechanisms related to aging and neurodegenerative diseases (EN), genetically modified animals have been used for years, but they fail to fully replicate the pathologies or phenotypes associated with aging. Quoting part of the conclusions of Ambrosi's study [10], "research on pathogenic mechanisms in peripheral cells, such as fibroblasts derived from patients with sporadic PD and age/sex-matched controls, could provide a deeper understanding of deficits affecting dopaminergic neurons and possibly new tools applicable to clinical practice." Thus, in the midst of this conclusion, our research aims to incorporate/contribute a new variable, which will be analyzed through fibroblasts, to obtain new conclusions/results and provide cellular and molecular evidence of how different PA programs influence patients with PD.

In the literature review (state of the question and theoretical framework), we have found a common denominator [10, 22] in research using cutaneous fibroblasts from PD patients. All agree that it is an easily accessible source of proliferating cells, sharing the same genetic complexity as neurons [23], showing typical biochemical deficits of nigral neurons. These deficits are related to sustaining the neurodegenerative process of PD and are also capable of reflecting cumulative cellular damage with the patient's age [10]. Several authors suggest that research on peripheral biomarkers could be based on cutaneous fibroblasts from PD patients [10, 24].

Milanese and colleagues in their research [24] argue that there is a relationship between the clinical picture of patients with Parkinson's Disease (PD) and peripheral mitochondrial function. This provides a key point in the justification of this research in the doctoral program.

# Hypothesis and Research Question

- Null Hypothesis: Physical exercise will not be beneficial in improving the function of mitochondrial metabolism in skin fibroblasts in patients with Parkinson's Disease (PD), and it will not have positive effects on motor function, cognitive and emotional aspects such as mood, as well as quality of life and sleep.
- Alternative Hypothesis: Physical exercise will be beneficial in improving the function of mitochondrial metabolism in skin fibroblasts in patients with PD, and it will have positive effects on motor function, cognitive and emotional aspects such as mood, as well as quality of life and sleep.
- Research Question: Can physical exercise restore and/or improve the function of mitochondrial metabolism in skin fibroblasts of patients with PD, and can it also improve motor function, cognitive and emotional aspects such as mood, as well as quality of life and sleep?

# Design

**Metodology: Method and Instruments**

Methodological Rationale: From the outset, this research has predominantly adopted a quantitative methodology. The research design falls within an Analytical-Experimental framework, being prospective (Design and General Publication Plan derived from the thesis, page 23). Quantitative methodology is involved in each of the main assessments of this research, both for the primary objective and the secondary objectives. Regarding mitochondrial assessment, respirometry is chosen as the "gold standard," based on the results generated by Seahorse analyses processed with Wave software (Agilent). The essence of the methodological rationale through quantitative methodology lies in the fact that the evaluation of the impact of Physical Activity (PA) on skin fibroblasts relies on laboratory tests using primarily chemical, physical, and biochemical means. This approach allows for the direct or indirect measurement of various bioenergetic processes that can detect biochemical alterations for comparison pre and post-intervention.

Motor function assessment is also a clear example of the quantitative aspect, utilizing the MDS-UPDRS III scale.

**6.1. Method:** Two different types of PA programs will be implemented. The first will focus solely on basic physical fitness components (BPF), specifically targeting Strength (S) and Endurance (E). The second program will consist of BPF (S and E will be worked on transversely in both programs) + stimulation of specific sensorimotor pathways that are more affected (Proprioception-Balance-Coordination). This program adds a cognitive-motor component ("dual-task training"), involving walking back and forth with coordinative exercises. At the extremes, a cognitive activity will be performed.

*Table 3: Design of the physical activity intervention*

**Subjects**

-Participants:

-Group:……..

**BPF MIXED PROGRAM: STRENGTH-RESISTANCE** (Reference+Synergy = What yielded more positive results in the cited references, plus others to be analyzed during the literature review, will carry more weight in the macro-meso-micro cycle).

##### Reference+Sinergy Description Results

-EP (HY I-III) CFB: …...(±DE) CFB-EF: ……(±DE)

-age: …… ± ……

-Duration EP:

…… ± ……

- MMSE: …… ±

-Patients in mode “on”

**-Miyai et al.,2000 [25]**

***Pros:**

1. **El estudio en corto plazo, BWSTT mejora más la movilidad que AG.**
2. **BWSTT fue más efectivo para mejorar las puntuaciones UPDRS, velocidad de marcha y longitud de paso**

**Steffen etal., 2012 [26]**

***Contras: Intervention: 10 months**

***Pros:**

**-Improved the mental state, behaviour, and humour from UPDRS**

-**Body weight–supported treadmill training** BWST

- **General conditioning,** range of motion exercises, training for activities of daily living, and gait training.

Training Program:

Forward treadmill walking: Enhanced endurance, speed, and stride length. Treadmill training speed: between 2.7 and 4.8 km/h, slope 0%.

Backward treadmill walking.

Mat activities for mobility and strengthening of the hip and spine.

-Quality of life-PDQ-39

-MDS-UPDRS-III (motor exam) functional assessment:

-Endurance and walking speed: 6MWT

-Equilibrium: BBS

-Functional mobility: TUG

Rendimiento funcional:

-Resistencia y velocidad marcha: 6MWT

-Equilibrio: BBS

-Movilidad funcional: TUG

- The sessions will be part of the main program and Will be adapted by the investigator.

CFB: Basic Physical Fitness DE: standar deviation

**BPF-EF MIXED PROGRAM + FUNCTIONAL EXERCISES** (Reference+Synergy = It will carry more weight in the macro-meso-micro cycle, having yielded more positive results in the cited reference.

.

**Gobbi et al.,2009 [27]** Multiple mode exercises

-Functional capacity, aerobic capacity, flexibility, upper and lower limb strenght, coordination and balance.

-Functional mobility-TUG

-Functional equilibrium- Berg Scale (FBS)

-MMSE -HY -UPDRS

For this reason and COVID measures, the ration Will be 5:1

**Tanaka et al., 2009 [28]**

**Physical exercise program conducted over 6 months focusing on Coordination-Strength-Balance. Texeira et al., 2014, also conducted the study in 6 phases.**

-Sessions with an aerobic component

-Stretching (flexibility), muscle strength, coordination, and balance were addressed through recreational motor activities.

-The intensity increased at the end of each phase.

Improvement in executive functions was evident.

CFB-EF: Basic Physical Fitness - Functional Exercises

The two physical activity programs will be compared with the control group. It will be determined which of the programs provides more positive changes in mitochondrial function, cellular bioenergetics, and all relevant information from cutaneous fibroblasts. Taking into account the evolution in the clinical presentation of each patient, including motor function, cognitive aspects, mood, quality of life, and sleep. Tests will be conducted at the beginning (Pre-intervention), in the middle, and at the end of the intervention (2 and 4 months), and physical-functional tests at 8 months. Measurements and intervention will be carried out with patients in the "on" state.

In the methodological part concerning cutaneous fibroblasts and certain clinical and laboratory controls, the first phase in Spain will be conducted under the supervision of the Principal Investigator (PI)-Spain and co-direction of the thesis by Dr. Joel Montané, at the FCS-Blanquerna of the Universitat Ramon Llull (URL) and at the collaborating centers of the Neurodegenerative Diseases Group of the Vall d’Hebron University Hospital-Institute of Research (VHIR), Barcelona. The supervision will be provided by Dr. Jorge Hernández Vara (Neurologist), with the collaboration of neurologist Daniela Samaniego Toro, and researchers Dr. Marta Martínez-Vicente and Dr. Ariadna Laguna Tuset. Biopsies of the skin will be performed at this center by one of the neurologists from the VHIR Neurodegenerative Diseases Group (at the beginning and end of the intervention in the 3 groups), and fibroblast amplification will be carried out. The subsequent cultivation and processing (second phase in Portugal) will be carried out by the Center for Neuroscience and Cell Biology (University of Coimbra), where the PI-Portugal and co-director of this thesis, Dr. Susana P. Pereira, is one of the principal investigators of the center. In this second phase (Portugal Phase), there will be an International Center Stay (ECI) by the doctoral student (Mobility of the doctoral student, stays in research centers).

**Fibroblast Protocol:** The general plan is to collect samples in Spain, isolate fibroblasts, and expand them at VHIR (3 million cells), sending 1 million cells to Portugal.

**Spain Phase:**

- Collect skin biopsies, isolate fibroblasts, and cultivate to expand (increase the number of cells) to obtain 3 million cells (Dr. Susana P. Pereira). Specialized transportation will be handled by a dedicated company (Quotes requested from FedEx, Polar-Express, and Lab-courier).

**Portugal Phase (General Description):**

- With the cells in Portugal, they can be re-cultivated and expanded for experiments, making it possible to increase the number of cells for various experiments and freeze them if necessary.
- Seahorse analyses will be conducted, providing mitochondrial function (parameters in Fig.1 in the Milanese 2019 article) [24].
- Mitochondrial potential will be determined using the TMRM probe, cellular oxidative stress (e.g., using Amplex Red and other probes), and activities of antioxidant enzymes or protein content and/or mRNA levels.
- Mitochondrial dynamics, quality control of proteins, evaluating the efficiency of the Ubiquitin Proteasome System (UPS), protein levels of autophagic markers, and changes in cellular bioenergetics will be assessed.
- Based on the results, specific pathways related to exercise can be investigated.

Version 4, 05/12/2022

##### PHASE ESPAÑA


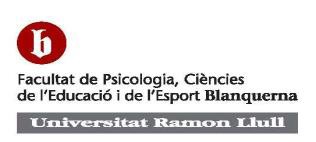

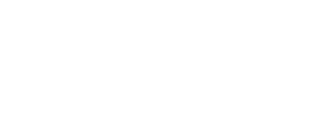

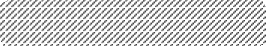

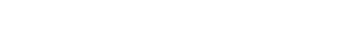

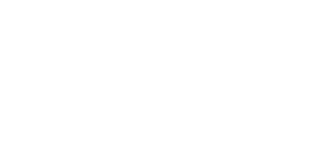

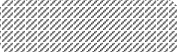

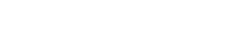

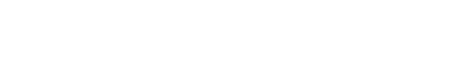

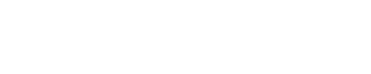


**PHYSICAL ACTIVITY PROGRAMS + FIBROBLAST EXTRACTION + EVALUATIONS**

**FASE ESPAÑA**

(IP-Spain) y

Co-supervisor

Dr. Joel Montané

-Mesocicle 1

-Mesocicle 2

-Mesocicle 3

-Mesocicle 4

##### MACROCYCLE OF PHYSICAL ACTIVITY PROGRAMS

|  | | | | | | | | | |  | | | | | | | | |
| --- | --- | --- | --- | --- | --- | --- | --- | --- | --- | --- | --- | --- | --- | --- | --- | --- | --- | --- |
|  |  |  |  |  |  |  |  |  |  | |  |  |  |  |  |  |  |  |
| *Initial evaluation* | *Week 1* | 2 | 3 | *4* | *5* | *6* | *7* | *8* | *9* | | *10* | *11* | *12* | *13* | *14* | *15* | *Week 16* | *Final evaluation* |

-Initial and Final: Extractions of samples and questionnaires and evaluations

3 groups

-Intermediate evaluation: Only physical tests

| **Functional evaluation and Physcal activity test** | **Motor evaluation MDS-UPDRS III** | **Health evaluation** | **Fibroblast extraction** | **Questionnaires** |
| --- | --- | --- | --- | --- |
| - 6-Minute Walk Test - 1-Minute Sit-to-Stand - Timed Up and Go (time to stand up from a chair, walk 3 meters, turn around, walk back, and sit down) - Grip Strength Test | -Motor evaluation MDS-UPDRS III | - Blood pressure, heart rate - Height-weight-BMI - Anthropometric measurements - Neurological examination (Strength, reflexes, sensitivity) - Orthopedic and neurodynamic tests of upper and lower extremities (UE-LE). | -Fibroblast extraction | - (MoCA)  - Depression Beck-IDB  -PD-CRS, Quality of life, Humour y Sleep (SCOPA-AUT, NMS, PDSS, PDQ39,  Test of BERG, TINETTI and fall Questionnaires) |

FASE

(IP-Portugal) y

Co-supervisorDay 1.A: Evaluación funcional y Test AF FCS-Blanquerna

Day 1.D: Motor evaluation MDS-UPDRS III VHIR

Day 1.B: Health evaluation FCS-Blanquerna

Day 1.E: extraction of Fibroblasts-

Hospital Universitario-VHIR

Day 1.C:

Questionnaires-VHIR

PORTUGAL

Dra. Susana P. Pereira

**Hospital Universitario-VHIR**

**Extraction of Fibroblasts-**

**VHIR**

- Expansion and cryopreservation of fibroblasts.

***Functional evaluation:** Miokines in response to physical activity [19]

**Centro de Neurociencia y Biología Celular (CNC)- Universidad de Coimbra- Portugal**

**Empresa de transporte**

**especializada**

Valoración Mitocondrial

- Isolate fibroblasts, cultivate them to expand (increase the number of cells) until reaching 3 million cells (per collected sample).

Rutas metabólicas del ejercicio en EP


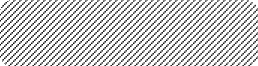

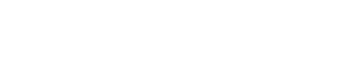


**Instruments:**

*Table 4.1: Study Variables and Measurement Instruments.*

| **Study variables** | **Measurement instruments** |
| --- | --- |
| **Muscular strength MMSS y MMII** | -dynamometer. |
| **Functional evaluation - equilibrium** | -Test of Berg (BBS), TINETTI a fall questionnaire |
| **Functional evaluation - gait** | -6-minute walk test (6MWT). |
| **Functional evaluation -STS** | -Sit-To-Stands in one minute |
| **Functional evaluation -TUG** | Timed Up and Go |
| **Motor function** | The Unified Parkinson's Disease Rating Scale (UPDRS) |
| **Cognitive aspects** | -MMES - PD-CRS -Clock Drawing Test (CDT) -MoCA  -Trail Making Test A (TMT-A) and B (TMT-B). |
| **Quality of life** | - Parkinson’s disease questionnaire (PDQ-39). |
| **Humour** | -Humour BRUMS. |
| **Sleep** | - Sleep (SCOPA) - Parkinson's disease sleep scale (PDSS) (Chaudhuri et al, 2002). |
| **Respirometry**-**cellular oxygen consumption** | -Oxygen consumption rate (OCR) y extracellular acidification rate (ECAR) using Seahorse XF. |
| **Depression** | Depression test Beck-IDB |
| **Fibroblast characterization** | -Seahorse-Se analyxix using Wave (Agilent) software |

*Table 4.2.: Objectives, Description, and Experiments of the Fibroblast Protocol - PORTUGAL PHASE - CNC*

| **General objective** | **Specific objectives** | **Description** | **Mitocondrial biology instruments** |
| --- | --- | --- | --- |
| Evaluate Mitochondrial Remodeling in Skin Fibroblasts of Patients with Parkinson's Disease (PD) undergoing Physical Activity Intervention, and respective age-matched controls. | Evaluate the effects of metabolic remodeling on oxidative stress, mitochondrial quality control, mitochondrial DNA copy number, proteins, and transcriptions. | In this task, we will characterize the effects of exercise-induced remodeling in fibroblast cells of patients with PD in terms of epigenetic changes, mitochondrial biogenesis, oxidative stress, and mitochondrial quality control. | a) qRT-PCR for transcripts of interest related to mitochondrial biogenesis, dynamics, epigenetic regulation, oxidative phosphorylation, oxidative stress (SOD1, SOD2, NFE2L2), quality control mechanisms, including autophagy and UPS. b) Mitochondrial transcripts, RT-PCR will be performed using SsoFast Eva Green Supermix on a CFX96 Real-Time PCR System (Bio-Rad, Hercules, CA, USA). c) Regulation and abundance of mitochondrial proteins, immunoblotting using the Trans-Blot Turbo Transfer System (Bio-Rad) and UVP BioSpectrum 500 Imaging System (UVP, Upland, California). d) Enzymatic activities, a multimode microplate reader Cytation3 (BioTek Instruments, Inc.). e) Complex I and citrate synthase activity using colorimetric methods. |

**6.2 Sample: Selection Criteria (Inclusion, Exclusion, and Withdrawal Criteria), Recruitment** **6.2.1 Sample:** The sample size has been calculated using the GRANMO program with the observed proportions model relative to a reference. The calculation takes into account that "maximal respiration" is key to assessing the effects of physical exercise (PE) on mitochondrial function because it forces the entire respiratory chain to the maximum. The calculation is based on accepting an Alpha risk of 0.05 and a Beta risk below 0.2 in a bilateral test. Based on preliminary data, there is a difference in "maximal respiration" equal to or greater than 0.263 units [11]. It is assumed that the proportion in the reference group is 0.019 in individuals over 50 years old [5]. As an interventional study, the percentage of necessary replacements is expected to be 20%. Therefore, 8 subjects per group are needed, totaling 24 patients for the study. Patients with PD will be recruited from ages 50 to 70 if they meet inclusion criteria (patients with good cognitive status, Montreal Cognitive Assessment (MoCA) score greater than or equal to 26, patients who have signed informed consent, patients capable of walking independently for 6 minutes, and patients with stable medication (no changes in the last month). MDS-UPDRS-III (motor subscale) score and Hoehn and Yahr stage (up to stage 3) will be determined and assessed in the "ON" phase. The sample will be divided into three groups of stable patients after the first evaluation: the first group (n=8) will work on basic physical capacities, the second group (n=8) will work on basic physical capacities + stimulation of specific sensorimotor pathways + "dual task training" + cognitive activity, and the third will be the Control Group (n=8). Once the first evaluation is completed, randomization by block (see Randomization Annex p. 58) of the sample into the three explained groups will be conducted. Randomization will be based on PD stages (stages I, II, III of Hoehn and Yahr), gender, and age.

*Table 5:* ***Hoehn & Yahr Staging Scale for the Progression of Parkinson's Disease:***

| **STAGES** | **Description** | **CLASIFICATION** |
| --- | --- | --- |
| **Stage I** | The descriptions you provided seem to outline different stages or levels of motor impairment in individuals with Parkinson's disease. Here's a brief translation: | Recently diagnosed patients |
| **Stage II** | Exclusively unilateral involvement | Recently diagnosed patients |
| **Stage III** | Bilateral involvement without balance impairment | Moderately affected patients |
| **Stage IV** | Bilateral involvement with balance impairment | Patients with severe disability and an increase in the degree of dependence |
| **Stage V** | Still able to walk or stand without assistance | Severely affected patients |

**6.2.1 Inclusion Criteria:**

- Patients with a medical diagnosis of idiopathic PD, stages I-III on the Hoehn & Yahr scale [29].
- Patients with good cognitive status, scoring equal to or greater than 26 on the Montreal Cognitive Assessment (MoCA).
- Patients who have signed the Informed Consent (IC).
- Age between 50 and 70 years, with the ability to walk independently for six minutes.
- Patients with stable medication (no changes in the medication in the last month).

**6.2.2. Exclusion Criteria:**

- Patients with a pathology other than idiopathic PD.
- Patients with cognitive impairment (MoCA score <26 points).
- Patients with uncontrolled cardiovascular disease, visual impairment, or recent musculoskeletal disorders in the upper or lower extremities that could interfere with balance and locomotion.
- Patients currently undergoing another therapeutic exercise protocol.
- Patients who have undergone surgery with the aim of influencing specific PD symptoms.

**Withdrawal Criteria:** Voluntary withdrawal of subjects from the study at any time and any complications that may arise during the intervention are considered reasons for withdrawal.

**6.2.3 Recruitment:** Recruitment will be progressive and continuous, depending on the speed of subject incorporation. Dr. Jorge Hernández Vara, Associate Physician of the Neurology Service (Movement Disorders Area) at HUVH, will be in charge, in coordination with collaborating centers and the involvement of the doctoral student. To ensure a minimum recruitment of 24 patients as established in the project, VHIR researchers will use their contacts with the Catalan Parkinson's Association and other Movement Disorder Units in major hospitals nationwide.

- Implement two lines of physical activity, with three classes per week for four months, one for each of the groups that will engage in physical activity.

**6.2.4 Procedure:** Firstly, each participant, who has previously read and signed the informed consent, will be interviewed. During the interview, the medical history will be consulted through a complete anamnesis, obtaining data on their current health status and the chronology of their particular condition. The medical history and neurological examination will be conducted to confirm inclusion-exclusion criteria (cranial nerves, sensitivity, motor strength, reflexes). Before the intervention, two familiarization sessions will be held. Participants will get to know the staff working with them (assistants and researchers), the places where they will be evaluated, and where the intervention will take place. They will also become familiar with the laboratory equipment, the materials used in exercise practice, and the various evaluation techniques.

**Initial Assessment (to be conducted by both intervention groups + control group):**

1. Verification of the general health status, analyzing the fitness of each participant for engaging in physical activity.
2. A battery of field tests (described in the Functional and Motor Assessment-General Flowchart of the Research (page 13)).
3. Anamnesis and neurological examination (cranial nerves, sensitivity, motor strength, reflexes).
4. Orthopedic and neurodynamic tests for upper and lower limbs (UE-LL).
5. Psychological tests to assess cognitive aspects, including the Montreal Cognitive Assessment (MoCA), the self-applied scale for depression assessment (Beck Depression Inventory; BDI), to exclude subjects with depressive disorders (scores of 10-13/15). The final score will be evaluated with Neuropsychologist Catalina Pons Marquès, and alternatively, the Self-Rating Depression Scale (SDS) by Zung. [30, 31, 32] Also, the Parkinson's Disease Cognitive Rating Scale (PD-CRS) created by neurologists at Sant Pau Hospital. The Clock Drawing Test (CDT) is interesting for the differentiation it makes between cortical and subcortical functions. Trail Making Test A (TMT-A) and B (TMT-B) will be administered based on the sample characteristics and the degree of tremor in patients.
6. Quality of life, mood, and sleep tests.

This assessment will be repeated three times: at the beginning and end of the intervention and 4 months after completing the intervention (8 months from the initial assessment). The assessments will take place at the FCS-Blanquerna campus of Universitat Ramon Llull (URL) and/or collaborating centers. The logistics will be decided considering safety, maximum comfort, and minimal patient travel.

**6.3. Intervention:** The two designed programs will have a duration of 4 months. The interventions will be conducted in groups, with 8 patients per group (2 intervention groups + 1 control group), lasting for 60 minutes each, with a frequency of 3 times per week. These intervention programs will be structured into 4 meso-cycles of 4 weeks each. During the first 2 weeks, the workload will be maintained to favor the adaptation period, similarly in programs that require adaptation to both the load and tasks of complex motor execution.

There will be a progressive increase in workloads. Participants in the control group will continue with their usual daily practice and will be interviewed once a week by the researchers to ensure that their routines have not been altered. This control group will receive 4 months of physical activity from the program that has achieved the best results in terms of symptoms and quality of life after the last assessment (8 months).

##### Variables

- Respirometry
- Muscle strength of upper limbs (MMSS) and lower limbs (MMII)
- Balance
- Gait
- Endurance

Indirect variables

- Mitochondrial function
- Motor function
- Quality of life
- Cognitive aspects
- Sleep
- Mood
  1. **Data Collection**

The researcher will properly prepare the data collection area, minimizing potential risks and complying with health measures taken due to COVID-19.

In the Spain Phase, skin fibroblast biopsies will be collected to be cultured and expanded (increasing the number of cells) until obtaining 3 million cells (following recommendations from Dr. Susana P. Pereira), which will then be transported to Portugal. The participation of patients in this research is a significant contribution to the search for meaningful insights into the treatment and management of PD progression. Their commitment to participating in this study is not only beneficial for themselves but also for society (similar to a blood donation). Moreover, it is a contribution to the advancement of science, shedding light on the pathways of neurodegenerative processes to improve the quality of life for patients. Further evaluation data collected in Spain will be stored on the research team's computer devices, protected under a password.

With the cells in Portugal, Seahorse analyses will be performed, searching for autophagic measurements and possible changes in cellular bioenergetics. The rest of the information is detailed in the instrument section.

- 1. **Data analysis**

The analyses will be conducted using the statistical software SPSS v.20 (IBM SPSS Statistics). The following analyses will be specified with the Tutor and Thesis Director:

- Descriptive Analysis: All variables in the sample will be analyzed using relative and absolute frequencies, as well as measures of dispersion and central tendency.
- Repeated Measures Analysis: Mixed-effects model for repeated measures, whether the sample is homogeneous or non-homogeneous. This will allow analyzing potential changes between different moments of the intervention and evaluating which type of intervention provides more physical benefits, whether in factors related to mitochondrial function, physiological aspects, quality of life, or cognitive and emotional aspects.
- Spearman's rho and Pearson's r to evaluate the association between two variables with ordinal categories. The normality of the distribution of the results for each group will be assessed using the Shapiro-Wilk normality test, with α = 0.05 considered the threshold for passing the normality test. If the data show a normal distribution, a parametric paired t-test will be performed. Otherwise, the Mann-Whitney test will be used. Statistical test values with p <0.05 will be considered statistically significant differences.

# Ethics

This research is conducted following the principles of the Declaration of Helsinki and relevant national and international legislation, as well as the Charter of Fundamental Rights of the European Union and the European Convention on Human Rights. The study is based on the Anglo-American tradition of Ethical Principles of Research. Throughout the research, the privacy and physical integrity of individuals will be maintained, along with the confidentiality of the data collected for the research. Images will be taken exclusively for the purposes of this study, and faces will be pixelated to ensure that subjects are not identifiable. The obtained images will never be accompanied by data or information that could reveal the subjects' identity to third parties.

The research we have outlined adheres to the regulations referenced in the General Data Protection Regulation (GDPR), Regulation (EU) 2016/679, and data privacy and confidentiality (LOPDGD) Organic Law (3/2018 of December 5) on the Protection of Personal Data and Guarantee of Digital Rights. Informed consents and patient information are attached in annexes, as well as the specific section on Basic Principles of Ethics Applied to Research (Ethical Reflection on this research).

# ****Applicability, Limitations, and Future Directions****

**Applicability:** Quoting part of a conclusion from Ambrosi's study [10], "research on pathogenic mechanisms in peripheral cells, such as fibroblasts derived from patients with sporadic PD and age/sex-matched controls, could provide a deeper understanding of deficits affecting dopaminergic neurons and possibly new tools applicable to clinical practice." In line with this conclusion, our research aims to incorporate/contribute a new variable, which will be analyzed through fibroblasts, to obtain new conclusions/results on how different physical activity programs influence PD patients.

**Limitations:** Some limitations arise from questions we have asked, and whose answers have not yet been described in the scientific literature and/or have not been investigated. Questions such as, "What is the minimum time of physical activity to observe changes in fibroblasts?" or "What type of physical activity produces changes in the mitochondria of skin fibroblasts?" Currently, these answers are unknown, as tissues typically considered for this type of analysis in humans are primarily skeletal muscle or visceral fat. The new focus on fibroblasts is continually growing.

A potential limitation associated with fibroblasts may be that they do not show any phenotypic alteration associated with exercise, as this condition predisposes to greater mitochondrial capacity but does not guarantee that this difference is noticeable in a basal state (Susana P. Pereira). In science, today's limitations can be the frontiers we cross tomorrow. Therefore, we aim to overcome this limitation by challenging the mitochondria of skin fibroblasts, forcing cells to rely on oxidative phosphorylation (OXPHOS) metabolism to survive, which can be done using the OXPHOS medium [10].

We must not forget that we are in the midst of a pandemic (COVID-19), and access to healthcare centers, hospitals, and clinics is very difficult. We have already begun the protocol phase of searching and contacting professionals in clinical centers and hospitals (VHIR). Currently, we are evaluating various options for possible synergy strategies with other centers.

Other limitations could include "dropout" or losses, but this has already been taken into account in the sample calculation.

**Future Directions:** Future directions are related to the applicability to other neurodegenerative diseases (EN).

# 9. Chronogram


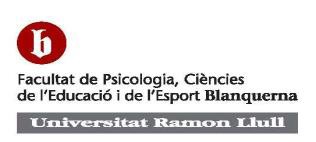


**2020 2021 2022 2023 2024**

Plan

| **7** | **8** | **9** | **1** | **1** | **1** | **1** | **2** | **3** | **4** | **5** | **6** | **7** | **8** | **9** | **1** | **1** | **1** | **1** | **2** | **3** | **4** | **5** | **6** | **7** | **8** | **9** | **1** | **1** | **1** | **1** | **2** | **3** | **4** | **5** | **6** | **7** | **8** | **9** | **1** | **1** | **1** | **1** | **2** | **3** | **4** | **5** | **6** | **7** | **8** | **9** | **1** | **1** | **1** |
| --- | --- | --- | --- | --- | --- | --- | --- | --- | --- | --- | --- | --- | --- | --- | --- | --- | --- | --- | --- | --- | --- | --- | --- | --- | --- | --- | --- | --- | --- | --- | --- | --- | --- | --- | --- | --- | --- | --- | --- | --- | --- | --- | --- | --- | --- | --- | --- | --- | --- | --- | --- | --- | --- |
|  |  |  | **0** | **1** | **2** |  |  |  |  |  |  |  |  |  | **0** | **1** | **2** |  |  |  |  |  |  |  |  |  | **0** | **1** | **2** |  |  |  |  |  |  |  |  |  | **0** | **1** | **2** |  |  |  |  |  |  |  |  |  | **0** | **1** | **2** |

Sistematic review

FASE 1 ESPA ÑA

FASE 2 POR TUG AL

Topic definition

Preparation of the study

Development of the investigation plan Revision of the scientific literature

Elaboration of the systematic review

Peer review and comments

Publication

Definition of PA programs Familiarization of the program by the investigators

Recruitment of participants Randomization

Initial evaluation Intervention Post test

Extraction Amplification and freezing of fibroblasts

Familiarization of the doctoral student with the researchers at CNC-UC.

Sample reception

1

2

3

Data collection and analysiss

PhD Objetives

Methods and results

Discusion

20 Conclusions

Versión 4, 05/12/2022

# 10. Resources

**10.1 Necessary Resources**

The materials required to carry out this study include:

1. **Information about CNC (now CIBB; cibb.uc.pt):**
   - The second Portuguese R&D unit in the field of life and health sciences.
   - Has three sites on the campuses of the University of Coimbra.
   - Mainly dedicated to research and health sciences, closely located to University Hospitals (CHUC) for translational research.
   - Equipped with advanced infrastructure, including the Seahorse XFe96 Analyzer for metabolic measurements, microscopy units for cellular analysis, and platforms like the GE In Cell Analyzer 2200 and Fluidigm Biomark HD for automated assays.
2. **Vall d'Hebron Research Institute (VHIR):**
   - More than 60 research groups and over 1,400 researchers dedicated to a wide range of diseases and improving patient care.
   - The Neurodegenerative Diseases Group, part of the Spanish Network of Research Groups in Neurodegenerative Diseases (CIBERNED), is recognized as a consolidated group by the AGAUR of the Generalitat de Catalunya.
   - Focuses on cutting-edge research in Parkinson's Disease, including neuromelanin and alpha-synuclein, lysosomal dysfunction, and immune system and gut microbiota alterations.
   - Well-equipped laboratory with necessary facilities for the proposed activities.
3. **Pre-doctoral contract (Doctorando) - Blanquerna:**
   - Contract for pre-doctoral research training.
4. **Continuous training and Membership in International Parkinson and Movement Disorder Society (MDS):**
   - Support for ongoing education and networking in the field.
5. **FCS: Room:**
   - Facilities provided by FCS for the study.
6. **Other necessary resources for AF material:**
   - Available at FPCEE for carrying out the intervention.

These resources ensure the study has the necessary support, infrastructure, and expertise for its successful implementation.

**10.2 Budget Distribution**

The budget distribution for the study includes the following components:

1. **Skin Biopsy Procedure:**
   - Estimated budget between 190 and 220 euros per patient and exploration.
   - Contact Person: Antonia Campolongo Perillo, Nurse at the Movement Disorders Unit, Hospital Sant Pau.
   - Phone: +34683524471, Internal Extension: 30127.
2. **Derivation, Expansion, and Cryopreservation of Fibroblasts:**
   - The process involves cryopreserving fibroblasts, either in liquid nitrogen or using alternative methods such as the Mrs. Frosty device to achieve freezing without crystal formation.
   - Ensures the viability of cells during cryopreservation.
3. **Isolation and Cultivation of Fibroblasts:**
   - Involves isolating fibroblasts, cultivating them to expand (increase the number of cells) until reaching 3 million cells per sample collected.

This budget distribution covers the costs associated with the crucial steps of the study, including the skin biopsy procedure and the handling of fibroblasts for further analysis and experimentation. It ensures that the necessary funds are allocated to each stage of the research process.

*Table 6: Budget for fibroblasts expansion Fase España-IBIDELL*

| **Private External Fee** | **For 1 sample** | **For 42 samples processed at 2 times** | **For 42 samples at once** |
| --- | --- | --- | --- |
| Derivation/Expansion/Cryopreservation HDF (for 1 sample) | 767,901 | 32251,827 | 32251,82693 |
| Test of Mycoplasma | 19,718 | 828,172 | 828,172 |
| Use of culture room (45 days) | 2639,364907 | 5278,730 | 2639,364907 |
| **Total cost** | **€ 143933,325** | **€ 38358,728** | **€ 35719,364** |
|  |  |  |  |

Teresa Zomeño, PhD Responsable de la Plataforma de Cultius Avançats de Cèl·lules i Teixits Serveis Cientificotècnics Tel: +34 93 316 03 17 Email: [actc@idibell.cat](mailto:actc@idibell.cat) Website: [www.idibell.cat](http://www.idibell.cat) Institut d'investigació Biomédica Bellvitge-Hospital Duran i Reynals Gran Via de L’Hospitalet, 199-08908 Hospitalet de Llobregat Barcelona

**Note:** The VHIR has accepted to be our collaborative center and partake in our research. Consequently, they will cover the expenses related to the skin biopsies and the initial phase of fibroblast cultivation, as indicated in the initial budget. This collaboration significantly contributes to the advancement of our research, and we appreciate the support provided by the VHIR in this scientific endeavor.

**10.3 POSSIBLE SOURCES OF FUNDING:**

- **Public Sources:** Competitive project proposals will be submitted to public institutions, such as Challenge projects that promote international cooperation in research, PERIS, among others.
- **Private Sources:** A search for potential private sources that currently fund similar projects will be conducted.
- **Presented Finances and Grants:** Applications for available financing and grants will be pursued.

| **Description** | **Funding agency** | **duration ción** | **Total** |
| --- | --- | --- | --- |
| **Convocatoria para la concesión**  **de Becas para la financiación de Proyectos de Investigación** | Fundación Eugenio Rodríguez Pascual (Solicitud presentada en Julio de 2021) | 1  año | 25.000 euros |
| **Ayuda a la actividad de investigación del personal docente e investigador de la URL** | Universidad Ramon Llull | 1  año | 20.000 euros |
| **Convocatoria Premio Especial a la Innovación en el Sector Sociosanitario.** | Fundación DomusVi | 1  año | 20.000 euros |

**10.4. Funding and Grants Obtained:**

- **Funding and Grants Received:** Assistance for the research activity of teaching and research staff at URL (Reference: 2021-URL-Proj-004).

# 11. Investigators

**Researchers, Mentoring, and Expert Opinion from Other Professionals:** This work has benefited from the experience and expert opinion of the thesis co-directors and the following professionals:

- Mentoring and expert opinion ("expert interviews") from Dr. Jaime Kulisevsky (Hospital Sant Pau), Director of the "Fundació Institut de Recerca de l'Hospital de la Santa Creu i Sant Pau."
- Nurse from the group of Dr. Jaime Kulisevsky: Antonia Campolongo Perillo, Nurse at the Movement Disorders Unit, Hospital Sant Pau.
- Neuropsychologist Catalina Pons Marquès, Health Psychologist, Master's in Neuropsychology.
- Dr. María Giné.
- Researchers from the Neurodegenerative Diseases Group at VHIR (Vall d'Hebron Research Institute).

# 12. Difusion plan, communication, oral presentations and prizes,

- Social Media: We have created an account on Instagram, and currently, we have more than 3,300 followers
- We have also created a 60-second video outlining the main objectives of the research, showcasing the team and the key institutions involved in this international collaboration between Spain and Portugal (Blanquerna-URL, CNC-Portugal).

The results obtained in this project will be disseminated through complete publications in high-impact original peer-reviewed scientific journals (e.g., Metabolism: Clinical and Experimental, IF = 6.51, Top 6%, and Journal of Clinical Investigation). All publications will follow an open-access format. The findings will be presented in the form of oral presentations and posters at various national and international conferences such as the European Society for Clinical Investigation.

Training: This project will contribute to human resources training, potentially resulting in several master's and doctoral theses. I also intend to organize scientific awareness activities focused on demonstrating that exercise intervention can improve the phenotype of Parkinson's disease (PD) and slow down its progression.

Communication: This field is a new and powerful area of action in life sciences, and my goal is to contribute to reducing the harmful effects of PD. I believe that the project contributes to the UN's goals by advising on lifestyle interventions that can correct and/or prevent disease progression to ensure a healthy life and promote well-being across all ages.

Supported by CNC's extensive experience in scientific communication, we will participate in scientific activities targeted at different audiences and interest groups. This includes open lab days, activities in scientific centers and museums, school activities for different target groups, media coverage (radio, television, newspapers), regular updates on social media (including LinkedIn and Facebook), production of printed materials, including brochures, and engagement with specialized health professional groups.

We believe that the project aligns perfectly with the mission of the host institution, resulting in a significant contribution to critical social challenges. The results obtained in this study will be disseminated by presenting them to scientific journals, conferences, and events related to physical activity, neurodegenerative diseases, Parkinson's disease, health sciences, biomedicine, neurology, mitochondrial health and exercise, sports and physical activity sciences, neuroscience, among others.

Presentation of Abstract at the CINPSUS-I Congress (International Interdisciplinary Congress on Public Health Policies).

| **Authors** | **Presenting author** | **Title** | **Congress** | **Organizer and dates** | **Quality** |
| --- | --- | --- | --- | --- | --- |
| **-Juan Carlos** | Juan Carlos | Abstract: EVALUATING THE | CINPSUS - I | Done by the Grupo | Code of the |
| **Magaña [1]** | Magaña | EFFICACY OF TWO PHYSICAL | International | de Investigación | abstract |
| **-Catalina Pons** |  | ACTIVITY PROGRAMS TO | Interdisciplin | Interdisciplinar en | Approved |
| **Marqués [2]** |  | PREVENT PARKINSON´S | ary Congress | Salud, Educación y | By the |
| **-Maria Giné-** |  | DISEASE PROGRESSION | on Public | Educación Física- | Scientific |
| **Garriga [3]** |  | USING MITOCHONDRIAL | Health | GIPEEF-Universidad | Commission |
| **-Susana P.** |  | FUNCTION FROM PATIENTS | Policies | Federal del Valle de San | Of the |
| **Pereira [4]** |  | SKIN FIBROBLASTS AS |  | Francisco | Meeting: |
| **-Joel Montané** |  | BIOMARKER |  | 18/06/2021 | 00535 |
| **[5]** |  |  |  | 19/06/2021 |  |

The CINPSUS congress was truly a great success, with more than 3,100 applicants from 14 different nationalities, and over 1,800 submissions in Portuguese, Spanish, and English. In the end, 392 papers were presented, and the best 30 papers were awarded. Ours was one of the top 30 papers.

We also plan to disseminate, attend, and/or participate in conferences such as the European Society for Clinical Investigation meeting, National Congress of Physical Activity and Sports Sciences (CAFE), National Congress of Health Sciences Students, CAFE Congress, European Congress on Neurodegenerative Diseases, Parkinson's Congress organized by the Spanish Parkinson's Federation, International Neuropsychological Society World Congress, International Congress on Non-Motor Dysfunctions in Parkinson’s Disease and Related Disorders.

12.1 Journals:

- Different Q1 journals will be considered based on the results.
- Physiotherapy, Physical Therapy, Journal of Physiotherapy, Disability and Rehabilitation, International Journal of Neuroscience, Physical Education Journals, among others.

# 13. References

[1] OMS | Los trastornos neurológicos afectan a millones de personas en todo el mundo: informe de la OMS [Internet]. Disponible en: <http://www.who.int/mediacentre/news/releases/2007/pr04/es>

[2] OMS | ¿Qué son los trastornos neurológicos? [Internet]. Disponible en:<https://www.who.int/features/qa/55/es/>

[3] Sánchez, C. S. (2006). Impacto Sociosanitario De Las Enfermedades Neurológicas En España. Fundación Española de Enfermedades Neurológicas (FEEN). Retrieved from<http://www.fundaciondelcerebro.es/docs/imp_sociosanitario_enf_neuro_es.pdf>

[4] Domingo, E. P., Sierra, M. G., Valero, M. M., & Castiñeira, M. P.-O. (2015). El Libro Blanco del párkinson en España - Aproximación, análisis y propuesta de futuro. Madrid: Real Patronato sobre Discapacidad (Ministerio de Sanidad, Servicios Sociales e Igualdad) y Federación Española de Párkinson.

Retrieved from <http://www.fedesparkinson.org/libro_blanco.pdf>

[5] Garcés, Mario y Crespo Puras, María del Carmen y Finkel Morgenstern, Lucila y Arroyo Menéndez, Millán (2016) Estudio sobre las enfermedades neurodegenerativas en España y su impacto económico y social.

[6] Erbach, G. (2013). Neurodegenerative diseases in the workplace. Library of the European Parliament. [7] Tu PH, Galvin JE, Baba M, et al. Glial cytoplasmic inclusions in white matter oligodendrocytes of multiple system atrophy brains contain insoluble alpha-synuclein. Ann Neurol 1998;44:415-422.

[8] Martínez-Fernández., R., Gasca-Salas C., C., Sánchez-Ferro, Á., & Ángel Obeso, J. (2016). ACTUALIZACIÓN EN LA ENFERMEDAD DE PARKINSON. Revista Médica Clínica Las Condes, 27(3), 363–379. https://doi.org/https://doi.org/10.1016/j.rmclc.2016.06.010

[9] Carvalho, A., Barbirato, D., Araujo, N., Martins, J. V., Cavalcanti, J. L., Santos, T. M., Coutinho, E. S., Laks, J., & Deslandes, A. C. (2015). Comparison of strength training, aerobic training, and additional physical therapy as supplementary treatments for Parkinson's disease: pilot study. Clinical interventions in aging, 10, 183–[191. https://doi.org/10.2147/CIA.S68779](https://doi.org/10.2147/CIA.S68779)

[10] Ambrosi, G., Ghezzi, C., Sepe, S., Milanese, C., Payan-Gomez, C., Bombardieri, C. R., Armentero, M. T., Zangaglia, R., Pacchetti, C., Mastroberardino, P. G., & Blandini, F. (2014). Bioenergetic and proteolytic defects in fibroblasts from patients with sporadic Parkinson’s disease. Biochimica et Biophysica Acta -

Molecular Basis of Disease, 1842(9). <https://doi.org/10.1016/j.bbadis.2014.05.008>

[11] Deus, C. M., Pereira, S. P., Cunha-Oliveira, T., Pereira, F. B., Raimundo, N., & Oliveira, P. J. (2020). Mitochondrial remodeling in human skin fibroblasts from sporadic male Parkinson’s disease patients uncovers metabolic and mitochondrial bioenergetic defects. Biochimica et Biophysica Acta - Molecular Basis of Disease, 1866(3). <https://doi.org/10.1016/j.bbadis.2019.165615>

[12] Brooks, G. A., Brown, M. A., Butz, C. E., Sicurello, J. P., & Dubouchaud, H. (1999). Cardiac and skeletal muscle mitochondria have a monocarboxylate transporter MCT1. Journal of applied physiology (Bethesda, Md. : 1985), 87(5), 1713–1718. <https://doi.org/10.1152/jappl.1999.87.5.1713>

[13] Acin-Perez, R., Benador, I. Y., Petcherski, A., Veliova, M., Benavides, G. A., Lagarrigue, S., Caudal, A., Vergnes, L., Murphy, A. N., Karamanlidis, G., Tian, R., Reue, K., Wanagat, J., Sacks, H., Amati, F.,

Darley-Usmar, V. M., Liesa, M., Divakaruni, A. S., Stiles, L., & Shirihai, O. S. (2020). A novel approach to measure mitochondrial respiration in frozen biological samples. The EMBO Journal, 39(13), e104073. https://doi.org/https://doi.org/10.15252/embj.2019104073

[14] Carter, H. N., Chen, C. C., & Hood, D. A. (2015). Mitochondria, muscle health, and exercise with advancing age. Physiology (Bethesda, Md.), 30(3), 208–[223. https://doi.org/10.1152/physiol.00039.2014](https://doi.org/10.1152/physiol.00039.2014) [15] Sen C. K. (1995). Oxidants and antioxidants in exercise. Journal of applied physiology (Bethesda, Md. : 1985), 79(3), 675–686. <https://doi.org/10.1152/jappl.1995.79.3.675>

[16] Hoppeler, H., Howald, H., Conley, K., Lindstedt, S. L., Claassen, H., Vock, P., & Weibel, E. R. (1985). Endurance training in humans: aerobic capacity and structure of skeletal muscle. Journal of applied physiology (Bethesda, Md. : 1985), 59(2), 320–327. <https://doi.org/10.1152/jappl.1985.59.2.320>

[17] Barrios, L., & López, M. (2011). Aportes del ejercicio físico a la actividad cerebral. Lecturas: Educación Física y Deportes (Revista Digital) (160), N/A.

[18] La actividad física como factor benéfico a nivel neurológico Vol. 3, núm. 1., (2019) Juan Antonio Vera Hinojosa; Karla Lissette Flores Flores; Natalia del Carmen Alvarado, Linda Beatriz Dávila Solórzano.

[19] Severinsen, M., & Pedersen, B. K. (2020). Muscle-Organ Crosstalk: The Emerging Roles of Myokines.

Endocrine reviews, 41(4), 594–609. <https://doi.org/10.1210/endrev/bnaa016>

[20] Soria, Bernat. «Biomedical research in Spain: the patient’s point of view». Contributions to science, [en línia], 2009, p. 91-94, https[://www](http://www.raco.cat/index.php/Contributions/article/view/188419).[raco.cat/index.php/Contributions/article/view/188419](http://www.raco.cat/index.php/Contributions/article/view/188419) [Consulta: 29-04-2021].

[21] Bloomer, R. J., Schilling, B. K., Karlage, R. E., Ledoux, M. S., Pfeiffer, R. F., & Callegari, J. (2008). Effect of resistance training on blood oxidative stress in Parkinson disease. Medicine and Science in Sports and Exercise, 40(8), 1385–1389.

[22] G. Auburger, M. Klinkenberg, J. Drost, K. Marcus, B.Morales-Gordo, W.S. Kunz, et al., Primary skin fibroblasts as amodel of Parkinson's disease,Mol. Neurobiol. 46 (2012) 20–27.

[23] C. Mytilineou, P.Werner, S. Molinari, A. Di Rocco, G. Cohen, M.D. Yahr, Impaired oxidative decarboxylation of pyruvate in fibroblasts from patients with Parkinson's disease, J. Neural Transm. Park. Dis. Dement. Sect. 8 (1994) 223–228.

[24] Milanese, C., Payán-Gómez, C., Galvani, M., Molano González, N., Tresini, M., Nait Abdellah, S., van Roon-Mom, W. M. C., Figini, S., Marinus, J., van Hilten, J. J., & Mastroberardino, P. G. (2019). Peripheral mitochondrial function correlates with clinical severity in idiopathic Parkinson’s disease. Movement Disorders : Official Journal of the Movement Disorder Society, 34(8), 1192–1202.<https://doi.org/10.1002/mds.27723>

[25] Miyai, I., Fujimoto, Y., Ueda, Y., Yamamoto, H., Nozaki, S., Saito, T., & Kang, J. (2000). Treadmill training with body weight support: its effect on Parkinson's disease. Archives of physical medicine and rehabilitation, 81(7), 849–852. <https://doi.org/10.1053/apmr.2000.4439>

[26] Steffen, T., Petersen, C., & Dvorak, L. (2012). Community-based exercise and wellness program for people diagnosed with Parkinson disease: experiences from a 10-month trial. Journal of geriatric physical therapy (2001), 35(4), 173–180. <https://doi.org/10.1519/JPT.0b013e31824a1c9d>

[27] Gobbi, L. T., Oliveira-Ferreira, M. D., Caetano, M. J., Lirani-Silva, E., Barbieri, F. A., Stella, F., & Gobbi, S. (2009). Exercise programs improve mobility and balance in

people with Parkinson's disease. Parkinsonism & related disorders, 15 Suppl 3, S49–S52.<https://doi.org/10.1016/S1353-8020(09)70780-1>

[28] Tanaka, K., Quadros, A. C., Jr, Santos, R. F., Stella, F., Gobbi, L. T., & Gobbi, S. (2009). Benefits of physical exercise on executive functions in older people with Parkinson's disease. Brain and cognition, 69(2), 435–[441. https://doi.org/10.1016/j.bandc.2008.09.008](https://doi.org/10.1016/j.bandc.2008.09.008)

[29] Hoehn, M., & Yahr, M. (2001). Hoehn MM, Yahr MD. Parkinsonism: onset, progression and mortality.

Neurology 17: 427-442. Neurology, 57, S11-26. <https://doi.org/10.1212/WNL.17.5.427>

[30] Conde, V., Esteban, T. y Useros, E. (1976). Revisión crítica de la adaptación castellana del Cuestionario de Beck. Revista de Psicología General y Aplicada, 31, 469-497.

[31] Conde, V., Escriba, P. e Izquierdo, J.A. (1970). Evaluación estadística y adaptación castellana de la Escala Autoaplicada para la Depresión (SDS) de Zung. Publicaciones de la Sociedad Española de Psicología, 30, 867-880.

[32] Conde, V., y Useros, E. (1974). El inventario para la medida de la depresión de Beck. Revista de Psiquiatría y Psicología Médica de Europa y América Latina, 12, 153-167

***Referencias de Anexos (En el documento “Principios básicos de la Ética aplicada a la Investigación”):*** [33] Speelman, A. D., van de Warrenburg, B. P., van Nimwegen, M., Petzinger, G. M., Munneke, M., & Bloem, B. R. (2011). How might physical activity benefit patients with Parkinson disease?. Nature reviews.

Neurology, 7(9), 528–534. https://doi.org/10.1038/nrneurol.2011.107

[34] Cruise, K.E., Bucks, R.S., Loftus, A.M., Newton, R.U., Pegoraro, R., Thomas, M.G. (2010). Exercise and Parkinson’s: benefits for cognition and quality of life. Acta Neurologica Scandinavica, 123, 13-19. [35] Crizzle, A. M., & Newhouse, I. J. (2006). Is physical exercise beneficial for Persons with Parkinson’s disease. Clinical Journal of Sport Medicine, 16, 422–425.

[36] Tolosa, E., Wenning, G., & Poewe, W. (2006). The diagnosis of Parkinson's disease. The Lancet.

Neurology, 5(1), 75–86. https://doi.org/10.1016/S1474-4422(05)70285-4

[37] Wieckowski MR, et al. Recovering Mitochondrial Function in Patients’ Fibroblasts. In: Mitochondrial Biology and Experimental Therapeutics (ed^(eds) (2018).

[38] Goetz CG. [Movement Disorder Society-Unified Parkinson’s Disease Rating Scale (MDS-UPDRS): a new scale for the evaluation of Parkinson’s disease]. Rev Neurol (Paris) 2010;166:1-4.

# 14. Annexes

- Basic Principles of Applied Ethics in Research
- Informed consent General-URL (in Spanish, original language)
- Patient information sheet (in Spanish, original language)
- General Data Protection Regulation (GDPR)

***Principios básicos de la Ética aplicada a la Investigación***

**INDICE:**

##### Fundamentación y Justificación de la investigación.

**- “La triada de un abordaje Dual, Físico-Coordinativo y Funcional, con un enfoque terapéutico”.**

1. **Consideraciones Éticas de esta investigación.**
2. **Apartado descripción del grupo vulnerable.**
3. **Apartado Consentimiento Informado e Información del paciente.**
4. **Apartado Principios básicos de la Ética aplicada a la Investigación. Tradición Anglo-Americana. “Un recorrido por los principios éticos de la investigación”**

**(Principio de no Maleficencia-Principio de Beneficencia-Principio de Autonomía-Principio de Justicia)**

1. **Apartado de Reflexión Ética sobre la investigación que realizaré.**
2. **Fundamentación y Justificación de la investigación.**

Creemos que es de vital importancia para el desarrollo de los principios éticos de la investigación, el correcto entendimiento del qué, porqué, para qué y el cómo, desarrollado en el Plan de Recerca (PR). También en el PR hemos citado estudios previos respecto a la enfermedad de Parkinson (EP) y la importancia de analizar los fibroblastos, para valorar su función mitocondrial. A esos estudios previos proponemos e incluimos, la dimensión de la intervención de la actividad física (AF). Hasta el día de hoy aún no se ha llevado a cabo, este tipo de investigación con intervención por parte de las Ciencias de la Actividad Física.

Al día de hoy es numerosa la evidencia de los beneficios de la AF en la EP. Centrándonos en nuestra investigación, tenemos como objetivo el exponer la relación entre la AF y la fisiopatología de la EP; y cómo afecta a los mecanismos de la función mitocondrial y su remodelación.

Los beneficios generales del ejercicio, también se espera en los pacientes con EP que realizan AF [33].

Hemos citados dos estudios que mostraron la implicancia de un programa de ejercicio aeróbico en el deterioro cognitivo en pacientes con EP, y también constataron efectos positivos en la función ejecutiva [29, 34]. Dentro de las pérdidas de funciones y deterioro, numerosas habilidades motrices se ven comprometidas, entre estas encontramos la postura, el equilibrio, la marcha y las transferencias. La AF ayuda a mejorar este rendimiento funcional [35]. Esta es una de las razones por las que hemos planteado en uno de los programas de AF, el abordaje Dual, Físico-Coordinativo y Funcional. Aunque en ambos programas de AF tenemos como objetivo adentrarnos en el estudio de la función mitocondrial, para una mejor comprensión de los efectos beneficiosos, de la AF como abordaje terapéutico.

Por esta razón es imprescindible el realizar una extracción de células de la piel, para analizar los fibroblastos de los pacientes con EP. El análisis de los fibroblastos de piel nos ayudará a valorar la función mitocondrial de los pacientes; y también como la AF influye en los déficits proteolíticos y bioenergéticos celulares, asociados a la enfermedad.

En las conclusiones de Bloomer [21] vislumbramos una llamada a investigar en esta área, ya que se sorprenden de que ningún estudio hasta la fecha ha investigado el papel del ejercicio estructurado, para mejorar el estado oxidativo en personas con EP. Bloomer se centró en estudiar a nivel sanguíneo los efectos del entrenamiento de resistencia sobre el estrés oxidativo en la EP.

Hasta el momento no se han encontrado estudios que relacionen la clínica de la EP, que utilicen como biomarcadores a las organelas celulares (por medio de los fibroblastos) analizando el impacto de distintos tipos de AF, y cómo influyen en los déficits de la bioenergética celular. Esta es la primera investigación que estudiará los efectos de la AF, en la disfunción mitocondrial, utilizando el modelo de estudio de los fibroblastos de piel en personas con EP.

El estudio del sistema nervioso humano, posee la complejidad y dificultad debido a la inaccesibilidad del tejido, sumado a las limitaciones de los modelos celulares y animales de reproducir su fisiología. En lo que respecta a las investigaciones de mecanismos moleculares relacionadas con el envejecimiento y con las EN, hace años que se encuentran utilizando animales modificados genéticamente, pero que no terminan de reproducir del todo las patologías, ni los fenotipos asociados a la vejez.

Citando parte de una de las conclusiones del estudio de Ambrosi [10] “la investigación de los mecanismos patogénicos en las células periféricas, como los fibroblastos derivados de pacientes con EP esporádica (EPs) y controles emparejados por edad/sexo, podría generar una comprensión más profunda de los déficits que afectan a las neuronas dopaminérgicas, y posiblemente, nuevas herramientas aplicables a la práctica clínica.” Es así que, en medio de esta conclusión, nuestra investigación pretende incorporar/aportar una nueva variable, que será analizada por medio de los fibroblastos; para obtener nuevas conclusiones/resultados. Y poder aportar evidencia a nivel celular y molecular de cómo influye los distintos programas de AF en pacientes con EP.

##### “La triada de un abordaje Dual, Físico-Coordinativo y Funcional, con un enfoque terapéutico”.

La triada de un abordaje dual, físico-coordinativo y funcional, con un enfoque terapéutico, en donde la AF (planteada en 2 programas distintos) ejerce de hilo-conductor (y motor principal) de la biología celular (por un lado) y sus valores fisiológicos; enlazando a su vez con la fisiopatología de la EP (por el otro extremo de la “triada”) focalizada en la disfunción mitocondrial y estrés oxidativo. Para evaluar y analizar la clínica propia del paciente en cada etapa de la investigación, en lo que respecta a sus síntomas motores y no motores, psicológicos y de calidad de vida. Y de esta manera poder correlacionar la influencia, eficacia y eficiencia de los distintos programas de AF (propuestos por la investigación) en relación a los fibroblastos de piel de pacientes con EP, que ejercerán como biomarcador encargados de valorar la función mitocondrial y el estrés oxidativo. Siendo los Fibroblastos una valiosa alternativa a lo que sería una muestra de tejido cerebral, que por obvias razones de riesgos-beneficios estaría totalmente contraindicado.

Es por esto que proponemos un camino mínimamente invasivo, con una técnica conocida y ya implantada en diversos estudios. Dichos estudios, ya han arrojado resultados preliminares (citado en el plan de recerca), y el camino de esos estudios ha sido primero con animales y luego con humanos; y también han aportado luz a otras EN como el Alzheimer.

Creemos que es fundamental, hacer referencia y dejar constancia, que esta investigación cuenta con el apoyo del Center for Neuroscience and Cell Biology at the University of Coimbra (CNC-UC), Portugal. Una de las investigadoras principales del CNC-UC (Dra. Susana P. Pereira) es autora de los estudios citados en el PR (con resultados preliminares), y es Co-directora de esta tesis. Sumado a la Co-dirección del Dr. Joel Montané de la FCS-Universidad Blanquerna, cuyo apoyo y asesoramiento especializado en el complejo “universo” mitocondrial, hacen que esta investigación pueda adentrarse en la fisiopatología de la EP. Para investigar cómo responde la disfunción mitocondrial propia de la EP, teniendo en cuenta la clínica por la que transcurre el paciente; y poder así relacionar con su valoración motora (MDS-UPDRS III) y demás valoraciones, antes y después de la intervención de AF especialmente diseñada. Y también con la colaboración del Grupo de Enfermedades Neurodegenerativas del Hospital Universitario-Institut de Recerca Vall d’Hebron (VHIR), Barcelona. Mediante la supervisión del Dr. Jorge Hernández Vara (Neurólogo), con la colaboración de la neuróloga Daniela Samaniego Toro, y las investigadoras Dra. Marta Martínez-Vicente y Dra. Ariadna Laguna Tuset.

##### Consideraciones Éticas:

Esta investigación se plantea teniendo en cuenta los principios éticos de la tradición Americana y la legislación nacional e internacional aplicable en esta materia, así como la Carta de los Derechos Fundamentales de la Unión Europea y el Convenio Europeo de Derechos Humanos. Nos hemos basado en la tradición Anglo-Americana de los Principios Éticos de la Investigación. En todo momento de la investigación, se mantendrá presente la intimidad y privacidad física de las personas, como así también la confidencialidad de los datos solicitados y establecidos propios de la investigación. La investigación que hemos planteado siguen las normas que hacen referencia al Reglamento General Europeo de Protección de Datos (RGPD), Reglamento (UE) 2016/679; y privacidad y confidencialidad de datos (LOPDGD) Ley Orgánica (3/2018 de 5 de diciembre) de Protección de Datos Personales y garantía de los derechos Digitales. Los procedimientos y medidas establecidas para cumplir la privacidad y confidencialidad, se ha explicado en el documento de Información del paciente.

##### Apartado descripción del grupo vulnerable:

Las personas mayores son un grupo mayoritario en Europa, en los próximos años se dispararán las estadísticas de este colectivo y por ende las enfermedades verán como sus prevalencias aumentan.

Como ya hemos dicho en el plan de recerca, las EN se encuentran dentro de los trastornos neurológicos, entre otras enfermedades (OMS) [2]. Las EN tienen una gran implicación en el sistema sanitario y en la sociedad. “Al propio proceso de la enfermedad, hay que sumar el impacto psíquico, la mengua en la calidad de vida, la incapacidad laboral, la pérdida de habilidades sociales, el gravamen de los cuidadores y las situaciones de dependencia” [4]. En España casi 1 millón de personas padecen una EN, donde el 40% de los afectados dejan de trabajar por la enfermedad, y el 53% tienen dificultad económica a causa de la misma (esto sin tener en cuenta que a día de hoy habría que sumar los efectos del COVID-19) [5]. La EP es la segunda EN más prevalente en la actualidad, después del Alzhéimer, y pertenece a los llamados Trastornos del Movimiento [6].

El Grupo Vulnerable a investigar en este Plan de Recerca, son pacientes con EP. Este grupo vulnerable ronda la cifra de 1 millón en Europa, y en España se estiman unas 160.000 personas. Con la dificultad agregada que posee su diagnóstico, esta cifra puede variar hasta casi 300.000 personas [5]. Y no menos importante, existe un 30% de pacientes con EP de inicio temprano aún no diagnosticados, y que no se han añadido a las cifras de la prevalencia. Siendo la dificultad del diagnóstico [36], uno de los motivos claves por los cuales gran parte de las investigaciones de hoy en día, pretenden identificar un biomarcador para optimizar su diagnóstico. En el marco de nuestra investigación, utilizaremos a los fibroblastos de piel como biomarcador, para la valoración de la función mitocondrial, en pacientes con EP que realizan distintos programas de AF.

Actualmente en las sociedades de la mayoría de los países de Europa, muestran un factor común respecto a la tasa de natalidad y la esperanza de vida (menor la primera y mayor la segunda); dando como consecuencia un envejecimiento progresivo de la población. Es este factor el que dispara una curva exponencial, respecto a la evolución de las EN en el mundo, duplicándose de aquí a 20 años, y se triplicará de aquí a 30 años (2050).

##### Apartado Consentimiento Informado e Información del paciente.

Se han elaborado los siguientes Consentimientos informado:

-C.I. General-URL -Hoja de Información del paciente (HIP)

-Permisos Centros

-C.I. Específico (Muestras biológicas para la investigación biomédica, y muestras biológicas sobrantes)

-En el documento de “(HIP)” se explica cómo se anonimizarán los datos, para preservar la intimidad de la persona. También hemos dejado constancia (y en el documento de información del paciente) de como los participantes de esta investigación verán reconocido sus derechos.

-La información sobre las Biopsias de los Fibroblastos de piel se encuentra al final de la HIP.

##### Apartado Principios básicos de la Ética aplicada a la Investigación. Tradición Anglo-Americana: “Un recorrido por los principios éticos de la investigación”

**(Principio de no Maleficencia-Principio de Beneficencia-Principio de Autonomía-Principio de Justicia)** Este apartado lo abordaremos realizando un recorrido por los principios éticos de la investigación, en relación a los aspectos más relevantes de este estudio.

Uno de los aspectos claves respecto a los Principios ético de esta investigación, lo hemos abordado desde el inicio del *Diseño* y el planteamiento de los *Objetivos* de esta investigación; y en base a la *fundamentación* y el *estado actual de la cuestión*. Nos referimos al uso de los fibroblastos de piel, que trae asociado la extracción de biopsia de piel, lo cual es irremplazable mediante otras técnicas. En este tipo de biopsia de piel (descriptas en la HIP), las molestias y los riesgos son mínimos; y a esto sumamos la experiencia de la Dra. Marta Martínez-Vicente en otros estudios de investigación, en los cuales también se han obtenido biopsias de piel de pacientes. Wieckowski_2018 afirma que *“los fibroblastos humanos (por Ej., de una biopsia de piel) son una fuente valiosa y confiable de material biológico para el estudio de una amplia gama de enfermedades, y es un procedimiento mucho menos invasivo en comparación con las biopsias de músculo o hígado.”* [37].

En un análisis profundo *del estado de la cuestión*, y bajo la supervisión de los directores de Tesis especializados en el Área de la Biología Celular (disfunción mitocondrial, estrés oxidativo) y Biomedicina, y con el respaldo de una de las investigadoras principales del CNC (Susana P. Pereira); hemos analizado estudios que afirman, que *“los fibroblastos de piel humana se han convertido en un camino muy acertado para estudiar diferentes patologías y trastornos neurodegenerativos como el Parkinson y la enfermedad de Alzheimer”* [10] (Ambrosi et al. , 2014 ; Cameron et al. , 2004 ; Hu et al. , 2015 ), “*debido a su metabolismo, disponibilidad y robustez fisiológicamente relevantes; y a su localización ya que pueden aislarse de los pacientes mediante métodos menos invasivos”* [22] en comparación a lo que sería aislar un tejido nervioso. Hay estudios que solo obtienen la información post mortem, debido a la inaccesibilidad del tejido.

Y es en los fibroblastos donde encontramos las mismas disfunciones que suceden en un tejido neuronal. Deus et al., 2020 detectaron alteraciones metabólicas y mitocondriales que también existirían en un tipo de célula no neuronal [11]. Por tanto, este estudio de Deus et al.,2020, es clave para nuestra investigación, ya que nos permite adentrarnos en uno de los aspectos de la fisiopatología de la EP, para evaluar la disfunción mitocondrial y que efectos produce un programa de AF.

También recomienda Auburger que *“las células primarias pueden tener varias ventajas para evaluar la toxicidad inducida por mitocondrias, incluido el metabolismo relevante, la accesibilidad humana y la traducción clínica”.* Sabemos por el *Marco Teórico,* que los Fibroblastos son protagonistas tanto en el mantenimiento, como en la reparación de los tejidos. Lo que hace que los Fibroblastos sea un biomarcador adecuado para el estudio de las EN según Auburger, es la capacidad de “*reflejar el daño celular acumulativo y las mutaciones”* [22].

Habiendo abordado uno de los aspectos claves de esta investigación, continuamos con otros aspectos también relevantes, de todo aquello que se tendrá en cuenta, para evitar causar un mal en primer lugar, y poder buscar un bien, antes, durante y después de la intervención.

##### Antes de la intervención:

-La correcta información del paciente, consentimiento informado, y explicación personal e individualizada.

-El análisis individualizado de la clínica que presenta el paciente en ese momento, la cual se realizará mediante una anamnesis (historia clínica detallada) para valorar la trayectoria y evolución que ha tenido su enfermedad.

-Se realizarán unos Test iniciales, no solo por el objetivo que tiene la investigación en cuanto analizar la intervención, sino también para tener en cuenta una posible modificación y adaptación de los parámetros de la AF a desarrollar, tales como carga, volumen, intensidad, repeticiones, frecuencia, recuperación, entre otras variables.

##### Durante la intervención:

-Cada una de las actividades programadas se llevarán a cabo habiendo realizado su correspondiente entrada en calor (general y específica) en función de los objetivos de la sesión.

-También se llevará a cabo al final de cada sesión, la “vuelta a la calma”, con el fin de recuperar los valores fisiológicos.

-En cada sesión se dejará un tiempo “Pre-sesión” y “Post-sesión”. Los cuales estarán destinados a valorar como llega el paciente, como se ha sentido desde la pasada sesión, se resolverán dudas y también se recogerá el feedback, una vez finalizada la sesión. Lo cual será clave para una correcta retroalimentación de lo planificado. **Después de la intervención:**

-Se seguirán realizando los controles pertinentes de la clínica, se les motivará y orientará para que mantengan unos niveles de actividad física aconsejables.

-Habrá un acompañamiento terapéutico durante todas las fases de la intervención (antes-durante y después).

Aunque ya hemos ido exponiendo la fundamentación y su justificación en este documento, ahora lo hacemos respecto de una de las dimensiones “del mal” (mal físico) del Principio de Maleficencia.

Somos muy conscientes de que un aspecto clave para esta investigación es el análisis de los fibroblastos de piel. Y en el mismo sentido es también clave desde el punto de vista de los principios básicos de la Ética aplicada a la Investigación. La recolección de las muestras de fibroblastos de piel, aunque técnicamente esté bajo el nombre de biopsia, es una de las menos invasivas, aunque no por esta razón somos menos conscientes de lo que significa para el paciente. Cada una de las etapas de esta investigación han sido “pesadas” en la “balanza riesgo- beneficios”; para asegurarnos de que sean mayores los beneficios a conseguir.

En el apartado de *discusiones* de los estudios de C. Deus y S. P. Pereira (2020) [11], nos afirma que *“la medición de la respuesta mitocondrial individual, a los agentes químicos en los fibroblastos de la piel, puede generar ahorros significativos en el deterioro de la salud del paciente.”*

Es casi inviable que un paciente por su propia cuenta, pueda solicitar un estudio de esta naturaleza. Y por esto creemos que es un aporte (contraprestación) a los pacientes que participen en la investigación.

Los resultados hallados no solo servirán de cara a la investigación y a la sociedad, y al resto de personas con Parkinson en España, Europa, y en todo el mundo. Con dichos resultados podremos optimizar con cada participante (paciente) de la investigación, cuantificándole su plan de AF lo más personalizado posible. Por medio del análisis de la muestra extraída de Fibroblastos, podremos evidenciar el estado actual de su enfermedad a nivel celular. Y así, obtendrá un examen detallado de los parámetros relacionados con su salud, su función física, y su función cognitiva. Al final de la intervención le entregaremos dicho plan de entrenamiento personalizado, junto con un informe final de los resultados y pruebas realizadas (Médicas, biológicas y físicas).

Esta será una de las contraprestaciones más valiosas que podrán beneficiarse los participantes de esta investigación.

Somos conscientes de que el llevar a cabo la actividad física programada, más otros tipos de test, pruebas biológicas y demás; todas ellas llevan consigo un tiempo de dedicación por parte de los sujetos investigados. Pero también somos conscientes que en el otro extremo de la balanza se encuentran los beneficios de los efectos de la actividad física programada y todo lo que puede aportar esta investigación a cada sujeto en particular.

También tenemos en cuenta que con la intervención estamos separando a los pacientes de su entorno habitual (3 veces por semana) para asignarlo en un grupo, entendemos que esto puede llegar a generar algún tipo de estrés inicial. Pero también sabemos del potencial social de la actividad física, y el potencial psicológico (aportando para disminuir las dimensiones del “mal social y mal psicológico”) de poder compartir experiencias con otras personas, que también comparten diagnóstico y el vínculo social de la actividad física compartida.

Estaríamos reforzando uno de los principios fundamentales para mantener una salud mental, la sociabilización. Por fortuna la actividad física, posee la dimensión y capacidad de poder tener efectos holísticos e integradores, y que el beneficio sea en todas las áreas.

Uno de los objetivos presentes en la búsqueda de la muestra (N), es que todos los tipos de intervenciones se puedan llevar a cabo en el mismo sitio, para evitar desplazamientos a los sujetos investigados.

Así como el evitar causar un mal es un deber, el causar un bien también los es. Es así que varias de las razones que hemos citado con el fin de evitar causar un mal, también son citables en este apartado, porque en sí mismo forman parte de los beneficios que se pretenden conseguir.

En esta investigación no nos conformamos con desearlo y solo citar referencias y bibliografía, lo cual estaría bastante bien, pero el diseño que hemos creado se basa en la intervención. Lo cual entraña toda una complejidad, y todo un diseño en el que tenemos el deber y toda la responsabilidad de llevarlo hacia su máxima expresión.

Procedemos a citar los efectos beneficiosos de la AF, sobre el organismo de una persona con EP. El mismo Speelman et al. (2011) [33] lo sintetiza en un decálogo de razones por las cuales el ejercicio físico puede beneficiar a pacientes con EP, y éstas son: “prevenir complicaciones cardiovasculares, detener la osteoporosis, mejorar la función cognitiva, prevenir la depresión, mejorar el sueño, disminuir el estreñimiento, disminuir la fatiga, mejorar el rendimiento funcional, mejorar la eficacia de la medicación y optimizar el sistema dopaminérgico”.

Esta interacción en distintas áreas del conocimiento como son la biología celular, las ciencias de la actividad física y la enfermedad de Parkinson traerá consigo beneficios a la sociedad, los cuales hemos citados en el plan de recerca, y el mayor de los beneficios que aspiramos, es el poder mejorar la calidad de vida de las personas con EP e indirectamente la de sus familiares.

Unos de los criterios de inclusión será que la cognición esté preservada, no solo por el diseño y los objetivos propios de la investigación, también tiene repercusión en el correcto entendimiento y aceptación del consentimiento informado; lo cual se traduce en una garantía del principio de Autonomía, reforzado por el binomio *información-comprensión.*

También se tendrá en cuenta en la evaluación inicial (dentro de la anamnesis) si el sujeto vive solo, acompañado, y/o con sus hijos, para valorar la total autonomía y confirmar la comprensión del C.I.

La forma en que se anonimizarán los datos, será mediante un Código de Identificación del Sujeto. Es un Identificador único que asigna el investigador a cada sujeto del ensayo para proteger su identidad, y que se utiliza en vez del nombre del sujeto.

También en el documento de “Información del Paciente” hemos dejado constancia de como los participantes de esta investigación verán reconocido sus derechos.

En lo personal, esta tesis me permite volcar los conocimientos adquiridos durante varios años, desde distintas especialidades (siempre sobre los pilares de la Salud y la Educación), actualizarlos, y relacionarlo con nuevos conocimientos y nuevas áreas de especialización. Y de esta forma poder canalizar el foco, en los pacientes con EP.

Este estudio será un gran aporte al grupo SAFE y a la Universidad Blanquerna-URL, la cual me ha brindado la posibilidad de un contrato Pre-doctoral de Personal Investigador en Formación.

Sin duda alguna, el conocimiento obtenido en este proyecto también será de gran aporte al Center for Neurosciencie and cell Biology-University of Coimbra (Portugal), como así también al grupo de enfermedades Neurodegenerativas del Hospital Universitario VHIR.

También es un bien para mi persona, una REALIZACIÓN…un “SERVIR PARA SERVIR”.

##### 7. Apartado de Reflexión Ética sobre la investigación que realizaré

Esta investigación cuenta con el apoyo del contrato pre-doctoral para personal investigador en formación- Blanquerna (PIF), dentro del grupo SAFE. El motor principal es la Actividad Física; y si bien, el nivel de concreción está dentro de las EN, concretamente la EP, se podría vislumbrar que este tipo de intervenciones podría sentar precedente para el resto de las EN, ya que todas ellas comparten la disfunción mitocondrial. Esta investigación está diseñada para los pacientes con EP, pero la esencia protocolar de los fibroblastos de piel (desde el diseño de grupos para controles por edad y sexo, hasta la extracción y posteriores fases) también es útil para otras EN. Por lo tanto, una parte de esta investigación trasciende las fronteras de la EP, expandiéndose hacia las EN, con lo cual sin ser este nuestro objetivo principal, no deja de ser un valor a tener en cuenta desde una dimensión científica, innovadora y social. Remarcamos la dimensión social, ya que las EN están en continuo crecimiento debido a la pirámide poblacional europea, la cual influye directamente en este tipo de enfermedades donde el factor principal de predisposición, es la edad.

Se han tenido en cuenta dentro de los criterios de inclusión, aspectos claves que se utilizan en el día a día, no solo para el diagnóstico (el cual es meramente clínico), sino también para valorar la clínica que presentan los pacientes según avanza la enfermedad. Estos pilares fundamentales del diagnóstico y valoración son los

estadios de H&Y [29] y escala MDS-UPDRS III [38]. Y dentro de este grupo vulnerable con EP, se ha tenido en cuenta, que los efectos beneficiosos de esta intervención puedan alcanzar al mayor número de afectados por esta enfermedad. Debido a esta eficacia y eficiencia que buscamos, fundamentamos el criterio de inclusión de H&Y del estadio I-III; ya que es en los primeros estadios cuando más efectos beneficiosos pueden aportar las intervenciones terapéuticas, y la actividad física. En estadios y edades más avanzadas, los progresos son menos probables, las medicaciones dejan de tener efecto, al igual que otras terapias; y las demencias están más avanzadas.

En cada uno de estos apartados está impregnada la esencia de la responsabilidad y del deber. Responsables para responder a un área de la investigación en la que se puede obtener beneficios para el grupo vulnerable, en este caso los enfermos de Parkinson. Y es un deber, en esta ocasión que las ciencias de la actividad física sea el motor principal para llevar adelante este camino. En donde las ciencias de la actividad física y del deporte, ya de por si es un crisol de ciencias convergentes, interrelacionadas, yuxtapuestas; y en esta ocasión deja su impronta desde los inicios del diseño de esta investigación, donde establece lazos, conexiones y unión con distintas áreas del conocimiento. Desde la biología celular y molecular, hasta la valoración motora (incluyendo la evaluación y evolución clínica) y efectos de la actividad física planteada, en distintas combinaciones de capacidades físicas y funcionales.

##### Doctorando: Juan Carlos Magaña Gallardo

**Agosto 2021**

Comité de Ética de la Investigación

**CONSENTIMIENTO INFORMADO GENERAL**

Yo,……………………………………………………….,mayor de edad, con DNI,………………………….. actuando en nombre e interés propio.

**DECLARO QUE:**

He recibido información sobre el proyecto “Valoración de la función mitocondrial, utilizando fibroblastos de piel como biomarcador, en pacientes con enfermedad de Parkinson: efectos de dos programas de Actividad Física sobre la función motora, la calidad de vida, el sueño, aspectos cognitivos y el humor.” Del que se me ha entregado la hoja informativa anexa a este consentimiento y para el que se solicita mi participación. He entendido su significado, me han sido aclaradas las dudas y me han sido expuestas las acciones que se derivan del mismo. Se me ha informado/a de todos los aspectos relacionados con la confidencialidad y protección de datos en cuanto a la gestión de datos personales que comporta el proyecto y las garantías tomadas en cumplimiento del Reglamento General de Protección de Datos y de la Ley Orgánica 3/2018, de 5 de diciembre, de protección de datos personales y garantía de los derechos digitales.

También he recibido, con antelación y de forma satisfactoria, la explicación del procedimiento de extracción de Fibroblastos (Biopsia cutánea), su finalidad, riesgos, beneficios y alternativas. Que me han respondido todas las dudas, que comprendo la información recibida y que mi decisión es voluntaria.

Mi colaboración en el proyecto es totalmente voluntaria y tengo derecho a retirarme del mismo en cualquier momento, revocando el presente consentimiento, sin que esta retirada pueda influir negativamente en mi persona en sentido alguno. En caso de retirada, tengo derecho a que mis datos identificadores sean suprimidos, exceptuando que se podrían conservar si se anonimizan de manera que no se pueden vincular a mi persona.

Así mismo, renuncio a cualquier beneficio económico, académico o de cualquier otra naturaleza que pudiera derivarse del proyecto o de sus resultados.

Por todo ello,

**DOY MI CONSENTIMIENTO A:**

1. Participar en el proyecto “Valoración de la función mitocondrial, utilizando fibroblastos de piel como biomarcador, en pacientes con enfermedad de Parkinson: efectos de dos programas de Actividad Física sobre la función motora, la calidad de vida, el sueño, aspectos cognitivos y el humor.”
2. Que el equipo de investigación SAFE (Salut, Activitat física i Esport) de la Universidad de Blanquerna, el VHIR (Vall d´Hebron Institut de Recerca) y el Centro de Neurociencia y Biología celular de la Universidad de Coimbra (CNC-UC), el Investigador Principal del Proyecto el Dr. Jorge Hernández Vara, el Dr. Joel Montané (Universidad de Blanquerna) y el doctorando Juan Carlos Magaña; puedan tratar mis datos personales y difundir la información que el proyecto genere. Se garantiza que se preservará en todo momento mi identidad e intimidad, con las garantías establecidas en el Reglamento General de Protección de Datos y en la ley Orgánica 3/2018, de 5 de diciembre, de protección de datos personales y garantía de los derechos digitales y normativa complementaria.
3. Que doy mi consentimiento para el procedimiento de extracción de Fibroblastos de piel (Biopsia cutánea) y que conozco que tengo el derecho a revocarlo cuando lo desee, con la única obligación de informar al equipo médico.
4. También que las Instituciones nombradas en el punto anterior y sus investigadores y colaboradores; conserven todos los registros efectuados sobre mi persona en soporte electrónico, con las garantías y los términos legalmente previstos, si estuviesen establecidos, y a falta de previsión legal, por el tiempo que fuese necesario para cumplir las funciones del proyecto para las que los datos fueron recogidos.

En Barcelona, el / /

[FIRMA PARTICIPANTE] [FIRMA DEL IP]

HIP-Comité de Ética de la Investigación

HOJA DE INFORMACIÓN DEL PARTICIPANTE (HIP)

**Título del proyecto:**

“Valoración de la función mitocondrial, utilizando fibroblastos de piel como biomarcador, en pacientes con enfermedad de Parkinson: efectos de dos programas de Actividad Física sobre la función motora, la calidad de vida, el sueño, aspectos cognitivos y el humor.”

El investigador principal (IP) Dr. Jorge Hernández Vara del Hospital Universitari Vall d´Hebrón (HUVH), será quien llevará a cabo el reclutamiento. Responsables del servicio/grupo de enfermedades neurodegenerativas, junto a los miembros de nuestro equipo de investigación **Salut, Activitat Física i Eport (SAFE)** en un proyecto coordinado por el Dr. Joel Montané, estamos llevando a cabo el estudio: **“Valoración de la función mitocondrial, utilizando fibroblastos de piel como biomarcador, en pacientes con enfermedad de Parkinson: efectos de dos programas de Actividad Física sobre la función motora, la calidad de vida, el sueño, aspectos cognitivos y el humor.”**

Nuestro equipo de investigación desea invitarlo a participar en nuestro estudio. Antes que decida hacerlo, es extremadamente importante que comprenda por qué se está llevando a cabo esta investigación. Tómese unos minutos para leer detenidamente la siguiente información y no dude en preguntarnos lo que desee. Esta hoja de información para el participante explica el propósito de este estudio con más detalle y lo que implica si desea participar.

**¿Cuál es la finalidad de la investigación?**

El objetivo del estudio es evaluar su función mitocondrial, y como los efectos del ejercicio físico puede ayudar en el estado actual de su enfermedad.

La Enfermedad de Parkinson, y el resto de enfermedades neurodegenerativas, se caracterizan por presentar déficits en la función mitocondrial. Este estudio nos permitirá valorar en qué medida sus déficits bioenergéticos celulares asociados a su enfermedad, pueden mejorar con ejercicio físico. Para este fin utilizaremos, a los fibroblastos de piel (son pequeñas células de la superficie de su piel) como biomarcadores, ya que previamente se ha identificado que en estas células se puede evidenciar los mismos cambios que se producen a nivel neuronal. Los resultados de este estudio se obtendrán mediante la comparación, de dos grupos de pacientes en dos programas distintos de actividad física, y a la vez con un tercer grupo (grupo control) que no realizará los programas de actividad física, ni rehabilitación estándar (actualmente en el HUVH solo se lleva a cabo el seguimiento médico).

Todos los pacientes (de los 3 grupos) llevarán a cabo todas las visitas, test y los distintos procedimientos propios de la investigación.

Un estudio similar, pero sin el posible efecto terapéutico de un programa de actividad física se realizó previamente en el 2019 por uno de nuestros investigadores (no dude en consultarnos para ampliarle información).

En este estudio pretendemos determinar la diferencia de un plan de entrenamiento, para comprender como el diseño del ejercicio físico-terapéutico puede mejorar los síntomas motores y no motores, en pacientes con enfermedad de Parkinson.

Para ello pretendemos monitorear los diferentes parámetros que se enumeran a continuación, durante el tiempo en el que usted participe en el programa de actividad física (4 meses):

- Evaluación Funcional y test específicos de actividad física (3 evaluaciones)
- Valoración motora MDS-UPDRS III, función cognitiva, calidad de vida, sueño y Humor (al inicio y final, y a los 8 meses de la evaluación inicial)
- Parámetros de salud (frecuencia cardíaca, tensión arterial, medidas antropométricas, altura, peso, IMC)
- Valoración de biomarcadores de Fibroblastos de piel (2 extracciones).

Los participantes del grupo control continuarán con su práctica diaria habitual y serán entrevistados una vez por semana por los investigadores para comprobar que sus rutinas no se han alterado. El grupo control podrá recibir 4 meses de AF del programa que haya obtenido los mejores resultados, en cuanto a sintomatología, y calidad de vida; después de la última valoración (8 meses).

Su participación es totalmente voluntaria. Sin embargo, si decide hacerlo, se le pedirá que firme un formulario de consentimiento general, y otro específico respecto a la conservación de muestras en el Centro de Neurociencia y Biología celular de la Universidad de Coimbra (CNC-UC). Puede retirarse de la investigación en cualquier momento y no necesita proporcionar las causas por las cuales lo hace. También, en caso de retirada, tiene derecho a que sus datos identificadores sean suprimidos si así lo desea, exceptuando que se podrían conservar si se anonimizan de manera que no se puedan vincular a su persona.

**Si Usted decide participar en nuestra investigación …**

En primer lugar, nos aseguraremos que comprenda lo que implica la investigación y le pediremos que complete un cuestionario de salud con la colaboración de nuestro investigador (Juan Carlos), para verificar su estado actual y firme un formulario de consentimiento. Todos los participantes tendrán asignado un código que evitará la identificación directa del participante con las muestras, con las respuestas dadas en los cuestionarios, y con los Test realizados, garantizando totalmente la confidencialidad.

Luego pasaremos a las evaluaciones iniciales. La **evaluación inicial** se compone de tests funcionales y cuestionarios de salud. Estas medidas también se volverán a realizar luego de 16 semanas (4 meses) para el mejor control de su evolución. Cuatro meses después de haber terminado el estudio, continuaremos registrando sus parámetros (a los 8 meses de la evaluación inicial). El número de visitas presenciales de seguimiento serán 3: inicial, 4 meses y 8 meses).

Los resultados de sus evaluaciones se registrarán, para analizar cualquier cambio que se deba implementar, en la planificación de las actividades programadas, ejercicios, volumen e intensidad.

Las evaluaciones se realizarán en las instalaciones del VHIR, y las específicas de Actividad Física en la Universidad Blanquerna-FCS, y se mantendrá igual para el resto de las evaluaciones (final y a los 8 meses). Le enviaremos un informe a su mail de contacto.

##### FCS-Blanquerna VHIR

|  |  |
| --- | --- |
|  |  |
| Día 1.C: Cuestionarios- VHIR | |
|  |  |
| Día 1.D: Valoración motora MDS-UPDRS III- VHIR | |
|  |  |
| Día 1.E: Extracción Fibroblastos-VHIR | |

|  |  |
| --- | --- |
|  |  |
| Día 1.A: Evaluación funcional y Test de AF- FCS-Blanquerna | |
|  |  |
| Día 1.B: Evaluación de la salud-FCS-Blanquerna | |

##### Evaluación inicial

| **Evaluación funcional Y Test AF** | **Valoración motora MDS-UPDRS III** | **Evaluación de la salud** | **Extracción Fibroblastos** | **Cuestionarios** |
| --- | --- | --- | --- | --- |
| - Prueba de caminata 6´ - 1´(veces que se levanta y se sienta, en una silla) - Tiempo en levantarse de una silla, caminar   3 m, girar, caminar hacia atrás y sentarse  - Fuerza de prensión | -Valoración motora MDS-UPDRS III  (subescala motora) | - Presión arterial, FC - Altura-peso-IMC - Medidas Antropométricas   -Exploración Neurológica (Fuerza, reflejos, sensibilidad)   - Tests ortopédicos y neurodinámicos de extremidad superiores e inferiores   (EESS-EEII). | -Extracción Fibroblastos cutáneos | - (MoCA)  -Inventario de Depresión de Beck-IDB  -PD-CRS, Calidad de Vida, Humor y Sueño (SCOPA-AUT, NMS, PDSS, PDQ39,  Test de BERG, TINETTI y un cuestionario diario de caídas) |

##### FASE ESPAÑA

**PROGRAMAS DE ACTIVIDAD FÍSICA+EXTRACCIÓN DE FIBROBLASTOS MACROCICLO DE LOS PROGRAMAS DE ACTIVIDAD FÍSICA**

| *Evaluación Inicial* | *Semana 1* | 2 | 3 | *4* | *5* | *6* | *7* | *8* | *9* | *10* | *11* | *12* | *13* | *14* | *15* | *Semana 16* | *Evaluación Final* |
| --- | --- | --- | --- | --- | --- | --- | --- | --- | --- | --- | --- | --- | --- | --- | --- | --- | --- |


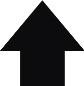


Evaluación Inicial y Final: Extracciones de muestras+cuestionarios+evaluaciones


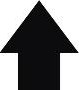


Esto se realizará a los 3 grupos (Todos los pacientes)

Control interno de la evolución de los Programas de Actividad Física.

Mesociclo 1: Periodo de adaptación individual a la carga, al volumen y a los ejercicios funcionales. Continuo Feedback Paciente-Investigador. Seguimiento semanal de la salud de los participantes.

Mesociclo 2: Primer aumento del binomio carga-volumen. Aumento de la ejecución motora en el programa (dual task training). Continuo Feedback Paciente-Investigador. Seguimiento semanal de la salud de los participantes.

|  |
| --- |
|  |
|  |
|  |

Mesociclo 3: Posibles modificaciones en base a los tests realizados en la semana 8 y 9. Continuo Feedback Paciente-Investigador. Seguimiento semanal de la salud de los participantes.

Mesociclo 4: último mesociclo de aumento en todas las dimensiones (teniendo en cuenta el principio de individualidad del entrenamiento) físicas, coordinativa y funcional Continuo Feedback Paciente-Investigador. Seguimiento semanal de la salud de los participantes.

**¿Cuáles son las medidas que deseamos tomar? E**n primer lugar, le pediremos que complete algunos cuestionarios de uso común para evaluar la calidad de vida (utilizaremos el cuestionario específico para pacientes con Parkinson-PDQ39), el estado de ánimo (Escala de Humor de Brunel (BRUMS)), la función cognitiva (mental) utilizando la Montreal Cognitive Assessment (MoCA) y otras que hemos citado, y también valoraremos aspectos del sueño. Esto nos permitirá investigar si los ejercicios que realizaremos generan un efecto positivo, además de la función motora, también en la función cognitiva, el estado de ánimo, el sueño y en la calidad de Vida.

A continuación, tomaremos diferentes medidas de salud: presión arterial, frecuencia cardíaca en reposo, altura, peso y medidas antropométricas como la circunferencia de la cintura (preferiblemente debajo de la ropa).

Luego, también nos gustaría medir su **función física** pidiéndole que complete los siguientes tests funcionales: prueba de marcha de **6 minutos** (mediremos la distancia total recorrida en metros (m) utilizando una distancia previamente fijada de 10 m, 1 minuto de **Sit-to-Stands** (contaremos el número de veces que se levanta y se sienta, en una silla, durante 1 minuto), **Timed Up and Go** (calcularemos cuánto tiempo le lleva levantarse de una silla, caminar 3 m, girar, caminar hacia atrás y sentarse - usted hará 3 intentos de esta prueba) y **fuerza de agarre** (medida máxima de fuerza de la mano izquierda y derecha). Dentro de la función física, dejaremos constancia de su valoración motora específica de la enfermedad de Parkinson (MDS-UPDRS III) realizada por los neurólogos de nuestro equipo de investigación.

Nos gustaría seguir repitiendo las medidas de la valoración funcional (Test de AF), a las 16 y a las 32 semanas.

Finalmente, hemos de extraer una muestra muy pequeña de células de la piel, para investigar su función mitocondrial (la cual se encuentra afectada en todas las enfermedades neurodegenerativas), y así poder constatar el efecto (su función y/o estructura) de la aplicación de un programa de actividad física.

**¿Qué centros/Instituciones participan y qué haremos con los fibroblastos cutáneos? Participan las siguientes instituciones:**

- **Facultad de Ciencias de la Salud-Blanquerna-Universidad Ramón Llull.**
- **Facultad de Psicología y Ciencias de la Educación y del Deporte-Blanquerna-URL.**
- **Centro de Neurociencia y Biología Celular (CNC)-Universidad de Coimbra-Portugal.**
- **Vall d’Hebron Institut de Recerca (VHIR)**

Una vez que hayamos recolectado las muestras de los fibroblastos de piel se ampliarán y congelarán en el Vall d’Hebron Institut de Recerca. Una vez finalizado el estudio, todas las muestras congeladas, serán enviadas al laboratorio de Neurociencia (CNC) de la Universidad de Coimbra Portugal, para ser analizadas.

También le pedimos su consentimiento para que autorice almacenar una parte de las muestras en la sección de Colecciones (ref C.0006794) del Registro Nacional de Biobanco para que puedan ser utilizadas en otros proyectos de investigación biomédica, nacionales o internacionales. Cualquier estudio de investigación para el que se solicite la utilización de estas muestras deberá disponer de la aprobación del Comité de Ética de Investigación con medicamentos (CEIm) del Hospital Universitario Vall d’Hebron u otros comités que se establezcan según la legislación vigente. Este comité vela para que los investigadores desarrollen sus estudios siguiendo siempre las más estrictas normas éticas y legales. La cesión de muestras y datos asociados se realizará de manera que el investigador receptor no pueda conocer su identidad.

Los datos que se obtengan de su participación, no se utilizarán con ningún otro fin distinto del explicitado en esta investigación, siendo tratadas de manera confidencial bajo la responsabilidad del investigador principal. Dichos datos quedarán protegidos mediante clave de seguridad, y únicamente tendrán acceso a los mismos, los integrantes del equipo de investigación que se detallan en este documento. Si necesita más información puede contactar con la responsable de la colección la Dra. Marta Martínez-Vicente ([marta.martinez@vhir.org](mailto:marta.martinez@vhir.org)).

**¿Cuánto tiempo llevará esto?**

Las evaluaciones funcionales y de salud tardan aproximadamente 25 minutos en completarse. Cognitivo, calidad de vida y los cuestionarios sobre el estado de ánimo tomarán aproximadamente 20-25 minutos. Y la función física otros 20-25 minutos. La extracción de los fibroblastos se hará en el mismo día.

Toda la evaluación inicial se completará durante 1 visita. Participar en todos los elementos del estudio en general puede demandar aproximadamente tres horas.

**¿Qué debo hacer antes de las evaluaciones y qué debo usar?**

Se le pedirá que evite cualquier ejercicio extenuante 48 horas antes de las visitas. También se le solicitará que evite el alcohol y cafeína (Coca Cola, té, café, chocolate, etc.) 12 horas antes de cada visita. Además, evite tomar alimentos o bebidas gaseosas al menos 2 horas antes del inicio de cada visita. Si es posible, repita sus comidas antes de cada visita a lo largo del estudio. Debe asistir con ropa adecuada y cómoda para realizar las pruebas funcionales. Le proporcionaremos agua. Traiga gafas para leer, si las necesita.

**¿Existe algún riesgo potencial?**

La extracción de las muestras de fibroblastos cutáneos, pueden causar una leve molestia durante algunas horas. Los riesgos son mínimos, además seguimos estrictos procedimientos de salud y seguridad (incluida la esterilización el área antes de tomar una muestra). Dicha extracción será realizada por uno de nuestros Neurólogos del equipo de investigación (Grupo de enfermedades neurodegenerativas) y personal experimentado del Vall d’Hebron Institut de Recerca (VHIR), utilizando procedimientos estándar de salud y seguridad. Dicha extracción será supervisada por uno de los IP del VHIR el Dr Jorge Hernández Vara, y también sumamos la experiencia de la Dra. Marta Martínez-Vicente que en otros estudios de investigación también ha obtenido biopsias de piel de pacientes.

**¿Qué pasará con los resultados de este estudio de investigación?**

Los datos recopilados se utilizarán para evaluar la eficacia de la clase de ejercicio para mejorar los parámetros de salud y la capacidad funcional. Se le proporcionarán informes sobre sus resultados personales, en un informe final. Los datos serán utilizados para publicar artículos con fines científicos, para presentaciones, conferencias nacionales e internacionales o para la elaboración de proyectos de disertación y tesis doctoral. Sin embargo, los datos personales serán anónimos y en ningún caso será posible identificar a las personas en los resultados informados para fines académicos, de evaluación o de publicación.

**¿Cuáles son los beneficios de participar en este estudio?**

En primer lugar, la información recopilada ayudará a desarrollar futuros programas de ejercicios para pacientes con EP. En segundo lugar, su participación también ayudará a arrojar luz, sobre los mecanismos por los cuales el ejercicio podría desempeñar un papel clave en la desaceleración de la progresión de la enfermedad de Parkinson. Por medio del análisis de la muestra extraída de Fibroblastos, podremos evidenciar el estado actual de su enfermedad a nivel celular. Y así, obtendrá un examen detallado de los parámetros relacionados con su salud, su función física, y su función cognitiva. Al final de la intervención, le diseñaremos un plan de entrenamiento personalizado, en base a los resultados de las pruebas realizadas (Médicas, biológicas y físicas).

Si decide participar en el estudio, es posible que del análisis de sus muestras biológicas se obtenga información relevante para su salud o la de su familia. Le entregaremos todo, junto a un informe final sobre sus resultados.

Es posible que de su participación en este estudio no se obtenga un beneficio directo. Sin embargo, la identificación de posibles factores, relacionados con la valoración de la función mitocondrial en pacientes con enfermedad de Parkinson que realizan actividad física; podría beneficiar en un futuro a otros pacientes con esta patología, y contribuir a un mejor conocimiento y tratamiento de esta enfermedad. De acuerdo con la legislación vigente, tiene derecho a ser informado de los datos que se obtengan en el curso del estudio. En el caso de que usted lo solicite, se le podrá facilitar información acerca de los estudios de investigación en los que hayan utilizado las muestras.

**Implicaciones de la información obtenida con el estudio:**

Si quiere conocer los resultados de investigación relevantes para su salud que se obtengan, infórmese a través de su médico sobre las implicaciones que esta información puede tener para su persona y su familia. Esta información se le comunicará si lo desea; en el caso de que prefiera no ser informado, su decisión se respetará. No obstante, cuando esta información, según criterio del médico responsable, sea necesaria para evitar un grave perjuicio para su salud o la de sus familiares biológicos, se informará a un familiar próximo o a un representante.

**¿Cómo se mantendrá la confidencialidad de la información recopilada?**

Cualquier información sobre usted permanecerá confidencial, anónima y resguardada por el equipo de investigación. Todos los participantes tendrán asignado un código que evitará la identificación directa del participante con las respuestas dadas en cada cuestionario, tests realizados y pruebas médicas-biológicas, garantizando totalmente la confidencialidad.

Los datos que se obtengan de su participación no se utilizarán con ningún otro fin distinto del explicitado en esta investigación, siendo tratados de manera confidencial bajo la responsabilidad del IP y de todo el Grupo de Investigación. El Investigador Principal (IP) del VHIR el Dr. Jorge Hernández Vara (HUVH), el Dr. Joel Montané (Universidad de Blanquerna) y el doctorando Juan Carlos Magaña, serán los responsables del fichero de datos personales y de realizar la codificación de datos; y el resto de los investigadores accederán solo a datos codificados. Se garantiza que se preservará en todo momento su identidad e intimidad, con las garantías establecidas en el Reglamento General de Protección de Datos. Dichos datos quedarían protegidos mediante clave de seguridad.

Toda la documentación que contenga información personal se almacenará de acuerdo con la Ley de Protección de datos. En cualquier momento se podrá dirigir al IP (Investigador Principal), a cualquiera de los miembros del equipo de investigación que se especifica más adelante en este documento, o a la institución a la cual pertenecen, para ejercer sus derechos que reconoce la Ley Orgánica 3/2018, de 5 de diciembre, de Protección de Datos Personales y garantía de los derechos digitales.

La información recopilada solo se utilizará en relación con este proyecto y no se divulgará a terceros, y únicamente tendrán acceso a los mismos el Personal Investigador (los cuales se identifican en la próxima tabla de este documento) En el anexo de protección de datos encontrará más información sobre su política de protección de datos y la forma de ejercer sus derechos.

Nos ponemos a su disposición para resolver cualquier duda. Puede contactar con nosotros a través de los mails de contacto que citamos más abajo.

**¿Quién ha aprobado este estudio?**

Este estudio ha sido revisado y aprobado por el Comité Académico del Programa de Doctorado (URL-Blanquerna) y el Comité de Ética en Investigación de la Facultad de Psicología y Ciencias de la Educación y del Deporte (FPCEE-Universidad de Blanquerna). Y también ha sido aprobado por un Comité de Ética de la Investigación con medicamentos.

**¿Y si tengo una pregunta?**

Si tiene alguna pregunta sobre cualquier aspecto de este proyecto de investigación, puede hablar con:

| Personal Investigador | Área | Correo electrónico |
| --- | --- | --- |
| IP-Dr. Jorge Hernández Vara | Hospital Universitario-VHIR | jorhernandez@vhebron.net |
| Dra. Samaniego Toro, Daniela | Hospital Universitario-VHIR |  |
| Dra. Laguna Tuset, Ariadna | Hospital Universitario-VHIR |  |
| Dra. Martínez-Vicente, Marta | Hospital Universitario-VHIR |  |
| Dra. Maria Giné | Coordinadora del Grupo SAFE (Salut, Activitat Fisica i Esport) | [mariagg@blanquerna.url.edu](mailto:mariagg@blanquerna.url.edu) |
| Dr. Joel Montané | Co-Director de esta Investigación- Fase España | [joelmm@blanquerna.url.edu](mailto:joelmm@blanquerna.url.edu) |
| Dra. Susana P. Pereira | Co-Directora de esta Investigación- Fase Portugal | [pereirasusan@gmail.com](mailto:pereirasusan@gmail.com) |
| Ldo. Juan Carlos Magaña | Personal Investigador en formación- Blanquerna | [juancarlosmg@blanquerna.url.edu](mailto:juancarlosmg@blanquerna.url.edu)  607815095 |

**En el contexto de esta investigación le pedimos su colaboración**

Ya que usted cumple los siguientes criterios de inclusión.

**Criterios de inclusión:**

-Pacientes que presenten un diagnóstico médico de EP idiopática, que se encuentren dentro de uno de los estadíos de la escala modificada de Hoehn y Yahr del I al III (incluido).

-Pacientes que tengan un buen estado cognitivo (puntuación en el Montreal Cognitive Assessment (MoCA) ≥26 puntos).

-Pacientes que hayan firmado el Consentimiento Informado.

-Pacientes con capacidad de caminar independientemente durante seis minutos.

-Edad entre 50 y 70 años.

-Pacientes con medicación estable (que no hayan tenido cambios en la medicación durante el último mes).

**Su colaboración implica participar durante 16 semanas en los programa de Actividad Física, con 3 sesiones por semana (sesiones de 1 hora), en la Universidad Blanquerna-Facultad de Ciencias de la Salud; ubicada en la** **C/ de Padilla, 326, 08025 Barcelona.**

**La Universidad (FCS)** **posee una cobertura de la póliza de responsabilidad civil, para diferentes proyectos que se llevan a cabo en la institución.**

**Y el número de visitas presenciales de seguimiento en el HUVH (Grupo de enfermedades Neurodegenerativas) serán 3: inicial (1 semana antes de comenzar los programas de AF), a los 4 meses (en la semana siguiente de haber terminado los programas de AF) y a los 8 meses.**

**Nota aclaratoria sobre el proceso de aleatorización de los distintos grupos.**

Esta investigación pretende comparar unos grupos de pacientes, en relación a los efectos de la actividad física (AF) en la función mitocondrial.

En este estudio se pretenden comparar dos procedimientos (2 programas de actividad física) y un grupo control.

La asignación vendrá determinada por el azar. Su médico no intervendrá en este proceso. Usted tendrá una probabilidad del 4,1% de recibir cada uno de los procedimientos contemplados en este estudio. Para llevar a cabo esta investigación necesitamos un mínimo de 24 pacientes diagnosticados con EP. La aleatorización de los veinticuatro pacientes, estará basada en la asignación a tres grupos (8 pacientes en cada grupo): 2 grupos realizarán programas de actividad física distintos (programa 1 y programa 2), y un tercer grupo no realizará ningún programa de actividad, conformando así un grupo de control. El grupo control podrá recibir 4 meses de AF del programa que haya obtenido los mejores resultados, una vez finalizada la intervención a los otros 2 grupos.

**Información sobre la extracción de los Fibroblastos de piel**

1. Descripción y objetivos de la biopsia cutánea

El objetivo de la técnica es el estudio de la función mitocondrial y estrés oxidativo en la Enfermedad de Parkinson (EP).

La extracción de los fibroblastos consiste en una biopsia de piel mínimamente invasiva:

- Para obtener una muestra muy pequeña de piel (aproximadamente 3 mm2) de la cara interna del brazo.

- Para la toma de biopsia de piel se le aplicará por vía tópica de un anestésico local.

- La toma de biopsias de piel puede ocasionar dolor puntual en el momento de la punción y ligeras molestias después del procedimiento.

Esta muestra de la piel, es para analizarla en el Laboratorio de Biología Celular.

Durante el procedimiento, también es posible que se hagan fotografías o se obtengan imágenes exclusivamente para fines del presente estudio; y se pixelará la cara para que los sujetos no sean identificables. Le garantizamos que su intimidad será estrictamente respetada y que las imágenes obtenidas de este procedimiento nunca irán acompañadas de datos o informaciones que puedan revelar su identidad a terceros.

2. Alternativas razonables a la biopsia cutánea

En su caso particular, se ha considerado que éste es el medio más adecuado para valorar su función mitocondrial y sus déficits bioenergéticos celulares asociados antes y después de la realización de un programa de ejercicio físico. Y que, una vez establecido este, se le indicarán las opciones terapéuticas.

En este caso no existen otras alternativas para evaluar la remodelación metabólica del estrés oxidativo, el control de calidad mitocondrial, y de proteínas (y otros parámetros) en fibroblastos cutáneos de pacientes con EP.

3. Riesgos generales y/o específicos de la biopsia cutánea

A pesar de la adecuada elección de la técnica y de su correcta realización pueden presentarse efectos poco frecuentes, en ocasiones pueden aparecer otras molestias como sangrado, o infección de la herida, y reacción alérgica a los anestésicos empleados.

Se harán todas las pruebas y tratamientos necesarios para que los riesgos de la intervención se reduzcan al mínimo.

4. Riesgos personalizados de la biopsia cutánea

Otros riesgos o complicaciones que pueden aparecer teniendo son los relacionados con sus circunstancias personales (estado previo de salud, edad, profesión, creencias, medicaciones, etc.}.

En su caso concreto, los riesgos son: ……………………………………………………………………………………………

………………………………………………………………………………………………………………………………………..

5. Conservación de muestras en el Centro de Neurociencia y Biología celular de la Universidad de Coimbra (CNC-UC)

Las muestras biológicas obtenidas en el presente estudio se conservarán durante 6 años para la realización de estudios futuros. En el laboratorio de la Universidad de Coimbra, las muestras se usarán para profundizar en los efectos del secretoma del ejercicio físico en la función mitocondrial de pacientes con EP. Además, en función de los resultados obtenidos en esta investigación, las muestras biológicas sobrantes, también podrían utilizarse como base para conseguir financiación en otros proyectos de I+D.

En Portugal, la identificación de muestras e información de datos se realizará de acuerdo con la normativa general de protección de datos, y de la misma manera que en España, se utilizarán en investigaciones que hayan recibido el informe favorable de un Comité de Ética para la Investigación; de forma que no contradiga las preferencias expresadas por usted en el consentimiento firmado y en virtud de lo establecido en los art. 6, 7, 13 y/o 14 del Reglamento (UE) 2016/279 del Parlamento Europeo y del Consejo de 27 de abril de 2016 (RGPD) relativo a la protección de las personas físicas en lo que respecta al tratamiento de datos personales y a la libre circulación de estos datos.

En Barcelona, el / / .

[FIRMA PARTICIPANTE] [FIRMA DEL IP]

***“EN INVESTIGACIÓN, Y EN LA LUCHA CONTRA EL PARKINSON...***

***…CADA PASO CUENTA”***

***Juan Carlos Magaña Gallardo***

***Personal Investigador en Formación-Blanquerna***

**“Conocerlo es el primer paso para combatirlo”**

**Lema de la Federación Española de Parkinson**

| ***Consentimiento informado específico***  *(Muestras biológicas para la investigación biomédica, y muestras biológicas sobrantes)* | **Etiqueta** |
| --- | --- |
| **Núm. SAP:** |  |
| **Unidad / Servicio:** |  |

| ***Utilización de muestras biológicas y datos clínicos obtenidos durante el proceso asistencial para investigación biomédica y conservación de muestras biológicas sobrantes en el Biobanco del HUVH y en el Centro de Neurociencia y Biología celular de la Universidad de Coimbra Portugal (CNC-UC)*** |
| --- |
| *Después de haber recibido la hoja de información del paciente y comprendido su contenido, firmo este documento y autorizo al Biobanco del Hospital Universitario Vall d’Hebron (HUVH) y al Centro de Neurociencia y Biología celular de la Universidad de Coimbra Portugal(CNC-UC):*   - *A que las muestras biológicas sobrantes de las pruebas que me han realizado o me van a realizar* - *y la información clínica y asistencial associada*   *Se utilicen con la finalidad de llevar a cabo proyectos de investigación biomédica, nacionales o Internacionales, siempre que éstos cuenten con la obligada aprobación del Comité de Ética de Investigación competente. Cualquier estudio de investigación para el que se solicite la utilización de estas muestras deberá disponer de la aprobación del Comité de Ética de Investigación con medicamentos (CEIm) del Hospital Universitario Vall d’Hebron u otros comités que se establezcan según la legislación vigente.*  *1. Autorizo que las muestras biológicas sobrantes de los fibroblastos de piel y la información clínica asociada se utilicen para investigación, en los términos recogidos en la hoja de información.*  🞏 SÍ 🞏 NO  *2. Deseo que se me comunique la información derivada de la investigación que realmente sea relevante y aplicable para mi salud o la de mi família.*  🞏 SÍ 🞏 NO  *3. Autorizo a ser contactado en el caso de necesitar más información o muestras biológicas adicionales.*  🞏 SÍ 🞏 NO |

| *PACIENTE* | PERSONA QUE INFORMA | |  |
| --- | --- | --- | --- |
| *Nombre* | *Nombre* | |  |
| *Apellidos* | *Apellidos* | |  |
| DNI  *Edad* | DNI | |  |
| *Firma*  *Fecha* | *Firma*  *Fecha* | |  |
| *REPRESENTANTE:* | | |  |
| *Nombre* | *Relación con el donante:* | |  |
| *Apellidos* |  |  |  |
| DNI | *Tipo de representante:* | |  |
| *Firma*  *Fecha* | 🞏 | (1) *Autorizado por el donante* | |
|  | 🞏 | (2) *Legalmente autorizado* | |
|  | 🞏 | (3) *Autorizado por la familia* | |
|  |  | |  |

Comité de Ética de la Investigación

***Anexo: Reglamento General de Protección de Datos (RGPD)***

*En virtud de lo establecido en los art. 6, 7, 13 y/o 14 del Reglamento (UE) 2016/279 del Parlamento Europeo del Consejo, de 27 de abril de 2016 (RGPD), el Vall d`Hebron Institut de recerca (VHIR), el Servicio de enfermedades neurodegenerativas del Hospital Universitari Vall d`Hebron (HUVH), la Facultat de Psicología, Ciències de l`Educació i de l`Esport de Blanquerna, actuando como co-encargados del tratamiento, informan que los datos obtenidos a través del presente formulario serán captados y tratados (previamente codificados) por el Centro de Neurociencia y Biología celular de la Universidad de Coimbra Portugal (CNC-UC), en la forma y modo que se reflejan en el presente formulario, así como, también informarle que la base del presente tratamiento es el consentimiento expreso de usted proporcionado en el presente documento. Los datos personales serán utilizados con las únicas finalidades descritas en el presente documento. Los mismos serán conservados de acuerdo las exigencias legales hasta la finalización de las finalidades que hayan podido motivar su captación, y/o prescripción de las acciones legales que se pudieran derivar. Los datos personales captados en el presente documento y la información facilitada podrán ser comunicados a los investigadores acreditados, así como por obligación legal a las Administraciones competentes. La falta de autorización para el tratamiento de datos y el consentimiento para la realización del procedimiento descrito comportaran la imposibilidad de realizar las tareas descritas.*

*Le informamos que tiene derecho a solicitar el acceso, rectificación, portabilidad y supresión de los datos aportados y la limitación y oposición a su tratamiento de datos.*

***¿Con quién contacto?***

***-Investigador principal/colaborador del estudio:*** *Nombre y apellido. Dr. Jorge Hernández Vara*

*Teléfono +34932746235, Correo electrónico:* [jorhernandez@vhebron.net](mailto:jorhernandez@vhebron.net)

***-Investigador Coordinador:*** *Joel Montané*

***-Datos de contacto del DPD del Centro:*** *En cumplimiento del Reglamento (UE) 2016/679, el VHIR ha designado un delegado de Protección de datos, siendo sus datos de contacto* [*dpd@ticsalutsocial.cat*](mailto:dpd@ticsalutsocial.cat)

***-Datos de contacto del DPD del Promotor:*** [*dpd@blanquerna.url.edu*](mailto:dpd@blanquerna.url.edu)

*Facultad de Psicología y Ciencias de la Educación y del Deporte-Blanquerna (Grupo de Recerca SAFE)* *El Responsable del tratamiento de los datos es la Fundación Blanquerna.*

***-Datos de contacto del promotor por correo postal:*** *Passeig de Sant Gervasi, 47 de Barcelona (CP08022*Barcelona, ………………………………………………..

Sirva la presente para solicitar **permiso a su Centro/Asociación/Fundación**

……………………………………………………………………………………. al doctorando Juan Carlos Magaña Gallardo, Personal Investigador en Formación adscripto al grupo de investigación SAFE (Salut, Activitat Física i Esport),

de la Facultat de Psicologia, Ciències de l’Educació i de l’Esport-Blanquerna. Universitat Ramon Llull, para poder realizar cuestionarios y búsqueda de información, con el fin de su investigación.

Les rogamos, colaboración de su centro, y poder así formar parte de esta investigación.

Para cualquier consulta al respecto no duden en ponerse en contacto con nosotros en el siguiente correo electrónico: [joelmm@blanquerna.url.edu](mailto:joelmm@blanquerna.url.edu)

Agradeciéndole de antemano su colaboración, reciba un cordial saludo

Fdo

Barcelona, ………………………………………………..
