# Supplementary material for: Investigating the impact of physical activity on mitochondrial function in Parkinson’s disease (PARKEX): Study protocol for A randomized controlled clinical trial
Source: PLoS One. 2023 Nov 22;18(11):e0293774. doi: 10.1371/journal.pone.0293774 (PMC10664890; doi:10.1371/journal.pone.0293774)
Supplement: S2 File — Clinical trial protocol v4, V4; December 05 2022. (DOCX) [file pone.0293774.s004.docx]

##### Facultat de Psicologia, Ciències de l’Educació i de l’Esport Blanquerna.

**Universitat Ramon Llull**

**Departament de Ciències de l’Activitat Física i l’Esport**

**Programa de Doctorat en Ciències de l’Educació i de l’Esport (Curs 2020-2021)**

**Título provisional**

**“Valoración de la función mitocondrial, utilizando fibroblastos de piel como biomarcador, en pacientes con enfermedad de Parkinson: efectos de dos programas de Actividad Física sobre la función motora, la calidad de vida, el sueño, aspectos cognitivos y el humor.”**

*The nature of this thesis arises from the main pillars of international cooperation in research networks and has its origin in the international collaboration between the University of Blanquerna (Barcelona-Spain) and the Center for Neuroscience and Cell Biology at the University of Coimbra (CNC-UC), Portugal. He will undoubtedly contribute extensively to uniting the important research areas of Mitochondrial Health Benefits of Physical Exercise and Parkinson Disease. These research areas are fundamental to human health and uniting them will be very important. There is a great need for knowledge on the role of mitochondria in disease developmental and how mitochondrial activity can be modulated in Parkinson by exercise. I feel I can say this with certainty as I was one of authors uncovering the mitochondrial bioenergetic defects in human skin fibroblasts from sporadic Parkinson’s disease patients.*

**Co-directora de Tesis:** PhD Susana P. Pereira CNC - Center for Neuroscience and Cell Biology, CIBB - Centre for Innovative Biomedicine and Biotechnology, University of Coimbra, IIIUC - Institute for Interdisciplinary Research, University of

Coimbra, Coimbra, Portugal ITR – Laboratory for Integrative and Translational Research in Population Health, LaMetEx - Laboratory of Metabolism and Exercise, CIAFEL - Research Center in Physical Activity, health and Leisure, Faculty of

Sports, University of Porto, Portugal

*La Biomedicina es un campo dedicado al avance de la medicina humana. El papel de la investigación en Biomedicina en el presente proyecto tiene un rol muy relevante, ya que se mezclan diferentes disciplinas como la Fisiología, la Medicina, la Bioquímica, las Ciencias de la Actividad Física, la Neuropsicología, entre otras. En la presente tesis doctoral, se utilizará la Biomedicina como herramienta para determinar si dos programas diferentes de actividad física mejoran, la función Mitocondrial, la función motora, calidad de vida, sueño, aspectos cognitivos y el humor en pacientes con la enfermedad de Parkinson. Este gran paso es posible gracias a la colaboración internacional, entre Center for Neuroscience and Cell Biology, de la Universidad de Coimbra, y el grupo de investigación SAFE, de la Universitat Blanquerna-URL (FPCEE).*

*Dos grupos punteros y multidisciplinares que se han unido para abordar, la valoración de la función mitocondrial utilizando fibroblastos de piel como biomarcador. Estos resultados contribuirán el campo de la Biomedicina, y ayudarán a mejorar, sin duda, la calidad de vida y el diagnóstico de los pacientes con Parkinson.*

**Co-director de Tesis:** PhD Joel Montané Facultat Ciències de la Salut Universitat Blanquerna-URL

Barcelona

##### Doctorando: Juan Carlos Magaña Gallardo

**5 de Diciembre 2022** 1

##### ÍNDICE

###### 1--Resumen o Abstract……………………………………………………………………….3 2--La justificación del trabajo……………………………………………………………….4 3--El tema y objetivos………………………………………………………………………..7 4--Los Antecedentes teóricos y estado actual del tema……………………………………..8 5-- Hipótesis y Pregunta de Investigación………………………………………………….10 6-- Diseño (Fundamentación metodológica).……………………………………………. 10

*6.1. Método e Instrumentos*

*6.2 Muestra, Criterios de selección (Criterios de Inclusión, Exclusión y Retirada), Reclutamiento, Procedimiento.*

*6.2.1Muestra, 6.2.2 Criterios de selección (Criterios de Inclusión, Exclusión y Retirada),*

*6.2.3 Reclutamiento, 6.2.4 Procedimiento*

- 1. *Intervención*
  2. *Variables*
  3. *Recogida de datos*
  4. *Análisis de datos*

###### 7--Los aspectos éticos…………………………………………………………………….…18 8--Aplicabilidad, las limitaciones y líneas futuras…………………………………………19 9--Plan de trabajo y Cronograma…………………………………………………………..20

***10--Recursos necesarios…………………………………………………………………….21 11--Investigadores……………………………………………………………………….….23 12-- Plan de Difusión, Comunicación, Presentación de Abstract, Premios………………24 13--Referencias Bibliográficas……………………………………………………………..27***

*Referencias de Anexos (En el documento “Principios básicos de la Ética aplicada a la Investigación”)*

###### 14--Anexos………………………………………………………………………………….29

-Principios básicos de la Ética aplicada a la Investigación… -C.I. General-URL … -C.I. Específico (Muestras biológicas para la investigación biomédica, y muestras biológicas sobrantes)… -Hoja evaluación de la salud del paciente (Anamnesis)… -Permisos Centros… -Anexo Aleatorización… -Folleto-Tríptico divulgación de la investigación (cara ext/int)

##### ÍNDICE DE TABLAS Y DIAGRAMAS

-Tabla 1: Prevalencia mundial de las tres principales enfermedades neurodegenerativas……………...4

-Tabla 2: Prevalencia de las enfermedades neurodegenerativas en Europa………………………….....4

-Tabla 3: Diseño AF…………………………………………………………………………………...11

-Diagrama de flujo de la investigación………………………………………………………………..13

-Tabla 4.1: Variables de estudio e instrumentos de medida utilizados………………………………..14

-Tabla 4.2: Objetivos, descripción y experimentos del Protocolo de los Fibroblastos-

FASE PORTUGAL-CNC……………………………………………………………………………..14

-Tabla 5: Estadios de la evolución de la enfermedad de Parkinson según ……………………………15

-Tabla 6: Variables de estudio…………………………………………………………………………17

-Diagrama Diseño y Plan de publicación general derivada de la tesis………………………………..21

-Tabla 7: Presupuesto Fibroblastos Fase España-IBIDELL…………………………………………...23

###### 1--Resumen o Abstract

**Introduction:** Parkinson's disease (PD) is a disorder characterized by the progressive degeneration of dopaminergic neurons resulting in dopamine deficiency in the striatum. Mitochondrial dysfunction and oxidative stress are associated with PD and are intrinsic factors related to its pathogenesis. Physical activity (PA) increases cognitive ability in older adults, attenuating motor deficits, increasing new neuron formation, ameliorating neurological impairments, and impeding age-related neuronal loss. In addition, skin fibroblasts have been identified as surrogate indicators of pathogenic processes correlating with clinical measures.

Thus, the present study aims to compare the effects of two different PA programs in PD patients analyzing relevant clinical aspects and the impact on mitochondrial function in patients' skin fibroblasts, used here as biomarker for metabolism improvement and disease progression.

**Methods:** Patients with clinical diagnosis of PD in the stages from 1 (mild) to 3 (moderate) according to Hoehn and Yahr Scale (n=24) will be recruited and randomized into three matched groups. The effects of two different PA programs will be compared. The first group (n=8) will perform basic physical training (BPT) based on strength and resistance; a second group will perform BPT combined with functional exercises (BPTFE), including exercises aimed to stimulate the specific sensorimotor pathways that are most affected in PD (proprioception-balance-coordination) together with cognitive and motor training; a third group will serve as control (sedentary group; Sed). Subjects will perform 3 sessions per week for 16 weeks. Motor function, quality of life, sleep quality, cognitive aspects and humor will be evaluated before and after intervention. A metabolic characterization of skin fibroblast will be performed by respirometry using the Seahorse XFe96, and by measuring ATP levels and mitochondria-related transcripts and proteins to determine the bioenergetic cellular deficits and characterize the metabolic remodeling induced by the exercise programs.

**Results:** We predict that the application of BPT and BPTFE programs will ameliorate relevant clinical aspects of the disease by improving systemic mitochondrial function, restoring mitochondrial metabolism, gene expression patterns, and ultimately, translating into mitochondrial neuroprotective effects.

**Conclusions:** The comparison of BPT and BPTFE PA programs will provide insights into the degree of amelioration in several relevant aspects of PD, including motor function, quality of life, quality of sleep, cognitive aspects, humour, and mitochondrial function. Such changes can have a positive clinical impact and PD skin fibroblasts may be used as a biomarker for PD diagnosis and disease progression assessment.

###### 2--La justificación del trabajo

Según la OMS [1] los trastornos neurológicos (TN) están presentes en patologías y enfermedades de millones de personas en todo el mundo. Los TN son enfermedades del sistema nervioso central (SNC) y periférico [2]. Las Enfermedades Neurodegenerativas (EN) se encuentran dentro de los TN, entre otras enfermedades. Se calcula en España entre 6 y 7,5 millones de ciudadanos sufren algún tipo de TN, (un 13- 16% del total de la población): alrededor de un millón y medio con una enfermedad neurológica grave [3]. Las EN tienen grandes consecuencias en el sistema de salud pública y en la sociedad. “Al propio proceso de la enfermedad, hay que sumar el impacto psíquico, la mengua en la calidad de vida, la incapacidad laboral, la pérdida de habilidades sociales, el gravamen de los cuidadores y las situaciones de dependencia” [4]

Un estudio realizado por Neuroalianza y la Universidad Complutense de Madrid [5] (Estudio sobre las EN en España y su impacto económico y social) señala que cada paciente gasta de media más de 23.000 euros al año.

*Tabla 1: Prevalencia mundial de las tres principales enfermedades neurodegenerativas. [5]*

| **Enfermedad** | **Casos por 100.000**  **Hab** | **Prevalencia** | **Población afectada a nivel mundial** | **Referencia** |
| --- | --- | --- | --- | --- |
| **Alzheimer y otras demencias** | 400 | 0,5% | 35.600.000 | (Prince et al., 2013, p. 63) |
| **Parkinson** | 315 | 0,34% | 23.800.000 | (Pringsheim, Jette, Frolkis, & Steeves, 2014, p. 1586) |
| **Esclerosis Múltiple** | 30 | 0,03% | 2.280.000 | (Trisolini, Honeycutt, Wiener, & Lesesne, 2010, p. 6) |

*Tabla 2: Prevalencia de las enfermedades neurodegenerativas en Europa (UE-27). [5]*

| **Enfermedad** | **Casos por 100.000** | **Prevalen** | **Población afectada en** | **Referencia** |
| --- | --- | --- | --- | --- |
|  | **Hab** | **cia** | **EUROPA** |  |
| **Alzheimer y otras demencias** | 854 | 1,23% | 6.341.179 | (Gustavsson et al., 2011, p. 729) |
| **Enfermedad de Parkinson** | 168 | 0,24% | 1.249.312 | (Gustavsson et al., 2011, p. 729) |
| **Esclerosis Múltiple** | 108 | 0,1% | 801.900 | (MSIF, 2013, p. 9) |
| **Enfermedades Neuromusculares** | 29 | 0,041% | 149.079 | (Olesen et al., 2012) |
| **Esclerosis Lat. Amiotrófica (ELA)** | 2-5 | 0,002% -  0,005% | 10.281-25.703 | (Camacho et al., 2014; Gustavsson et al., 2011) |

Las EN como el Alzhéimer y otras demencias, enfermedad de párkinson (EP), la esclerosis múltiple (EM), la esclerosis lateral amiotrófica (ELA) y las patologías neuromusculares suponen un coste total de 32.372 millones de euros anuales en España, donde viven con este tipo de problemas 988.000 personas. El 40% de los afectados dejan de trabajar por la enfermedad, y el 53% tienen dificultad económica a causa de la misma (esto sin tener en cuenta que a día de hoy habría que sumar los efectos del COVID-19). La EP es la segunda EN más prevalente en la actualidad, después del Alzhéimer [6], y pertenece a los llamados Trastornos del Movimiento. Es una enfermedad crónica, y se caracteriza por la pérdida (o degeneración) de neuronas dopaminérgicas de la sustancia negra pars compacta (SNpc) del mesencéfalo. Esta pérdida provoca una falta de dopamina en el organismo, lo cual repercute en el control del movimiento, dando lugar a los síntomas motores típicos, como el temblor en reposo o la rigidez, lentitud en los movimientos voluntarios, dificultad

de pronunciación al hablar o debilidad muscular. Aunque al día de hoy sigue siendo complejo clasificar a la

EP, y queda un amplio camino por investigar respecto a su fisiopatología, de ahí que también se la identifica como una de las enfermedades dentro de las α-sinucleopatías, debido al depósito patológico de α-sinucleína en el citoplasma de neuronas o de células gliales [7]. Estos depósitos forman las inclusiones intracelulares llamadas cuerpos de Lewy [8], que junto con la degeneración dopaminérgica de la SNpc forman la base anatomopatológica de la EP.

La AF tiene un rol muy importante y un gran impacto en la actividad cortical en los pacientes con EP [9]. Carvalho en su estudio pudo constatar que *“durante la ejecución del ejercicio físico se activaron más áreas corticales, y la principal hipótesis de este fenómeno es el aumento del flujo sanguíneo cerebral durante el ejercicio”.* En dicho estudio se evaluaron los síntomas motores de la EP (UPDRS-III), comparando 3 grupos de pacientes, en donde 2 grupos realizaron distintos programas de AF, en contraste con un grupo que realizó fisioterapia. Ambos grupos que realizaron AF mejoraron sus síntomas motores un 27,5% (valorados Pre y Post con UPDRS-III), mientras que el grupo de fisioterapia mostró una mejora del 2,9%. Al finalizar la intervención, los tres grupos mejoraron su capacidad funcional.

Esta investigación nace de la necesidad de conectar las investigaciones de la biología celular, en especial aquellas que hacen referencia a la disfunción mitocondrial y el estrés oxidativo (características en común de varias EN), junto con procesos bioenergéticos metabólicos; y la clínica que presentan los pacientes con EP en distintos programas de actividad física (AF).

La relevancia de los fibroblastos de piel como patrón surrogado en esta investigación, se da por la capacidad que tienen de reflejar los déficits bioquímicos típicos de las neuronas nigrales [10]. Adelantándonos al “marco teórico” citamos a Milanese que nos comenta que dichos déficits “son factores de sostenimiento del proceso neurodegenerativo de la EP, y pueden ser detectados en fibroblastos de pacientes con Enfermedad de Parkinson esporádico (EPs)”. De ahí la importancia como biomarcador surrogado a los procesos patógenos, ya que sería posible correlacionar con las medidas clínicas, como por ejemplo la función motora por medio de la MDS-UPDRS III. Por lo tanto, buscamos correlacionar y entender que procesos ocurren a nivel mitocondrial por causa de la AF, que hace que los síntomas motores y no motores, mejoren y/o frenen su avance después de una intervención.

Existen estudios recientes (Deus et, al; 2020) en el que se analizaron (células no neuronales) fibroblastos de piel de pacientes con EPs, para detectar alteraciones metabólicas y mitocondriales que también existirían en un tipo de célula no neuronal [11]. Estudios como el que acabamos de citar, son los que han ensanchado el camino de nuestra investigación, y han reforzado aún más, la certeza de utilizar los fibroblastos de piel en pacientes con EP; y poder así investigar los beneficios del ejercicio físico para la salud mitocondrial en la EP. Todo esto nos ha conducido a iniciar la investigación, sobre los efectos de la AF, y el análisis de los fibroblastos de piel, para poder valorar la función mitocondrial de los pacientes; y también como la AF influye en los déficits proteolíticos y bioenergéticos celulares, presentes en todo proceso neurodegenerativo de la EP [10].

Esta investigación, pretende ser clave para valorar la función mitocondrial de una manera que aún no se ha realizado, es decir, antes y después de una intervención de AF en pacientes con EP.

Las mitocondrias tienen la capacidad de oxidar sustratos y producir energía en el ciclo de Krebs y en la cadena de transporte de electrones (ETC). Cuando el metabolismo mitocondrial de los sustratos es más eficiente, se optimiza la producción de energía, y se oxida mayor cantidad de lactato dentro de ellas [12]. Las mitocondrias consumen la mayor parte del oxígeno (el 90% según R. A‐Perez 2020) a través del Complejo IV de la ETC [13]. Todo el complejo mecanismo de la ETC es esencial, para el metabolismo celular dependiente de estas organelas. La ETC transforma la energía de los nutrientes y crea una ruta de gradientes de protones y electrones con el fin de producir ATP.

La importancia de las mitocondrias se debe principalmente a su función, son como una fábrica de energía en casi todas las células; y regulan la producción de las especies reactivas de oxígeno (ROS) y los procesos de muerte celular. Lo hacen mediante la combinación de oxígeno con las moléculas de combustible (azúcares y grasas). Esta es la razón (relación Estructura-Función) por la que nos interesa medir el consumo de oxígeno en los fibroblastos de piel, la llamada respirometría, que tiene por objetivo principal medir la función mitocondrial. Lo que N. Carter denomina la medida estándar de oro [14].

En la EP la función mitocondrial se encuentra afectada, las células no tienen suficiente energía, las moléculas de oxígeno y combustible no utilizadas se acumulan en las células, causando daños. Las células musculares y nerviosas tienen necesidades altas de energía, por lo que los problemas musculares y neurológicos son comunes. En sujetos sanos se sabe que, a mayor eficiencia mitocondrial, disminuye la producción de radicales libres y se mantiene la integridad de las proteínas, lípidos y ADN mitocondrial [15]. Teniendo en cuenta que la función mitocondrial es una referencia fundamental para comprender el metabolismo en la salud y en la enfermedad. Uno de los “puentes” que encontramos (de las múltiples conexiones encontradas) en el trinomio Función Mitocondrial-EP-AF, son las Mitocondrias. Y para ello en los principios de este plan de recerca hemos profundizado primero desde la salud, y en lo que respecta a los valores fisiológicos normales y las distintas vías e impactos de la AF. Hemos visto que existe una relación entre la biogénesis mitocondrial y el ejercicio físico, por ejemplo, una de las adaptaciones que produce el entrenamiento aeróbico es el aumento del número y tamaño de las mitocondrias.

Hoppeler (1985) demostró que con entrenamiento aeróbico de 6 semanas de duración 5 veces por semana de 30’ cada sesión con intensidad de 4 mMol de lactato hubo un aumento en promedio de 40% de estas organelas, tanto subsarcolémicas como intermiofibrilares [16].

En sujetos entrenados se encontró una doble de cantidad de mitocondrias por mm3 respecto a los no entrenados. Cabe destacar que la señal para iniciar la biogénesis de las mitocondrias es la contracción muscular, las mitocondrias no aumentarán si no se recluta la fibra muscular. Esto es una de las bases de porqué hemos elegido como objetivo transversal en ambos programas de entrenamiento, el trabajo no solamente aeróbico, sino también el trabajo de la Fuerza. Con los objetivos de mejorar la disminución del

estrés oxidativo y el aumento de la capacidad antioxidante.

Por otra parte, la relación de la actividad física con algunos aspectos positivos a nivel neurológico [17], sostienen que la ejercitación del cuerpo humano “parece activar una serie de procesos encargados de mantener y proteger a las células nerviosas, lo que podemos llamar sistemas de neuroprotección fisiológica”. La actividad física favorece a que se produzcan unos mecanismos de compensación mediante una reorganización de los circuitos neuronales dañados. Vera Hinojosa y Flores [18] detallan que, “es necesaria la AF en el cuerpo humano para sostener una serie de funciones básicas, ya que para el movimiento de éste mediante el ejercicio, el cerebro se activa en múltiples zonas, básicamente asociadas a: la coordinación del movimiento correcto de los músculos implicados en el ejercicio, producción y liberación de Myokinas [19]; el aumento del flujo sanguíneo; la administración del consumo de glucosa; el control de la respiración y del ritmo cardíaco, la capacidad del sistema sensorial, entre tantos otros [14]. Por lo tanto, un estilo de vida sedentario es un factor de riesgo para muchas patologías crónicas. Es vital incrementar los niveles de condición física en personas con EN, y en particular en personas con EP. Esto nos lleva a estudiar la eficacia de la AF como intervención terapéutica, y así poder investigar cómo influyen los beneficios del ejercicio físico a nivel mitocondrial en este tipo de población.

Ya expuestas las tres áreas principales de conocimiento en las que interactúa esta tesis (Ciencias de la AF- Ciencias de la Salud (EP)-Biología Celular y Molecular), y la breve introducción respecto a la prevalencia de la EP; solo nos cabe agregar en este apartado el papel de la Biomedicina. El Dr. Soria Bernat [20] nos adelanta que la investigación biomédica en España tendrá un gran protagonismo en el escenario de los grandes avances del presente siglo. Y a pesar que en la actualidad hay una demanda creciente de investigación científica, el Dr. Soria nos ha advertido del “desfase en el lugar que España ocupa en Europa desde un punto de vista económico y el lugar que ocupa en la investigación, en general, y en biomedicina, en particular” [20]. Actualmente las iniciativas para contrarrestar este desfase se basan en promover y desarrollar la investigación en Biomedicina que incluyen sinergias y colaboraciones entre Universidades, instituciones y demás organismos como el Instituto de Salud Carlos III y los Centros de Investigación Biomédica en Red (CIBER). Tales acciones y dichos centros han permitido a España disminuir el desfase respecto al resto de los países de Europa. Citando al Dr. Soria “es esencial que el resultado del conocimiento llegue a la práctica clínica y se convierta en terapias efectivas.”. Es así que, para llegar al resultado del conocimiento, en esta tesis proponemos el camino de la AF en su forma de ejercicio terapéutico para analizar su efectividad (mejora de la función mitocondrial) en estrecha relación con la práctica clínica; y pueda ser devuelta a la sociedad en caminos de abordajes efectivos en pacientes con EP. Y que también el conocimiento desentrañado pueda ser referente para el resto de las EN.

###### 3--El tema y los objetivos

**Tema**

El tema de esta Tesis consiste en analizar los efectos de la intervención de dos programas distintos de AF en pacientes con EP, y poder así comprender las repercusiones de los beneficios del ejercicio físico a nivel mitocondrial, para poder comparar con la disfunción mitocondrial en pacientes sedentarios (Grupo control).

Se ha demostrado que el ejercicio físico es una herramienta terapéutica que frena el ritmo del deterioro y el avance de la EP. Y como en la fisiopatología de la EP está presente la disfunción mitocondrial, por lo tanto, hemos visto que el próximo paso a dar en el terreno de la investigación, luego del estudio de Deus, et, al 2020 [11], es investigar, utilizando a los fibroblastos de piel como herramienta de valoración, pero en este caso con pacientes que hayan seguido un plan de AF. Nos hemos propuesto analizar las rutas metabólicas del ejercicio. La evidencia científica ha demostrado que el estado energético de las células controla muchas reacciones metabólicas (Gomes et al., 2011), también que la actividad física repercute beneficiosamente en ese estado energético y por ende en sus reacciones metabólicas.

Hasta donde sabemos, esta es la primera investigación que estudia los efectos de la AF supervisada, sobre la función mitocondrial, cogiendo a los fibroblastos de piel como biomarcadores de toda la respirometría mitocondrial, en personas con EP.

##### Objetivo General

-Valorar los efectos de la AF en la función mitocondrial, de pacientes con enfermedad de Parkinson, que realizan distintos programas de AF, y sus efectos sobre la función motora, la calidad de vida, el sueño, aspectos cognitivos y el humor.

##### Objetivos Específicos

-Valorar posibles cambios en los déficits proteolíticos y bioenergéticos celulares.

-Relacionar la clínica que presentan los pacientes con EP, para valorar la evolución en los distintos programas de AF, respecto a los estadios de H &Y, y función motora (MDS-UPDRS III)

-Valorar los efectos positivos de la mejora de la condición física y del sistema cardiovascular, en la función motora, la calidad de vida, el sueño, aspectos cognitivos y el humor.

-Mediante una revisión bibliográfica (que realizaremos) determinar qué programas de AF serían los más beneficioso para mejorar la función mitocondrial, la función motora, la calidad de vida, el sueño, aspectos cognitivos y el humor, en pacientes con EP.

-Realizar una caracterización metabólica de fibroblastos cutáneos de pacientes con EP y evaluar los efectos de la remodelación metabólica mediante el ejercicio sobre el estrés oxidativo, el control de calidad mitocondrial, el número de copias de ADN mitocondrial, proteínas y transcripciones en fibroblastos cutáneos de pacientes con EP.

**-**Correlacionar la función mitocondrial de los fibroblastos de piel de los pacientes con EP, para desarrollar un biomarcador biológico de baja invasión, para el diagnóstico y la progresión de la EP.

###### 4--Los Antecedentes teóricos y estado actual del tema

En el marco de nuestra investigación y respecto al abordaje clínico de la EP, nos centramos en una de las 3 áreas de intervención clínica, que es la Actividad Física (en modalidad de ejercicio terapéutico). Las otras 2 áreas vienen a ser la Farmacológica y la intervención quirúrgica. Actualmente hay un gran auge y mayores avances en los estudios de la biología celular y molecular, mecanismo mitocondrial (estrecha relación entre estructura y función), señalización, y procesos metabólicos. Ya hemos ido avanzando en el apartado 2 y 3

(“Justificación” y “Tema”) datos importantes respecto al estado de la cuestión. En las conclusiones de Bloomer [21] vislumbramos una llamada a investigar en esta área, ya que se sorprenden de que ningún estudio hasta la fecha ha investigado el papel del ejercicio estructurado para mejorar el estado oxidativo en personas con EP. Bloomer se centró en estudiar a nivel sanguíneo los efectos del entrenamiento de resistencia sobre el estrés oxidativo en la EP.

Hasta el momento no se han encontrado estudios que relacionen la clínica de la EP, que utilicen como biomarcadores a las organelas celulares (por medio de los fibroblastos) analizando el impacto de distintos tipos de AF, y cómo influyen en los déficits de la bioenergética celular. Esta es la primera investigación que estudiará los efectos de la AF, en la disfunción mitocondrial, utilizando el modelo de estudio de los fibroblastos de piel en personas con EP. “El músculo esquelético funciona como un órgano endocrino, puede producir y secretar cientos de Miokinas, con señales autocrinas, paracrinas o endocrinas. Los avances recientes muestran que el músculo esquelético produce Miokinas en respuesta al ejercicio, lo que permite la diafonía entre el músculo y otros órganos, incluido el cerebro. Actualmente se han identificado que las funciones biológicas de las Miokinas tienen efectos sobre, la cognición, el metabolismo de lípidos y glucosa, entre otras. En relación a esta tesis. nos sugiere que las Miokinas pueden ser biomarcadores útiles para monitorear la prescripción de AF para personas con EN [19] ".

El estudio del sistema nervioso humano, posee la complejidad y dificultad debido a la inaccesibilidad del tejido, sumado a las limitaciones de los modelos celulares y animales de reproducir su fisiología. En lo que respecta a las investigaciones de mecanismos moleculares relacionadas con el envejecimiento y con las EN, hace años que se encuentran utilizando animales modificados genéticamente, pero que no terminan de reproducir del todo las patologías ni los fenotipos asociados a la vejez. Citando parte de una de las conclusiones del estudio de Ambrosi [10] “la investigación de los mecanismos patogénicos en las células periféricas, como los fibroblastos derivados de pacientes con EP esporádica y controles emparejados por edad/sexo, podría generar una comprensión más profunda de los déficits que afectan a las neuronas dopaminérgicas y, posiblemente, nuevas herramientas aplicables a la práctica clínica.” Es así que, en medio de esta conclusión, nuestra investigación pretende incorporar/aportar una nueva variable, que será analizada por medio de los fibroblastos; para obtener nuevas conclusiones/resultados. Y poder aportar evidencia a nivel celular y molecular de cómo influye los distintos programas de AF en pacientes con EP.

En la búsqueda bibliográfica (*estat de la qüestió i marc teòric*), hasta el momento hemos encontrado como denominador común [10, 22], en aquellas investigaciones que utilizan los fibroblastos cutáneos de pacientes con EP, todos coinciden que es una fuente fácilmente accesible de células en proliferación, que comparten la misma complejidad genética de las neuronas [23], muestran déficits bioquímicos típicos de las neuronas nigrales, déficits que están relacionados con el sostenimiento del proceso neurodegenerativo de la EP, también son capaces de reflejar el daño celular acumulativo a la edad del paciente [10]. Varios autores señalan, que las investigaciones de biomarcadores periféricos podrían basarse en los fibroblastos cutáneos de

pacientes con EP [10, 24].

Milanese y asociados en su investigación [24] sostienen que existe una relación entre el cuadro clínico de los pacientes con EPs y la función mitocondrial periférica, lo cual nos da un punto clave en la fundamentación de esta investigación en el programa de doctorado.

###### 5-- Hipótesis y Pregunta de Investigación

- **Hipótesis nula:** el ejercicio físico no resultará beneficioso en la mejora de la función del metabolismo mitocondrial en los fibroblastos de piel en pacientes con EP, como así también no tendrá efectos positivos en la función motora, aspectos cognitivos y emocionales, como el humor; y la calidad de vida y el sueño.
- **Hipótesis alternativa:** el ejercicio físico resultará beneficioso en la mejora de la función del metabolismo mitocondrial en los fibroblastos de piel en pacientes con EP, como así también tendrá efectos positivos en la función motora, aspectos cognitivos y emocionales, como el humor; y la calidad de vida y el sueño.

**Pregunta de Investigación:** ¿Puede el ejercicio físico restaurar y/o mejorar la función del metabolismo mitocondrial en los fibroblastos de piel de los pacientes con EP, como así también mejorar la función motora, aspectos cognitivos y emocionales, como el humor, la calidad de vida y el sueño?

###### 6--Diseño (Fundamentación metodológica)

**La Metodología: método e instrumentos**

**Fundamentación Metodológica**: Básicamente esta investigación ha adoptado desde sus inicios la metodología cuantitativa. El diseño de la investigación estaría dentro de un marco Analítico experimental, prospectivo (Diseño y Plan de publicación general derivada de la tesis página 23). La metodología cuantitativa participa en cada una de las valoraciones principales de esta investigación, tanto del objetivo principal, como de los objetivos secundarios. Por la parte de la valoración mitocondrial, se toma a la respirometría como la “medida estándar de oro”, basándose en los resultados que arrojarán los análisis Seahorse al ser procesados con el software Wave (Agilent). La esencia de la fundamentación metodológica por medio de la metodología cuantitativa, está dada en que la valoración que realizaremos del impacto de la AF en los fibroblastos de piel, se basa en pruebas de laboratorio que utilizan medios químicos, físicos y bioquímicos principalmente. Y así poder medir directa o indirectamente, los distintos procesos bioenergéticos, que puedan detectar alteraciones bioquímicas para compararlas Pre y Post-intevención.

Y la función motora también es un claro ejemplo del aspecto cuantitativo, por medio de la escala MDS- UPDRS III.

**6.1. Método:** Se ejecutarán 2 tipos de programas distintos de AF. El primero se basará solo en el trabajo de las capacidades físicas básicas (CFB), específicamente se trabajará la Fuerza (F) y la Resistencia (R). El segundo consistirá en CFB (F y R se trabajará transversalmente en ambos programas) + estimulación de las vías sensomotoras específicas que se encuentran más afectadas (Propiocepción-Equilibrio-Coordinación). Sumando el componente cognitivo-motor (“dual task training”), en el cual se verá involucrada la marcha ida y vuelta, con ejercicios coordinativos, y en los extremos se realizará una actividad cognitiva.

**Datos-Sujetos**

-Participantes:…….

-Turno:……..

**CFB PROGRAMA MIXTO FUERZA-RESISTENCIA** (Referencia+Sinergia = Tendrá más peso (en el Macro-meso-micro-ciclo) lo que dio más resultados positivos en la referencia citadas, más otras que se analizarán al hacer la revisión bibliográfica.

##### Referencia+Sinergia Descripción Resultados que fueron potenciados

-EP (HY I-III) CFB: …...(±DE) CFB-EF: ……(±DE)

-Edad: …… ± ……

-Duración EP:

…… ± ……

- MMSE: …… ± ……

-Pacientes “on” (entrenamiento y evaluación)

**-Miyai et al.,2000 [25]**

***Pros:**

1. **El estudio en corto plazo, BWSTT mejora más la movilidad que AG.**
2. **BWSTT fue más efectivo para mejorar las puntuaciones UPDRS, velocidad de marcha y longitud de paso**

**Steffen etal., 2012 [26]**

***Contras: Intervención: 10 meses**

***Pros:**

**-Mejoró la categoría de “estado mental, comportamiento y humor” de UPDRS**

-**Body weight–supported treadmill training** BWSTT

**(entrenamiento en tapiz con soporte parcial del peso)**

-**Acondicionamiento general**, ejercicios de amplitud de movimiento, entrenamiento de actividades de la vida diaria y entrenamiento de marcha.

Programa de entrenamiento:

-Caminar en tapiz hacia delante: facilitó resistencia, velocidad y longitud de zancada. Entrenamiento en tapiz: entre 2.7 y 4.8 km/h,pendiente 0%.

-Caminar en tapiz hacia atrás.

-Actividades en esterilla, para movilidad y fortalecimiento de cadera y columna.

-Calidad de vida-PDQ-39

-MDS-UPDRS-III (examen motor) Rendimiento funcional:

-Resistencia y velocidad marcha: 6MWT

-Equilibrio: BBS

-Movilidad funcional: TUG

Rendimiento funcional:

-Resistencia y velocidad marcha: 6MWT

-Equilibrio: BBS

-Movilidad funcional: TUG

-Las sesiones formarán parte del programa principal, y serán adaptadas por el

CFB: Capacidades Físicas Básicas DE: Desviación estándar

**CFB-EF PROGRAMA MIXTO+EJERCICIOS FUNCIONALES** (Referencia+Sinergia = Tendrá más peso en el (Macro-meso-micro-ciclo) que dio más resultados positivos en la referencia citada.

investigador según

las necesidades individuales.

**Gobbi et al.,2009 [27]** Ejercicios de modo múltiples

-Capacidad funcional, capacidad aeróbica, flexibilidad, fuerza MMSS y MMII, coordinación y equilibrio.

-Movilidad funcional-TUG

-Equilibrio funcional-Escala de Berg (FBS)

-MMSE -HY -UPDRS

POR ESTA RAZON Y POR LAS MEDIDAS DE PREVENCIÓN COVID, PROPONEMOS QUE EL RATIO SEA DE 5:1

Y que en un mismo grupo de

intervención se

**Tanaka et al., 2009 [28]**

**-Programa de ejercicio físico que se llevó a cabo en 6 meses y se trabajó Coordinación-Fuerza- Equilibrio**

**Texeira et al, 2014, también llevó a cabo el estudio en 6 fases**

-Sesiones con componente aeróbico

-Se trabajó estiramientos (flexibilidad), fuerza muscular, coordinación y el equilibrio mediante actividades motrices recreativas.

-La carga fue creciendo al final de cada fase.

-Se evidenció mejora en las funciones ejecutivas

pueda crear el turno A y B

CFB-EF: Capacidades Físicas Básicas-Ejercicios Funcionales*Tabla 3: Diseño de AF*

Los 2 programas de AF se compararán con el grupo control. Se definirá cuál de los programas proporciona cambios más positivos en la función mitocondrial, en la bioenergética celular y toda la información relevante de los fibroblastos cutáneos. Teniendo en cuenta cómo evoluciona en la clínica que presenta cada paciente; la función motora, aspectos cognitivos, el humor, la calidad de vida, y el sueño. Se realizarán tests al inicio (Pre-intervención), en la parte media y final de la intervención (2 y 4 meses) y tests físicos-funcionales (a los 8 meses). Las mediciones y la intervención se realizarán con pacientes en estado "on".

En la parte metodológica respecto a los fibroblastos cutáneos y ciertos controles clínicos y de laboratorios se llevarán a cabo (primera Fase-España) bajo la supervisión del Investigador Principal (IP)-Spain y Co- dirección de Tesis del Dr Joel Montané; en la FCS-Blanquerna de la Universitat Ramon Llull (URL) y en los **centros colaboradores** del Grupo de Enfermedades Neurodegenerativas del Hospital Universitario-Institut de Recerca Vall d’Hebron **(VHIR)**, Barcelona. Mediante la supervisión del Dr. Jorge Hernández Vara (Neurólogo), con la colaboración de la neuróloga Daniela Samaniego Toro, y las investigadoras Dra. Marta Martínez-Vicente y Dra. Ariadna Laguna Tuset. En dicho centro se realizarán las biopsias de piel por uno de los neurólogos del Grupo de EN del VHIR (al inicio y final de la intervención en los 3 grupos) y amplificación de los fibroblastos. El posterior cultivo y procesamiento (segunda Fase-Portugal) estarán cargo del Center for Neuroscience and Cell Biology (Universidad de Coimbra) en el cual la IP-Portugal y Co-directora de esta tesis la Dra. Susana P. Pereira, es una de las investigadoras principales de dicho centro. En esta segunda fase (Fase Portugal) tendrá lugar la Estancia en Centros Internacionales (ECI), por parte del doctorando (Mobilitat del doctorand, estades en centres de recerca).

##### El protocolo de los Fibroblastos:

La planificación general se basa en recolectar muestras en España, aislar los fibroblastos y expandirlos en elVHIR (3 millones de células), y enviar 1 millón de células a Portugal.

##### Fase España

-Recolectar las biopsias de piel, aislar los fibroblastos y cultivar para expandirlos (aumentar el número de células) hasta obtener 3 millones de células (Dra. Susana P. Pereira). El transporte lo hará una empresa especializada (Presupuestos pedidos a Fedex, Polar-Express y Lab-courier).

##### Fase Portugal (Descripción General)

-Con las células en Portugal, se pueden volver a cultivar y expandir para hacer los experimentos, por lo que será posible aumentar el número de células para varios experimentos y congelarlas si es necesario. Se realizarán análisis de Seahorse, que le dan la función mitocondrial (parámetros en la Fig.1 en el artículo de Milanese 2019) [24]. También se determinará el potencial mitocondrial con la sonda TMRM, estrés oxidativo celular (por ejemplo, usando rojo amplex y otras sondas), y actividades de enzimas antioxidantes o contenido de proteínas y/o niveles de ARNm. Se evaluará en la dinámica mitocondrial, el control de la calidad de las proteínas, evaluando la eficiencia del Sistema de Proteasoma de Ubiquitina (UPS), los niveles de proteína de los marcadores autofágicos, y los cambios en la bioenergética celular.

-En base a los resultados, se podrá investigar algunas vías específicas relacionadas con el ejercicio. 12

Versión 4, 05/12/2022

##### FASE ESPAÑA

**PROGRAMAS DE ACTIVIDAD FÍSICA+EXTRACCIÓN FIBROBLASTOS+EVALUACIONES**

**FASE ESPAÑA**

(IP-Spain) y

Co-dirección de Tesis

Dr. Joel Montané

-Mesociclo 1

-Mesociclo 2

-Mesociclo 3

-Mesociclo 4

##### MACROCICLO DE LOS PROGRAMAS DE ACTIVIDAD FÍSICA

|  | | | | | | | | | |  | | | | | | | | |
| --- | --- | --- | --- | --- | --- | --- | --- | --- | --- | --- | --- | --- | --- | --- | --- | --- | --- | --- |
|  |  |  |  |  |  |  |  |  |  | |  |  |  |  |  |  |  |  |
| *Evaluación Inicial* | *Semana 1* | 2 | 3 | *4* | *5* | *6* | *7* | *8* | *9* | | *10* | *11* | *12* | *13* | *14* | *15* | *Semana 16* | *Evaluación Final* |

-Inicial y Final: Extracciones de muestras+cuestionarios+evaluaciones

Esto se realizará a los 3grupos

-Evaluación Intermedia: Solo Tests Físicos (Eval Funcional) +

Anamnesis de salud

| **Evaluación funcional Y Test AF** | **Valoración motora MDS-UPDRS III** | **Evaluación de la salud** | **Extracción Fibroblastos** | **Cuestionarios** |
| --- | --- | --- | --- | --- |
| - Prueba de caminata 6´ - 1´(veces que se levanta y se sienta, en una silla) - Tiempo en levantarse de una silla, caminar   3 m, girar, caminar hacia atrás y sentarse  - Fuerza de prensión | -Valoración motora MDS-UPDRS III  (subescala motora) | - Presión arterial, FC - Altura-peso-IMC - Medidas Antropométricas   -Exploración Neurológica (Fuerza, reflejos, sensibilidad)   - Tests ortopédicos y neurodinámicos de extremidades superiores e inferiores   (EESS-EEII). | -Extracción Fibroblastos cutáneos | - (MoCA)  -Inventario de Depresión de Beck-IDB  -PD-CRS, Calidad de Vida, Humor y Sueño (SCOPA-AUT, NMS, PDSS, PDQ39,  Test de BERG, TINETTI y un cuestionario diario de caídas) |

## FASE

(IP-Portugal) y

Co-dirección de tesis

Día 1.A: Evaluación funcional y Test AF FCS-Blanquerna

Día 1.D: Valoración motora MDS-UPDRS III VHIR

Día 1.B: Evaluación de la salud FCS-Blanquerna

Día 1.E: Extracción Fibroblastos-

Hospital Universitario-VHIR

Día 1.C:

Cuestionarios-VHIR

## PORTUGAL

Dra. Susana P. Pereira

**Hospital Universitario-VHIR**

**Extracción Fibroblastos-**

**VHIR**

- Derivación, expansión y criopreservación de fibroblastos.

***Evaluación Funcional:** Miokinas en respuesta al ejercicio [19]

**Centro de Neurociencia y Biología Celular (CNC)- Universidad de Coimbra- Portugal**

**Empresa de transporte**

**especializada**

Valoración Mitocondrial

- Aislar los fibroblastos, cultivarlos para expandirlos (aumentar el número de células) hasta obtener 3 millones de células (por muestra recogida)


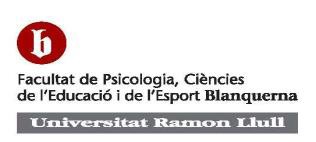

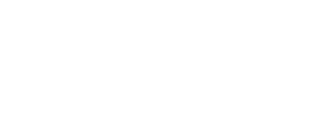

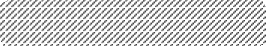

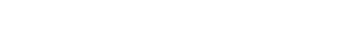

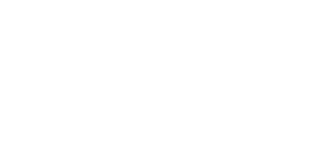

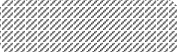

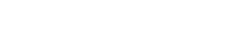

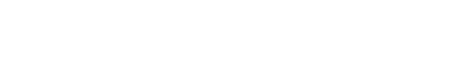

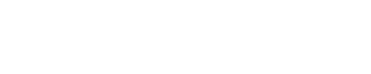


**Estancias en Centros Internacionales**

Respirometría

Rutas metabólicas del ejercicio en EP


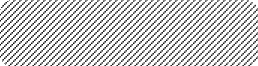

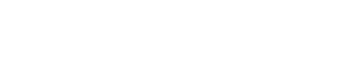


13

**Instrumentos:**

*Tabla 4.1: Variables de estudio e instrumentos de medida.*

| **VARIABLES DE ESTUDIO** | **INSTRUMENTOS PARA MEDIR LA VARIABLE DE ESTUDIO** |
| --- | --- |
| **Fuerza muscular de MMSS y MMII** | -Dinamómetro. |
| **Evaluación functional-Equilibrio** | -Test de Berg (BBS), TINETTI y un cuestionario diario de caídas. |
| **Evaluación funcional -Marcha** | -Test de caminar 6 Minutos (6MWT). |
| **Evaluación funcional -STS** | -Veces que se levanta y se sienta, en una silla, 1´ (Sit-To-Stands). |
| **Evaluación funcional-TUG** | Tiempo en levantarse de una silla, caminar 3 m, girar, caminar hacia atrás y sentarse (Timed Up and Go). |
| **Función motora** | -Escala de Clasificación de la Enfermedad de Parkinson  Unificada de la Sociedad de Trastornos del Movimiento(MDS-UPDRS-III). |
| **Aspectos cognitivos** | -MMES - PD-CRS -Clock Drawing Test (CDT) -MoCA  -Trail Making Test A (TMT-A) and B (TMT-B). |
| **Calidad de vida** | -Cuestionario de la Enfermedad de Parkinson (PDQ-39). |
| **Humor** | -Escala de humor BRUMS. |
| **Sueño** | - Sueño (SCOPA-Sueño) - Parkinson's disease sleep scale (PDSS) (Chaudhuri et al, 2002). |
| **Respirometría**-**Cuantificación del consumo de**  **oxígeno celular** | -medir la tasa de consumo de oxígeno (OCR) y la tasa de acidificación extracelular (ECAR) con los analizadores Seahorse XF-Analizador de flujo extracelular Seahorse XF e 96. |
| **Depresión** | Inventario de Depresión de Beck-IDB |
| **Caracterización metabólica de fibroblastos** | -Análisis de datos de Seahorse-Se utilizará el software Wave (Agilent) |

*Tabla 4.2.: Objetivos, descripción y experimentos del Protocolo de los Fibroblastos-FASE PORTUGAL-CNC.*

| **OBJETIVO GENERAL DEL ESTUDIO** | **OBJETIVOS ESPECIFICOS** | **DESCRIPCIÓN** | **BIOLOGÍA MITOCONDRIAL INSTRUMENTOS** |
| --- | --- | --- | --- |
| Evaluar el remodelado mitocondrial en los fibroblastos de la piel de pacientes con enfermedad de Parkinson (EP) cuando se somete a una intervención de Actividad Física; y los respectivos controles de la misma edad | Evaluar los efectos de la remodelación metabólica sobre el estrés oxidativo, control de calidad mitocondrial, número de copias de ADN mitocondrial, proteínas y transcripciones. | En esta tarea caracterizaremos los efectos del remodelado del ejercicio físico, en las células de fibroblastos de pacientes con EP, en términos de alteraciones epigenéticas, biogénesis mitocondrial, estrés oxidativo y control de calidad mitocondrial. | 1. qRT-PCR para transcripciones de interés relacionadas con la biogénesis mitocondrial, dinámica, regulación epigenética, fosforilación oxidativa, estrés oxidativo (SOD1, SOD2, NFE2L2), mecanismos de control de calidad, incluyendo autofagia y UPS. 2. Transcripciones mitocondriales, RT-PCR se realizará utilizando el SsoFast Eva Green Supermix, en un sistema CFX96 de PCR en tiempo real (Bio-Rad, Hercules, CA, EE. UU.) 3. Regulación y abundancia de proteínas mitocondriales, inmunotransferencia usando Trans-Blot Turbo Transfer System (Bio-Rad) y UVP BioSpectrum 500 Imaging System (UVP, Upland, California). 4. Actividades enzimáticas, lector de microplacas multimodo Cytation3 (BioTek Instruments, Inc.). 5. Complejo I y actividad citrato sintasa, usando métodos colorimétricos. |

##### Muestra: Criterios de selección (Criterios de Inclusión, Exclusión y Retirada), reclutamiento

- - 1. **Muestra:** El tamaño muestral se ha calculado mediante el programa GRANMO con el modelo de proporciones observadas respecto de una de referencia. Para el cálculo se ha tenido en cuenta, que el “maximal respiration” es la clave para calcular los efectos del ejercicio físico (EF) en la función mitocondrial,


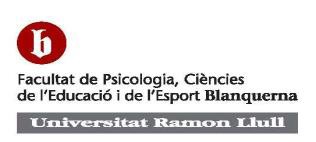


porque se fuerza la cadena respiratoria entera al máximo. Se ha realizado el cálculo aceptando un riesgo Alfa de 0,05 y un riesgo beta inferior al 0,2 en un contraste bilateral. Basado en datos preliminares, existe una diferencia de “maximal respiration” igual o superior a 0,263 unidades [11]. Se asume que la proporción en el grupo de referencia es del 0,019 en personas mayores de 50 años [5]. Siendo un estudio intervencional, el porcentaje de reposiciones necesarias se ha previsto que será del 20%. Por lo tanto se necesitan 8 sujetos por grupo, sumando un total de 24 pacientes para el estudio. Se reclutarán personas con EP a partir de los 50 y hasta los 70 años de edad, si cumple con los criterios de inclusión (pacientes con buen estado cognitivo) puntuación en el Montreal Cognitive Assessment (MoCA) de mayor o igual a 26, pacientes que hayan firmado el consentimiento informado, pacientes con capacidad de caminar independientemente durante 6 minutos, y pacientes con medicación estable (sin cambios durante el último mes). Se determinará la puntuación MDS-UPDRS-III (subescala motora) y el estadio de Hoehn y Yahr (hasta el estadio 3), se evaluará en la fase "ON". Se dividirá la muestra en tres grupos de pacientes estables después de la primera evaluación: un primer grupo (n=8) trabajará las capacidades físicas básicas, un segundo grupo (n=8) capacidades físicas básicas+estimulación de las vías sensomotoras específicas+ “dual task training” + actividad cognitiva; y un tercero será el Grupo Control (n=8). Una vez completada la primera evaluación, se realizará una aleatorización por bloque (ver Anexo aleatorización pág. 58) de la muestra en los tres grupos explicados. La aleatorización se realizará en base a los estadios de la EP (estadios I, II, III de Hoehn y Yahr), el sexo y la edad.

*Tabla 5:* ***Escala de estadios de la evolución de la enfermedad de Parkinson según Hoehn & Yahr***

| **ESTADÍOS** | **DESCRIPCIÓN** | **CLASIFICACIÓN** |
| --- | --- | --- |
| **Estadío I** | Afectación exclusivamente unilateral | Pacientes de diagnóstico reciente |
| **Estadío II** | Afectación bilateral, sin alteración del equilibrio | Pacientes de diagnóstico reciente |
| **Estadío III** | Afectación bilateral con alteración del equilibrio | Pacientes moderadamente afectados |
| **Estadío IV** | Aún capaz de caminar o de permanecer de pie sin ayuda | Pacientes con incapacidad grave Aumento del grado dedependencia |
| **Estadío V** | Permanecen en silla de ruedas o en cama si no tienen ayuda | Pacientes severamente afectados |

##### Criterios de inclusión:

-Pacientes con diagnóstico médico de EP idiopática, estadios de la escala de Hoehn y Yahr [29] del I-III .

-Pacientes que tengan un buen estado cognitivo puntuación en el Montreal Cognitive Assessment (MoCA) de mayor o igual a 26.

-Pacientes que hayan firmado el CI.

-Edad entre 50 y 70 años, y con capacidad de caminar independientemente durante seis minutos.

-Pacientes con medicación estable (que no hayan tenido cambios en la medicación durante el último mes).

##### Criterios de exclusión:

-Pacientes que presenten una patología distinta a la EP idiopática.

-Pacientes con deterioro cognitivo (puntuación en el MoCA <26 puntos).

-Pacientes con una enfermedad cardiovascular no controlada, alteración visual, o trastornos musculo-

esqueléticos recientes en las extremidades superiores o inferiores que pudieran interferir en el equilibrio y en la locomoción.

-Pacientes que en el momento del estudio estén realizando otro protocolo de ejercicio terapéutico.

-Pacientes que hayan sido intervenidos quirúrgicamente con el objetivo de influir en algún síntoma específico de la EP.

##### Criterios de retirada:

Se considera motivo de retirada la decisión voluntaria de los sujetos de retirarse del estudio en cualquier momento del mismo, así como cualquiera complicación que se pueda producir durante el tiempo que dure la intervención.

**6.2.3 Reclutamiento:** se realizará de manera progresiva y continuada, en dependencia de la velocidad de incorporación de los sujetos. Estará a cargo del IP Dr. Jorge Hernández Vara, Médico Adjunto del Servicio de Neurología (Área de Trastornos del Movimiento) del HUVH, en coordinación con los centros de colaboración y con la participación del doctorando. Para asegurar el reclutamiento mínimo de 24 pacientes según lo establecido en el proyecto, los investigadores del VHIR utilizarán sus contactos con la Asociación Catalana de Parkinson y con otras Unidades de Trastornos del Movimiento de los principales hospitales del estado.

- Implementar 2 líneas de actividad física, con 3 clases por semana durante 4 meses, una para cada una de los grupos que realizarán AF.

##### Procedimiento:

En primer lugar, se entrevistará a cada uno de los participantes, los cuales ya habrán leído y firmado previamente el consentimiento informado. En la misma entrevista se consultará la historia clínica mediante una anamnesis completa, obteniendo así datos sobre su actual situación de salud y también de la cronología de su patología en particular. En la entrevista se elaborará la historia clínica, y exploración neurológica para confirmar criterios de inclusión-exclusión (pares craneales, sensibilidad, fuerza motora, reflejos). Antes de la intervención, se llevarán a cabo 2 sesiones de familiarización; los participantes podrán conocer al personal que trabajará con ellos (auxiliares e investigadores), los lugares donde serán evaluados y donde se realizará la intervención; también podrán familiarizarse con los equipos de laboratorio, el material a utilizar en la práctica de los ejercicios, y las diferentes técnicas de evaluación.

##### La evaluación inicial incluirá: (a realizar por los 2 grupos de intervención + el grupo control)

1. Comprobación del estado de salud general, donde se analizará la aptitud de cada uno de los participantes, para realizar AF.
2. Una batería de pruebas de campo, (descripta en la Evaluación funcional y Motora-Diagrama General de flujo de la investigación (pág, 13).
3. Una Anamnesis y exploración neurológica (pares craneales, sensibilidad, fuerza motora, reflejos).
4. Tests ortopédicos y neurodinámicos de extremidades superiores e inferiores (EESS-EEII).
5. Tests psicológicos destinados a valorar aspectos cognitivos, como el Montreal Cognitive Assessment (MoCA), la escala autoaplicada para la evaluación de la depresión (Inventario de Depresión de Beck; IDB), con el fin de excluir aquellos sujetos que manifestaran un trastorno depresivo (puntuación de 10-13/15), puntuación final se valorará con la Neuropsicóloga Catalina Pons Marquès, y como alternativa Self-Rating Depresion Scale (SDS) de Zung. [30, 31, 32]

-También el Parkinson's Disease Cognitive Rating Scale (PD-CRS) que ha sido creado por neurólogos del Hospital Sant Pau.

-Clock Drawing Test (CDT), es interesante por la diferenciación que realiza entre las funciones cortical y subcortical.

-Trail Making Test A (TMT-A) and B (TMT-B), estos tests se verán una vez se concretice la muestra, dependiendo del grado de temblor de los pacientes.

1. Tests de calidad de vida, humor, y sueño.

Esta evaluación se repetirá tres veces: al inicio y al final de la intervención; y a los 4 meses después de finalizar la intervención (8 meses de la evaluación inicial). Se realizarán en el campus FCS-Blanquerna de la Universitat Ramon Llull (URL), y/o centro de colaboración. La logística será decidida teniendo en cuenta la seguridad, la mayor comodidad y el menor desplazamiento por parte de los pacientes.

- 1. **Intervención:** los 2 programas diseñados tendrán una duración de 4 meses. Las intervenciones a realizar serán grupales, 8 pacientes por grupo (2 grupos de intervención + 1 grupo control ), de 60 minutos de duración, con una frecuencia de 3 veces por semana. Dichos programas de intervención se estructurarán en 4 meso-ciclos de 4 semanas cada uno. Durante las primeras 2 semanas se mantendrán las cargas de trabajo, para favorecer al periodo de adaptación, de igual forma en los programas que también requieren además de una adaptación a la carga, también una adaptación a las tareas de ejecución motora compleja.

Con un aumento progresivo de las cargas.

Los participantes del grupo control continuarán con su práctica diaria habitual y serán entrevistados una vez por semana por los investigadores para comprobar que sus rutinas no se han alterado. Este GC recibirá 4 meses de AF del programa que haya obtenido los mejores resultados, en cuanto a sintomatología, y calidad de vida, después de la última valoración (8 meses).

##### Variables

*Tabla 6: Variables de estudio*

**Variables directas**

- - - Respirometría
    - Fuerza muscular de MMSS yMMII
    - Equilibrio
    - Marcha
    - Resistencia

**Variables indirectas**

- - - Función Mitocondrial
    - Función motora
    - Calidad de vida
    - Aspectos cognitivos
    - Sueño
    - Humor

##### Recogida de datos

El investigador preparará adecuadamente el área de recolección de datos, minimizando los Potenciales riesgos, cumpliendo así con las medidas sanitarias tomadas en base al COVID 19.

En la Fase España se recolectarán las biopsias de fibroblastos de piel para ser cultivadas y expandidas (aumentar el número de células) hasta obtener 3 millones de células (siguiendo recomendaciones de la Dra. Susana P. Pereira), para luego ser transportadas a Portugal. La participación de los pacientes en esta investigación, es un gran aporte en la búsqueda de aportes significativos en el tratamiento y abordaje de la progresión en la EP. Y también su compromiso de participación en este estudio, no solo es un bien para su persona, también lo es para la sociedad (como lo es una donación de sangre); y también es un bien para el avance de la ciencia como portadora de luz a los caminos de los procesos neurodegenerativos, para mejorar la calidad de vida de los pacientes. Demás datos de las evaluaciones recogidas en España se irán almacenando en los dispositivos informáticos del equipo de investigadores, protegido bajo clave.

Con las células en Portugal, se realizarán análisis de Seahorse, se buscarán mediciones autofágicas y cambios posibles en la bioenergética celular. El resto de la información se encuentra en el apartado de instrumento.

- 1. **Análisis de datos:** se llevarán a cabo con el programa estadístico SPSS v.20 (IBM SPSS Statistics). Se concretará con el Tutor y Director de Tesis los siguientes Análisis:

-Análisis descriptivo: Se analizarán todas las variables de la muestra mediante frecuencias relativas y absolutas, así como frecuencias de dispersión y de tendencia central.

-Análisis de medidas repetidas: Modelo de efectos mixtos para medidas repetidas, muestra homogénea o muestra no homogénea. Permitirán analizar los posibles cambios entre los diferentes momentos de la intervención y evaluar qué tipos de intervención aporta más beneficios físicos, ya sea en los factores de la función mitocondrial, fisiológicos, la calidad de vida o los aspectos cognitivos y emocionales.

-La rho de Spearman y la r de Pearson para evaluar la asociación entre dos variables que tienen categorías ordinales. La normalidad de la distribución de los resultados de cada grupo se evaluará mediante la prueba de normalidad de Shapiro-Wilk. α = 0.05 considerado el umbral para pasar la prueba de normalidad. Si los datos presentan una distribución normal, se realizará una prueba t pareada paramétrica. De lo contrario, se utilizará la prueba de Mann-Whitney. Los valores de prueba estadística con p <0,05 se considerarán diferencias estadísticamente significativas.

###### 7--Los aspectos éticos

Esta investigación se plantea siguiendo los principios de la declaración de Helsinki y la legislación nacional e internacional aplicable en esta materia, así como la Carta de los Derechos Fundamentales de la Unión Europea y el Convenio Europeo de Derechos Humanos. Nos hemos basado en la tradición Anglo-Americana de los Principios Éticos de la Investigación. En todo momento de la investigación, se mantendrá presente la intimidad y privacidad física de las personas, como así también la confidencialidad de los datos solicitados y establecidos propios de la investigación. Se tomarán imágenes exclusivamente para fines del presente estudio, y se pixelará la cara para que los sujetos no sean identificables. las imágenes obtenidas nunca irán acompañadas de datos o informaciones que puedan revelar su identidad a terceros.

La investigación que hemos planteado siguen las normas que hacen referencia al Reglamento General Europeo de Protección de Datos (RGPD), Reglamento (UE) 2016/679; y privacidad y confidencialidad de datos (LOPDGD) Ley Orgánica (3/2018 de 5 de diciembre) de Protección de Datos Personales y garantía de los derechos Digitales.

Consentimientos informados e información del paciente se adjuntan en anexos, como así también el apartado específico de Principios básicos de la Ética aplicada a la Investigación (Reflexión Ética sobre esta investigación).

###### 8--Aplicabilidad, Las limitaciones y líneas futuras

**Aplicabilidad:** Citando parte de una de las conclusiones del estudio de Ambrosi [10] “la investigación de los mecanismos patogénicos en las células periféricas, como los fibroblastos derivados de pacientes con EP esporádica y controles emparejados por edad/sexo, podría generar una comprensión más profunda de los déficits que afectan a las neuronas dopaminérgicas y, posiblemente, nuevas herramientas aplicables a la práctica clínica.” Es así que, en medio de esta conclusión, nuestra investigación pretende incorporar/aportar una nueva variable, que será analizada por medio de los fibroblastos; para obtener nuevas conclusiones/resultados de cómo influye los distintos programas de AF en pacientes con EP.

**Las limitaciones**: Algunas limitaciones surgen de preguntas que nos hemos hecho, y cuyas respuestas aún no se han descripto en la literatura científica y/o aún no se ha investigado. Tales preguntas como ¿Cuánto es el tiempo mínimo de AF para poder observar cambios en los fibroblastos? ¿qué tipo de AF produce cambios en las mitocondrias de los fibroblastos de la piel? Actualmente se desconocen las respuestas, ya que por lo general los tejidos que se han tenido en cuenta para este tipo de análisis en humanos, son principalmente el músculo esquelético o grasa visceral, el nuevo enfoque de los fibroblastos está en continuo crecimiento.

Una limitación potencial que puede estar asociada a los fibroblastos, sería que no muestren ninguna alteración fenotípica asociada con el ejercicio, ya que esta condición predispone a una mayor capacidad mitocondrial pero no garantiza que esta diferencia sea notoria en un estado basal (Susana P. Pereira). En ciencia las limitaciones de hoy, pueden ser las fronteras que cruzaremos mañana. Por lo tanto, planteamos superar esta limitación desafiando a las mitocondrias de los fibroblastos de la piel, forzando a las células a depender del metabolismo de OXPHOS (Sistema de fosforilación oxidativa) para sobrevivir, podemos hacerlo usando el medio OXPHOS [10].

Tampoco debemos olvidar que estamos en medio de una pandemia (COVID-19) y es muy difícil el acceso a los centros sanitarios, hospitales, clínicas. Ya hemos comenzado la fase protocolar de búsqueda y contactos con profesionales en centros clínicos y hospitales (VHIR). Actualmente nos encontramos evaluando las distintas opciones de estrategias de sinergias posibles con otros centros.

Y otras limitaciones sería el “dropout” o pérdidas, pero ya se ha tenido en cuenta en el cálculo muestral.

**-Líneas futuras:** Están relacionadas con la aplicabilidad a otras EN.

###### 9--Plan de trabajo y Cronograma

**2020 2021 2022 2023 2024**

Plan de inve stiga ción

| **7** | **8** | **9** | **1** | **1** | **1** | **1** | **2** | **3** | **4** | **5** | **6** | **7** | **8** | **9** | **1** | **1** | **1** | **1** | **2** | **3** | **4** | **5** | **6** | **7** | **8** | **9** | **1** | **1** | **1** | **1** | **2** | **3** | **4** | **5** | **6** | **7** | **8** | **9** | **1** | **1** | **1** | **1** | **2** | **3** | **4** | **5** | **6** | **7** | **8** | **9** | **1** | **1** | **1** |
| --- | --- | --- | --- | --- | --- | --- | --- | --- | --- | --- | --- | --- | --- | --- | --- | --- | --- | --- | --- | --- | --- | --- | --- | --- | --- | --- | --- | --- | --- | --- | --- | --- | --- | --- | --- | --- | --- | --- | --- | --- | --- | --- | --- | --- | --- | --- | --- | --- | --- | --- | --- | --- | --- |
|  |  |  | **0** | **1** | **2** |  |  |  |  |  |  |  |  |  | **0** | **1** | **2** |  |  |  |  |  |  |  |  |  | **0** | **1** | **2** |  |  |  |  |  |  |  |  |  | **0** | **1** | **2** |  |  |  |  |  |  |  |  |  | **0** | **1** | **2** |

Revi sión Siste máti ca

FASE 1 ESPA ÑA

FASE 2 POR TUG AL

Definición de tema de estudio

Elaboración de marco referencial Desenvolvimiento del plan de investigación Presentación del plan de investigación

Revisión de la literatura científica Elaboración de la revisión sistemática Presentación del articulo a una revista científica

Definición de los programa de AF Familiarización de los investigadores con el programa Reclutamiento de la muestra Aleatorización del estudio

Evaluación inicial Intervención Post test

Extracción Fibroblastos Amplificación y congelación Fibroblastos

Familiarización del doctorando con los investigador del CNC- UC

Recepción Muestras 1

2

3

Recoge y análisis de los datos

Tesis Objetivos conclusiones Métodos y resultados

Discusión

20 Conclusiones

Versión 4, 05/12/2022

*- Diagrama de diseño y Plan de publicación general derivada de la tesis.*


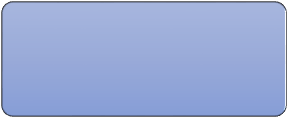

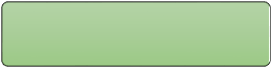


**Objetivo principal**

**Objetivo**

**secundario**

|  |  | **Abstract** | |
| --- | --- | --- | --- |
|  |  |  |  |
|  |  |  | **Revisión**  **sistemática** |
|  | | |  |

**Publicación de la Tesis**

Estudio Clínico de intervención no farmacológico, para mejorar diferentes biomarcadores de salud en personas con EP. Con el objetivo de valorar la función mitocondrial, utilizando fibroblastos de piel como biomarcador, en pacientes con EP: efectos de 2 programas de AF sobre la función motora, la calidad de vida, el sueño, aspectos cognitivos y el humor.”

**2020**

**2021**

**2021**

**2022**

**2021**

**2022**

**-Formación Transversal (F.T.)**

**-Definir la Co-Tutela de la Tesis especializado en Biología Celular-Enfermedades Neurodegenerativas-Biomedicina**

**-Búsqueda Bibliográfica. Estudio de la Bibliografía.**

**-Elaboración del Plan de Investigación (E.P.I)**

**-Presentación del Plan de Investigación. (P.P.I.)**

**-Defensa del Plan de Investigación.**

**-Formación Específica (F.E)**

**-Actividades de Formación e Investigación, preparar Protocolo, pasar comité ético, coordinación del estudio.**

**-Financiación**

**-Presentación de Póster o Abstract en un congreso científico. Abstract presentado (2021)**

**-Financiación**

**-Reclutamiento de los participantes, centros, fundaciones, hospitales. Entrevistas-Consentimiento Informado**

**-Evaluación Inicial**

**-Siguientes evaluaciones**

**2022 -Intervenciones**

**2023 -Intervención Grupo Control (Finalizado los programas de AF, el GC recibirá 4 meses de AF)**

**2023**

**2024**

**-Artículo Científico**

**-Análisis de datos**

**-Estancias en Centros Internacionales (E.C.I.)**

**-Conclusiones**

**-Depósito de la Tesis**

###### 10--Recursos necesarios

- 1. **Recursos necesarios**

Los materiales necesarios para llevar a cabo este estudio son:

**-Información sobre CNC** (ahora CIBB; cibb.uc.pt): Es la segunda unidad portuguesa de I + D en el ámbito de las ciencias de la vida y la salud, cuenta con 3 sitios en los campus de la Universidad de Coimbra.

Principalmente dedicada a la investigación, ciencias de la salud, y por esto está ubicada muy próxima a los Hospitales Universitarios (CHUC), donde potencia la investigación traslacional, y el sitio UC-Biotech en el

parque biotecnológico BIOCANT (sede del 40% de las empresas biotecnológicas en Portugal). Ofrece a

21

los investigadores un entorno único y excelentes infraestructuras de investigación (varias de las cuales integran los ERIC Euro-Bioimaging, Instruct o EATRIS). En particular, CIBB cuenta con el Analizador Seahorse XFe96, para medidas de metabolismo, las unidades de microscopía para análisis celular y plataformas que incluyen un analizador celular GE In Cell Analyzer 2200, el sistema Fluidigm Biomark HD para ensayos automatizados tanto a nivel celular (basado en microscopía) como genético (PCR).

-**El Instituto de Investigación Vall d'Hebron (VHIR)** cuenta con más de 60 grupos de investigación y más de 1.400 personas dedicadas a la investigación de una amplia gama de enfermedades y mejora de la atención, que se aplican a la práctica clínica del hospital. El Grupo de Enfermedades Neurodegenerativas fue creado en el año 2006 por el profesor de investigación ICREA Dr. Miquel Vila. Actualmente, el grupo tiene 4 investigadores principales, a parte del jefe de grupo, y está formado por 25 investigadores incluyendo estudiantes de doctorado, técnicos de laboratorio e investigadores postdoctorales. El grupo forma parte de la red española de grupos de investigación en enfermedades neurodegenerativas (CIBERNED) y es reconocido como grupo consolidado por la Agencia de Gestió d’Ajuts Universitaris i de Recerca (AGAUR) de la Generalitat de Catalunya. El grupo trabaja en diferentes líneas de investigación punteras en el campo de la Enfermedad de Parkinson como son el papel de la neuromelanina y la alpha-synucleina, la disfunción autofàgica y lisosomal, y las alteraciones del sistema inmune y de la microbiota intestinal. Además del excelente entorno científico, el laboratorio está equipado con todo el material necesario y las instalaciones necesarias para realizar las actividades propuestas en este proyecto. Actualmente, el grupo de enfermedades Neurodegenerativas del VHIR cuenta con más de 11 proyectos activos a nivel nacional e internacional financiado por organismos públicos o entidades privadas, incluyendo el proyecto “The Vall d’Hebron Iniciative for Parkinson associated to GBA (VHIP-GBA): from biospecimen collection to translational studies and therapies” financiado por el Instituto de Salud Carlos III. Un resumen de las diferentes líneas de investigación, así como las principales publicaciones del grupo se pueden observar en el siguiente enlace: <https://vilalab.org/?years=2015_present>

-**Contrato (Doctorando) Pre-doctoral** de personal investigador en formación (PIF)-Blanquerna.

-Formación continua, Membresía en International Parkinson and Movement Disorder Society (MDS)

-FCS: Sala

-Otros recursos necesarios de material de AF, para llevar a cabo la intervención, disponibles en FPCEE.

##### Distribución del presupuesto

-El procedimiento de biopsia de piel tiene un presupuesto entre 190 y 220 euros por paciente y exploración. Antonia Campolongo Perillo-Enfermera de la Unidad de Trastornos del Movimiento. Hospital Sant Pau Telefono de contacto: +34683524471 EXT interna:30127

-Derivación, expansión y criopreservación de fibroblastos. (Criopreservados en nitrógeno líquido, o algún otro medio como dispositivo Mrs frosty para lograr la congelación sin la formación de cristales, u otro medio que pueda garantizar la viabilidad de las células).

22

- Aislar los fibroblastos, cultivarlos para expandirlos (aumentar el número de células) hasta obtener 3 millones de células (por muestra recogida).

*-Tabla 7: Presupuesto Fibroblastos Fase España-IBIDELL*

| **TARIFA EXTERNA PRIVADA** | **Para 1 muestra procesada independientemente** | **Para 42 muestras en dos tandas de 21 muestras** | **Para 42 muestras a la vez** |
| --- | --- | --- | --- |
| Derivación/expansión/criopreservación HDF (para 1 muestra) | 767,901 | 32251,827 | 32251,82693 |
| Test de Micoplasma | 19,718 | 828,172 | 828,172 |
| Uso sala de cultivos (45 días) | 2639,364907 | 5278,730 | 2639,364907 |
| **COSTE TOTAL PARA 42 MUESTRAS** | **€ 143933,325** | **€ 38358,728** | **€ 35719,364** |
|  |  |  |  |

Teresa Zomeño, PhD.-Responsable de la Plataforma de Cultius Avançats de Cèl·lules i Teixits Serveis Cientificotècnics-Tel: +34 93 316 03 17 [actc@idibell.cat](mailto:actc@idibell.cat) - SPAIN [www.idibell.cat](http://www.idibell.cat/) Institut d'investigació Biomédica Bellvitge-Hospital Duran i Reynals

Gran Via de L’Hospitalet, 199-08908 Hospitalet de Llobregat Barcelona

##### -A este primer presupuesto presentado, al haber aceptado el VHIR ser nuestro centro colaborador y ser parte de nuestra investigación, asume el costo de la Biopsias y la primera fase del cultivo de los Fibroblastos.

- 1. **POSIBLES FUENTES DE FINANCIACIÓN**

**-Fuentes públicas:** se pedirán proyectos competitivos en instituciones públicas como proyecto de Retos que favorece a la Cooperación internacional en investigación, PERIS, otros.

**-Fuentes privadas:** se realizará una búsqueda de posibles fuentes privadas que en la actualidad financian proyectos semejantes.

##### -Financiaciones y ayudas presentadas:

| **Descripción y objeto** | **Entidad financiadora** | **Dura ción** | **TOTAL DE AYUDA SOLICITADA** |
| --- | --- | --- | --- |
| **Convocatoria para la concesión**  **de Becas para la financiación de Proyectos de Investigación** | Fundación Eugenio Rodríguez Pascual (Solicitud presentada en Julio de 2021) | 1  año | 25.000 euros |
| **Ayuda a la actividad de investigación del personal docente e investigador de la URL** | Universidad Ramon Llull | 1  año | 20.000 euros |
| **Convocatoria Premio Especial a la Innovación en el Sector Sociosanitario.** | Fundación DomusVi | 1  año | 20.000 euros |

**-Financiaciones y ayudas conseguidas:** Ayuda a la actividad de investigación del personal docente e investigador de la URL (Ref: 2021-URL-Proj-004)

###### 11--Investigadores, tutorías y opinión experta de otros profesionales

Este trabajo se ha nutrido de la experiencia y opinión experta de los Co-directores de tesis, y también de los siguientes profesionales:

-Tutoría y opinión experta (“entrevista a expertos”) del Dr Jaime Kulisevsky (Hospital Sant Pau) Director “Fundació Institut de Recerca de l'Hospital de la Santa Creu i Sant Pau”.

-Enfermera del grupo del Dr Jaime Kulisevsky-Antonia Campolongo Perillo-Enfermera de la Unidad de Trastornos del Movimiento. Hospital Sant Pau. 23

-Neuropsicologa Catalina Pons Marquès, Psicóloga General Sanitaria, Máster en Neuropsicología.

-Dra. María Giné.

-Investigadores del Grupo de Enfermedades Neurodegenerativas del VHIR. ***12-- Plan de Difusión, Comunicación, Presentación de Abstract, Premios.* Primeros pasos en la difusión de la investigación:**

**-Redes Sociales:** Hemos creado una cuenta en Instagram y en la actualidad ya contamos con más de 3300 seguidores.

**-También hemos creado un video de 60´´** con los principales objetivos de la investigación, y en el cual difundimos el equipo de trabajo y las principales instituciones de esta colaboración internacional entre España y Portugal (Blanquerna-URL, CNC-Portugal).

**Los resultados**: obtenidos en este proyecto se difundirán como publicaciones completas en revistas científicas originales de alto impacto (Q1 / top 10%) revisadas por pares (p. Ej., Metabolism: Clinical and Experimental, IF = 6.51, Top 6%, and Journal of Clinical Investigación). Todas las publicaciones estarán en un esquema de acceso abierto. Los resultados se presentarán en forma de charlas orales y carteles en diferentes encuentros nacionales e internacionales como European Society for Clinical Investigation.

**Formación:** Este proyecto contribuirá a la formación de recursos humanos, pudiendo resultar en varias tesis de maestría y doctorado. También tengo la intención de organizar actividades de concienciación científica centradas en mostrar que la intervención con ejercicio puede mejorar el fenotipo de la EP y ralentizar la progresión.

**Comunicación:** Este campo es una nueva y poderosa área de acción en las ciencias de la vida, y mi objetivo es contribuir a reducir los efectos nocivos de la EP. Creo que el proyecto contribuye a las metas de la ONU al asesorar intervenciones sobre estilos de vida que pueden corregir y / o prevenir el progreso de la enfermedad para asegurar una vida sana y promover el bienestar de todas las edades.

Apoyados por la gran experiencia del CNC en comunicación científica, participaremos en actividades científicas dirigidas a diferentes públicos y grupos de interés, que van desde jornadas de laboratorio abierto, actividades en centros científicos y museos, actividad en escuelas para diferentes grupos destinatarios, noticias en medios (radio, televisión, periódicos), noticias periódicas en las redes sociales (incluidos LinkedIn y Facebook), producción de materiales impresos, incluidos folletos, y grupos especializados de profesionales de la salud.

Creemos que el proyecto se ajusta perfectamente a la misión de la institución anfitriona, lo que resulta en una contribución significativa para los desafíos sociales críticos. Estos resultados constituirán un importante cambio de paradigma que llevará a varios profesionales sanitarios a plantearse la alteración de las guías clínicas durante la EP para prevenir la progresión de la enfermedad.

Los resultados y conclusiones obtenidos en este estudio se intentará divulgarlos presentándolos tanto a revistas científicas, como a congresos y jornadas que tengan relación con la AF, las EN, la EP, las Ciencias de la Salud, Biomedicina, Neurología, Salud Mitocondrial y ejercicio, las ciencias de la Actividad Física y del Deporte, Neurociencias, entre otras.

##### Presentación de Abstract en el Congreso CINPSUS- I (International Interdisciplinary Congress on Public Health Policies).

| **Autores** | **Autor que realizó la presentación** | **Título** | **Congreso** | **Entidad organizadora y fechas** | **Indicios de calidad** |
| --- | --- | --- | --- | --- | --- |
| **-Juan Carlos** | Juan Carlos | Abstract: EVALUATING THE | CINPSUS - I | Realizado por el Grupo | Código del |
| **Magaña [1]** | Magaña | EFFICACY OF TWO PHYSICAL | International | de Investigación | trabajo |
| **-Catalina Pons** |  | ACTIVITY PROGRAMS TO | Interdisciplin | Interdisciplinar en | aprobado |
| **Marqués [2]** |  | PREVENT PARKINSON´S | ary Congress | Salud, Educación y | por la |
| **-Maria Giné-** |  | DISEASE PROGRESSION | on Public | Educación Física- | comisión |
| **Garriga [3]** |  | USING MITOCHONDRIAL | Health | GIPEEF-Universidad | científica |
| **-Susana P.** |  | FUNCTION FROM PATIENTS | Policies | Federal del Valle de San | del |
| **Pereira [4]** |  | SKIN FIBROBLASTS AS |  | Francisco | congreso: |
| **-Joel Montané** |  | BIOMARKER |  | 18/06/2021 | 00535 |
| **[5]** |  |  |  | 19/06/2021 |  |

El congreso CINPSUS fue realmente un gran éxito, en el que participaron más de 3.100 postulantes de 14 nacionalidades diferentes y se recibieron más de 1.800 trabajos en portugués, español e inglés. Al final, se presentaron 392 trabajos, de los cuales se han premiado a los mejores 30 trabajos. **El nuestro ha sido uno de los 30 mejores trabajos.**

-También pretendemos difundir, asistir y/o participar de congresos como el European Society for Clinical Investigation meeting, Congreso Nacional de Ciencias de la Actividad Física y del Deporte (CAFE), Congreso Nacional de Estudiantes ciencias de la salud, de CAFE, Congresos Europeo de EN, EP, Congreso, Internacional de la WCPT (Word Confederation for Physical Therapy), Participar en las Jornadas de, Parkinson que organiza la Federación Española de Parkinson, International Neuropsychological Society World Congress, International Congress on Non-Motor Dysfunctions in Parkinson’s Disease and Related Disorders.

##### Revistas

- Se tendrán en cuenta diferentes tipos de revistas de Q1, según los resultados.
- Physiotherapy, Physical Therapy, Journal of Physiotherapy, Disability and Rehabilitation, International Journal of Neuroscience, Revistas de Educación Física, otras.

###### 13--Referencias Bibliográficas:

[1] OMS | Los trastornos neurológicos afectan a millones de personas en todo el mundo: informe de la OMS [Internet]. Disponible en: <http://www.who.int/mediacentre/news/releases/2007/pr04/es>

[2] OMS | ¿Qué son los trastornos neurológicos? [Internet]. Disponible en:<https://www.who.int/features/qa/55/es/>

[3] Sánchez, C. S. (2006). Impacto Sociosanitario De Las Enfermedades Neurológicas En España. Fundación Española de Enfermedades Neurológicas (FEEN). Retrieved from<http://www.fundaciondelcerebro.es/docs/imp_sociosanitario_enf_neuro_es.pdf>

[4] Domingo, E. P., Sierra, M. G., Valero, M. M., & Castiñeira, M. P.-O. (2015). El Libro Blanco del párkinson en España - Aproximación, análisis y propuesta de futuro. Madrid: Real Patronato sobre Discapacidad (Ministerio de Sanidad, Servicios Sociales e Igualdad) y Federación Española de Párkinson.

Retrieved from <http://www.fedesparkinson.org/libro_blanco.pdf>

[5] Garcés, Mario y Crespo Puras, María del Carmen y Finkel Morgenstern, Lucila y Arroyo Menéndez, Millán (2016) Estudio sobre las enfermedades neurodegenerativas en España y su impacto económico y social.

[6] Erbach, G. (2013). Neurodegenerative diseases in the workplace. Library of the European Parliament. [7] Tu PH, Galvin JE, Baba M, et al. Glial cytoplasmic inclusions in white matter oligodendrocytes of multiple system atrophy brains contain insoluble alpha-synuclein. Ann Neurol 1998;44:415-422.

[8] Martínez-Fernández., R., Gasca-Salas C., C., Sánchez-Ferro, Á., & Ángel Obeso, J. (2016). ACTUALIZACIÓN EN LA ENFERMEDAD DE PARKINSON. Revista Médica Clínica Las Condes, 27(3), 363–379. https://doi.org/https://doi.org/10.1016/j.rmclc.2016.06.010

[9] Carvalho, A., Barbirato, D., Araujo, N., Martins, J. V., Cavalcanti, J. L., Santos, T. M., Coutinho, E. S., Laks, J., & Deslandes, A. C. (2015). Comparison of strength training, aerobic training, and additional physical therapy as supplementary treatments for Parkinson's disease: pilot study. Clinical interventions in aging, 10, 183–[191. https://doi.org/10.2147/CIA.S68779](https://doi.org/10.2147/CIA.S68779)

[10] Ambrosi, G., Ghezzi, C., Sepe, S., Milanese, C., Payan-Gomez, C., Bombardieri, C. R., Armentero, M. T., Zangaglia, R., Pacchetti, C., Mastroberardino, P. G., & Blandini, F. (2014). Bioenergetic and proteolytic defects in fibroblasts from patients with sporadic Parkinson’s disease. Biochimica et Biophysica Acta -

Molecular Basis of Disease, 1842(9). <https://doi.org/10.1016/j.bbadis.2014.05.008>

[11] Deus, C. M., Pereira, S. P., Cunha-Oliveira, T., Pereira, F. B., Raimundo, N., & Oliveira, P. J. (2020). Mitochondrial remodeling in human skin fibroblasts from sporadic male Parkinson’s disease patients uncovers metabolic and mitochondrial bioenergetic defects. Biochimica et Biophysica Acta - Molecular Basis of Disease, 1866(3). <https://doi.org/10.1016/j.bbadis.2019.165615>

[12] Brooks, G. A., Brown, M. A., Butz, C. E., Sicurello, J. P., & Dubouchaud, H. (1999). Cardiac and skeletal muscle mitochondria have a monocarboxylate transporter MCT1. Journal of applied physiology (Bethesda, Md. : 1985), 87(5), 1713–1718. <https://doi.org/10.1152/jappl.1999.87.5.1713>

[13] Acin-Perez, R., Benador, I. Y., Petcherski, A., Veliova, M., Benavides, G. A., Lagarrigue, S., Caudal, A., Vergnes, L., Murphy, A. N., Karamanlidis, G., Tian, R., Reue, K., Wanagat, J., Sacks, H., Amati, F.,

Darley-Usmar, V. M., Liesa, M., Divakaruni, A. S., Stiles, L., & Shirihai, O. S. (2020). A novel approach to measure mitochondrial respiration in frozen biological samples. The EMBO Journal, 39(13), e104073. https://doi.org/https://doi.org/10.15252/embj.2019104073

[14] Carter, H. N., Chen, C. C., & Hood, D. A. (2015). Mitochondria, muscle health, and exercise with advancing age. Physiology (Bethesda, Md.), 30(3), 208–[223. https://doi.org/10.1152/physiol.00039.2014](https://doi.org/10.1152/physiol.00039.2014) [15] Sen C. K. (1995). Oxidants and antioxidants in exercise. Journal of applied physiology (Bethesda, Md. : 1985), 79(3), 675–686. <https://doi.org/10.1152/jappl.1995.79.3.675>

[16] Hoppeler, H., Howald, H., Conley, K., Lindstedt, S. L., Claassen, H., Vock, P., & Weibel, E. R. (1985). Endurance training in humans: aerobic capacity and structure of skeletal muscle. Journal of applied physiology (Bethesda, Md. : 1985), 59(2), 320–327. <https://doi.org/10.1152/jappl.1985.59.2.320>

[17] Barrios, L., & López, M. (2011). Aportes del ejercicio físico a la actividad cerebral. Lecturas: Educación Física y Deportes (Revista Digital) (160), N/A.

[18] La actividad física como factor benéfico a nivel neurológico Vol. 3, núm. 1., (2019) Juan Antonio Vera Hinojosa; Karla Lissette Flores Flores; Natalia del Carmen Alvarado, Linda Beatriz Dávila Solórzano.

[19] Severinsen, M., & Pedersen, B. K. (2020). Muscle-Organ Crosstalk: The Emerging Roles of Myokines.

Endocrine reviews, 41(4), 594–609. <https://doi.org/10.1210/endrev/bnaa016>

[20] Soria, Bernat. «Biomedical research in Spain: the patient’s point of view». Contributions to science, [en línia], 2009, p. 91-94, https[://www](http://www.raco.cat/index.php/Contributions/article/view/188419).[raco.cat/index.php/Contributions/article/view/188419](http://www.raco.cat/index.php/Contributions/article/view/188419) [Consulta: 29-04-

2021].

[21] Bloomer, R. J., Schilling, B. K., Karlage, R. E., Ledoux, M. S., Pfeiffer, R. F., & Callegari, J. (2008). Effect of resistance training on blood oxidative stress in Parkinson disease. Medicine and Science in Sports and Exercise, 40(8), 1385–1389.

[22] G. Auburger, M. Klinkenberg, J. Drost, K. Marcus, B.Morales-Gordo, W.S. Kunz, et al., Primary skin fibroblasts as amodel of Parkinson's disease,Mol. Neurobiol. 46 (2012) 20–27.

[23] C. Mytilineou, P.Werner, S. Molinari, A. Di Rocco, G. Cohen, M.D. Yahr, Impaired oxidative decarboxylation of pyruvate in fibroblasts from patients with Parkinson's disease, J. Neural Transm. Park. Dis. Dement. Sect. 8 (1994) 223–228.

[24] Milanese, C., Payán-Gómez, C., Galvani, M., Molano González, N., Tresini, M., Nait Abdellah, S., van Roon-Mom, W. M. C., Figini, S., Marinus, J., van Hilten, J. J., & Mastroberardino, P. G. (2019). Peripheral mitochondrial function correlates with clinical severity in idiopathic Parkinson’s disease. Movement Disorders : Official Journal of the Movement Disorder Society, 34(8), 1192–1202.<https://doi.org/10.1002/mds.27723>

[25] Miyai, I., Fujimoto, Y., Ueda, Y., Yamamoto, H., Nozaki, S., Saito, T., & Kang, J. (2000). Treadmill training with body weight support: its effect on Parkinson's disease. Archives of physical medicine and rehabilitation, 81(7), 849–852. <https://doi.org/10.1053/apmr.2000.4439>

[26] Steffen, T., Petersen, C., & Dvorak, L. (2012). Community-based exercise and wellness program for people diagnosed with Parkinson disease: experiences from a 10-month trial. Journal of geriatric physical therapy (2001), 35(4), 173–180. <https://doi.org/10.1519/JPT.0b013e31824a1c9d>

[27] Gobbi, L. T., Oliveira-Ferreira, M. D., Caetano, M. J., Lirani-Silva, E., Barbieri, F. A., Stella, F., & Gobbi, S. (2009). Exercise programs improve mobility and balance in

people with Parkinson's disease. Parkinsonism & related disorders, 15 Suppl 3, S49–S52.<https://doi.org/10.1016/S1353-8020(09)70780-1>

[28] Tanaka, K., Quadros, A. C., Jr, Santos, R. F., Stella, F., Gobbi, L. T., & Gobbi, S. (2009). Benefits of physical exercise on executive functions in older people with Parkinson's disease. Brain and cognition, 69(2), 435–[441. https://doi.org/10.1016/j.bandc.2008.09.008](https://doi.org/10.1016/j.bandc.2008.09.008)

[29] Hoehn, M., & Yahr, M. (2001). Hoehn MM, Yahr MD. Parkinsonism: onset, progression and mortality.

Neurology 17: 427-442. Neurology, 57, S11-26. <https://doi.org/10.1212/WNL.17.5.427>

[30] Conde, V., Esteban, T. y Useros, E. (1976). Revisión crítica de la adaptación castellana del Cuestionario de Beck. Revista de Psicología General y Aplicada, 31, 469-497.

[31] Conde, V., Escriba, P. e Izquierdo, J.A. (1970). Evaluación estadística y adaptación castellana de la Escala Autoaplicada para la Depresión (SDS) de Zung. Publicaciones de la Sociedad Española de Psicología, 30, 867-880.

[32] Conde, V., y Useros, E. (1974). El inventario para la medida de la depresión de Beck. Revista de Psiquiatría y Psicología Médica de Europa y América Latina, 12, 153-167

***Referencias de Anexos (En el documento “Principios básicos de la Ética aplicada a la Investigación”):*** [33] Speelman, A. D., van de Warrenburg, B. P., van Nimwegen, M., Petzinger, G. M., Munneke, M., & Bloem, B. R. (2011). How might physical activity benefit patients with Parkinson disease?. Nature reviews.

Neurology, 7(9), 528–534. https://doi.org/10.1038/nrneurol.2011.107

[34] Cruise, K.E., Bucks, R.S., Loftus, A.M., Newton, R.U., Pegoraro, R., Thomas, M.G. (2010). Exercise and Parkinson’s: benefits for cognition and quality of life. Acta Neurologica Scandinavica, 123, 13-19. [35] Crizzle, A. M., & Newhouse, I. J. (2006). Is physical exercise beneficial for Persons with Parkinson’s disease. Clinical Journal of Sport Medicine, 16, 422–425.

[36] Tolosa, E., Wenning, G., & Poewe, W. (2006). The diagnosis of Parkinson's disease. The Lancet.

Neurology, 5(1), 75–86. https://doi.org/10.1016/S1474-4422(05)70285-4

[37] Wieckowski MR, et al. Recovering Mitochondrial Function in Patients’ Fibroblasts. In: Mitochondrial Biology and Experimental Therapeutics (ed^(eds) (2018).

[38] Goetz CG. [Movement Disorder Society-Unified Parkinson’s Disease Rating Scale (MDS-UPDRS): a

new scale for the evaluation of Parkinson’s disease]. Rev Neurol (Paris) 2010;166:1-4.

28

###### 14--Anexos

- Principios básicos de la Ética aplicada a la Investigación…………………………………………. 30-39

- C.I. General-URL ……..…………………………………………………………………………… 40-41

- Hoja de Información del paciente………………………………………………………………….. 42-49

- C.I. Específico

(Muestras biológicas para la investigación biomédica, y muestras biológicas sobrantes)………….. 50

- Anexo: Reglamento General de Protección de Datos (RGPD)……………………………………. 51

29

***Principios básicos de la Ética aplicada a la Investigación***

**INDICE:**

##### Fundamentación y Justificación de la investigación.

**- “La triada de un abordaje Dual, Físico-Coordinativo y Funcional, con un enfoque terapéutico”.**

1. **Consideraciones Éticas de esta investigación.**
2. **Apartado descripción del grupo vulnerable.**
3. **Apartado Consentimiento Informado e Información del paciente.**
4. **Apartado Principios básicos de la Ética aplicada a la Investigación. Tradición Anglo-Americana. “Un recorrido por los principios éticos de la investigación”**

**(Principio de no Maleficencia-Principio de Beneficencia-Principio de Autonomía-Principio de Justicia)**

1. **Apartado de Reflexión Ética sobre la investigación que realizaré.**
2. **Fundamentación y Justificación de la investigación.**

Creemos que es de vital importancia para el desarrollo de los principios éticos de la investigación, el correcto entendimiento del qué, porqué, para qué y el cómo, desarrollado en el Plan de Recerca (PR). También en el PR hemos citado estudios previos respecto a la enfermedad de Parkinson (EP) y la importancia de analizar los fibroblastos, para valorar su función mitocondrial. A esos estudios previos proponemos e incluimos, la dimensión de la intervención de la actividad física (AF). Hasta el día de hoy aún no se ha llevado a cabo, este tipo de investigación con intervención por parte de las Ciencias de la Actividad Física.

Al día de hoy es numerosa la evidencia de los beneficios de la AF en la EP. Centrándonos en nuestra investigación, tenemos como objetivo el exponer la relación entre la AF y la fisiopatología de la EP; y cómo afecta a los mecanismos de la función mitocondrial y su remodelación.

Los beneficios generales del ejercicio, también se espera en los pacientes con EP que realizan AF [33].

Hemos citados dos estudios que mostraron la implicancia de un programa de ejercicio aeróbico en el deterioro cognitivo en pacientes con EP, y también constataron efectos positivos en la función ejecutiva [29, 34]. Dentro de las pérdidas de funciones y deterioro, numerosas habilidades motrices se ven comprometidas, entre estas encontramos la postura, el equilibrio, la marcha y las transferencias. La AF ayuda a mejorar este rendimiento funcional [35]. Esta es una de las razones por las que hemos planteado en uno de los programas de AF, el abordaje Dual, Físico-Coordinativo y Funcional. Aunque en ambos programas de AF tenemos como objetivo adentrarnos en el estudio de la función mitocondrial, para una mejor comprensión de los efectos beneficiosos, de la AF como abordaje terapéutico.

Por esta razón es imprescindible el realizar una extracción de células de la piel, para analizar los fibroblastos de los pacientes con EP. El análisis de los fibroblastos de piel nos ayudará a valorar la función mitocondrial de los

30

pacientes; y también como la AF influye en los déficits proteolíticos y bioenergéticos celulares, asociados a la enfermedad.

En las conclusiones de Bloomer [21] vislumbramos una llamada a investigar en esta área, ya que se sorprenden de que ningún estudio hasta la fecha ha investigado el papel del ejercicio estructurado, para mejorar el estado oxidativo en personas con EP. Bloomer se centró en estudiar a nivel sanguíneo los efectos del entrenamiento de resistencia sobre el estrés oxidativo en la EP.

Hasta el momento no se han encontrado estudios que relacionen la clínica de la EP, que utilicen como biomarcadores a las organelas celulares (por medio de los fibroblastos) analizando el impacto de distintos tipos de AF, y cómo influyen en los déficits de la bioenergética celular. Esta es la primera investigación que estudiará los efectos de la AF, en la disfunción mitocondrial, utilizando el modelo de estudio de los fibroblastos de piel en personas con EP.

El estudio del sistema nervioso humano, posee la complejidad y dificultad debido a la inaccesibilidad del tejido, sumado a las limitaciones de los modelos celulares y animales de reproducir su fisiología. En lo que respecta a las investigaciones de mecanismos moleculares relacionadas con el envejecimiento y con las EN, hace años que se encuentran utilizando animales modificados genéticamente, pero que no terminan de reproducir del todo las patologías, ni los fenotipos asociados a la vejez.

Citando parte de una de las conclusiones del estudio de Ambrosi [10] “la investigación de los mecanismos patogénicos en las células periféricas, como los fibroblastos derivados de pacientes con EP esporádica (EPs) y controles emparejados por edad/sexo, podría generar una comprensión más profunda de los déficits que afectan a las neuronas dopaminérgicas, y posiblemente, nuevas herramientas aplicables a la práctica clínica.” Es así que, en medio de esta conclusión, nuestra investigación pretende incorporar/aportar una nueva variable, que será analizada por medio de los fibroblastos; para obtener nuevas conclusiones/resultados. Y poder aportar evidencia a nivel celular y molecular de cómo influye los distintos programas de AF en pacientes con EP.

##### “La triada de un abordaje Dual, Físico-Coordinativo y Funcional, con un enfoque terapéutico”.

La triada de un abordaje dual, físico-coordinativo y funcional, con un enfoque terapéutico, en donde la AF (planteada en 2 programas distintos) ejerce de hilo-conductor (y motor principal) de la biología celular (por un lado) y sus valores fisiológicos; enlazando a su vez con la fisiopatología de la EP (por el otro extremo de la “triada”) focalizada en la disfunción mitocondrial y estrés oxidativo. Para evaluar y analizar la clínica propia del paciente en cada etapa de la investigación, en lo que respecta a sus síntomas motores y no motores, psicológicos y de calidad de vida. Y de esta manera poder correlacionar la influencia, eficacia y eficiencia de los distintos programas de AF (propuestos por la investigación) en relación a los fibroblastos de piel de pacientes con EP, que ejercerán como biomarcador encargados de valorar la función mitocondrial y el estrés oxidativo. Siendo los Fibroblastos una valiosa alternativa a lo que sería una muestra de tejido cerebral, que por obvias razones de riesgos-beneficios estaría totalmente contraindicado.

31

Es por esto que proponemos un camino mínimamente invasivo, con una técnica conocida y ya implantada en diversos estudios. Dichos estudios, ya han arrojado resultados preliminares (citado en el plan de recerca), y el camino de esos estudios ha sido primero con animales y luego con humanos; y también han aportado luz a otras EN como el Alzheimer.

Creemos que es fundamental, hacer referencia y dejar constancia, que esta investigación cuenta con el apoyo del Center for Neuroscience and Cell Biology at the University of Coimbra (CNC-UC), Portugal. Una de las investigadoras principales del CNC-UC (Dra. Susana P. Pereira) es autora de los estudios citados en el PR (con resultados preliminares), y es Co-directora de esta tesis. Sumado a la Co-dirección del Dr. Joel Montané de la FCS-Universidad Blanquerna, cuyo apoyo y asesoramiento especializado en el complejo “universo” mitocondrial, hacen que esta investigación pueda adentrarse en la fisiopatología de la EP. Para investigar cómo responde la disfunción mitocondrial propia de la EP, teniendo en cuenta la clínica por la que transcurre el paciente; y poder así relacionar con su valoración motora (MDS-UPDRS III) y demás valoraciones, antes y después de la intervención de AF especialmente diseñada. Y también con la colaboración del Grupo de Enfermedades Neurodegenerativas del Hospital Universitario-Institut de Recerca Vall d’Hebron (VHIR), Barcelona. Mediante la supervisión del Dr. Jorge Hernández Vara (Neurólogo), con la colaboración de la neuróloga Daniela Samaniego Toro, y las investigadoras Dra. Marta Martínez-Vicente y Dra. Ariadna Laguna Tuset.

##### Consideraciones Éticas:

Esta investigación se plantea teniendo en cuenta los principios éticos de la tradición Americana y la legislación nacional e internacional aplicable en esta materia, así como la Carta de los Derechos Fundamentales de la Unión Europea y el Convenio Europeo de Derechos Humanos. Nos hemos basado en la tradición Anglo-Americana de los Principios Éticos de la Investigación. En todo momento de la investigación, se mantendrá presente la intimidad y privacidad física de las personas, como así también la confidencialidad de los datos solicitados y establecidos propios de la investigación. La investigación que hemos planteado siguen las normas que hacen referencia al Reglamento General Europeo de Protección de Datos (RGPD), Reglamento (UE) 2016/679; y privacidad y confidencialidad de datos (LOPDGD) Ley Orgánica (3/2018 de 5 de diciembre) de Protección de Datos Personales y garantía de los derechos Digitales. Los procedimientos y medidas establecidas para cumplir la privacidad y confidencialidad, se ha explicado en el documento de Información del paciente.

##### Apartado descripción del grupo vulnerable:

Las personas mayores son un grupo mayoritario en Europa, en los próximos años se dispararán las estadísticas de este colectivo y por ende las enfermedades verán como sus prevalencias aumentan.

Como ya hemos dicho en el plan de recerca, las EN se encuentran dentro de los trastornos neurológicos, entre otras enfermedades (OMS) [2]. Las EN tienen una gran implicación en el sistema sanitario y en la sociedad. “Al propio proceso de la enfermedad, hay que sumar el impacto psíquico, la mengua en la calidad de vida, la

32

incapacidad laboral, la pérdida de habilidades sociales, el gravamen de los cuidadores y las situaciones de dependencia” [4]. En España casi 1 millón de personas padecen una EN, donde el 40% de los afectados dejan de trabajar por la enfermedad, y el 53% tienen dificultad económica a causa de la misma (esto sin tener en cuenta que a día de hoy habría que sumar los efectos del COVID-19) [5]. La EP es la segunda EN más prevalente en la actualidad, después del Alzhéimer, y pertenece a los llamados Trastornos del Movimiento [6].

El Grupo Vulnerable a investigar en este Plan de Recerca, son pacientes con EP. Este grupo vulnerable ronda la cifra de 1 millón en Europa, y en España se estiman unas 160.000 personas. Con la dificultad agregada que posee su diagnóstico, esta cifra puede variar hasta casi 300.000 personas [5]. Y no menos importante, existe un 30% de pacientes con EP de inicio temprano aún no diagnosticados, y que no se han añadido a las cifras de la prevalencia. Siendo la dificultad del diagnóstico [36], uno de los motivos claves por los cuales gran parte de las investigaciones de hoy en día, pretenden identificar un biomarcador para optimizar su diagnóstico. En el marco de nuestra investigación, utilizaremos a los fibroblastos de piel como biomarcador, para la valoración de la función mitocondrial, en pacientes con EP que realizan distintos programas de AF.

Actualmente en las sociedades de la mayoría de los países de Europa, muestran un factor común respecto a la tasa de natalidad y la esperanza de vida (menor la primera y mayor la segunda); dando como consecuencia un envejecimiento progresivo de la población. Es este factor el que dispara una curva exponencial, respecto a la evolución de las EN en el mundo, duplicándose de aquí a 20 años, y se triplicará de aquí a 30 años (2050).

##### Apartado Consentimiento Informado e Información del paciente.

Se han elaborado los siguientes Consentimientos informado:

-C.I. General-URL -Hoja de Información del paciente (HIP)

-Permisos Centros

-C.I. Específico (Muestras biológicas para la investigación biomédica, y muestras biológicas sobrantes)

-En el documento de “(HIP)” se explica cómo se anonimizarán los datos, para preservar la intimidad de la persona. También hemos dejado constancia (y en el documento de información del paciente) de como los participantes de esta investigación verán reconocido sus derechos.

-La información sobre las Biopsias de los Fibroblastos de piel se encuentra al final de la HIP.

##### Apartado Principios básicos de la Ética aplicada a la Investigación. Tradición Anglo-Americana: “Un recorrido por los principios éticos de la investigación”

**(Principio de no Maleficencia-Principio de Beneficencia-Principio de Autonomía-Principio de Justicia)** Este apartado lo abordaremos realizando un recorrido por los principios éticos de la investigación, en relación a los aspectos más relevantes de este estudio.

33

Uno de los aspectos claves respecto a los Principios ético de esta investigación, lo hemos abordado desde el inicio del *Diseño* y el planteamiento de los *Objetivos* de esta investigación; y en base a la *fundamentación* y el *estado actual de la cuestión*. Nos referimos al uso de los fibroblastos de piel, que trae asociado la extracción de biopsia de piel, lo cual es irremplazable mediante otras técnicas. En este tipo de biopsia de piel (descriptas en la HIP), las molestias y los riesgos son mínimos; y a esto sumamos la experiencia de la Dra. Marta Martínez-Vicente en otros estudios de investigación, en los cuales también se han obtenido biopsias de piel de pacientes. Wieckowski_2018 afirma que *“los fibroblastos humanos (por Ej., de una biopsia de piel) son una fuente valiosa y confiable de material biológico para el estudio de una amplia gama de enfermedades, y es un procedimiento mucho menos invasivo en comparación con las biopsias de músculo o hígado.”* [37].

34

En un análisis profundo *del estado de la cuestión*, y bajo la supervisión de los directores de Tesis especializados en el Área de la Biología Celular (disfunción mitocondrial, estrés oxidativo) y Biomedicina, y con el respaldo de una de las investigadoras principales del CNC (Susana P. Pereira); hemos analizado estudios que afirman, que *“los fibroblastos de piel humana se han convertido en un camino muy acertado para estudiar diferentes patologías y trastornos neurodegenerativos como el Parkinson y la enfermedad de Alzheimer”* [10] (Ambrosi et al. , 2014 ; Cameron et al. , 2004 ; Hu et al. , 2015 ), “*debido a su metabolismo, disponibilidad y robustez fisiológicamente relevantes; y a su localización ya que pueden aislarse de los pacientes mediante métodos menos invasivos”* [22] en comparación a lo que sería aislar un tejido nervioso. Hay estudios que solo obtienen la información post mortem, debido a la inaccesibilidad del tejido.

Y es en los fibroblastos donde encontramos las mismas disfunciones que suceden en un tejido neuronal. Deus et al., 2020 detectaron alteraciones metabólicas y mitocondriales que también existirían en un tipo de célula no neuronal [11]. Por tanto, este estudio de Deus et al.,2020, es clave para nuestra investigación, ya que nos permite adentrarnos en uno de los aspectos de la fisiopatología de la EP, para evaluar la disfunción mitocondrial y que efectos produce un programa de AF.

También recomienda Auburger que *“las células primarias pueden tener varias ventajas para evaluar la toxicidad inducida por mitocondrias, incluido el metabolismo relevante, la accesibilidad humana y la traducción clínica”.* Sabemos por el *Marco Teórico,* que los Fibroblastos son protagonistas tanto en el mantenimiento, como en la reparación de los tejidos. Lo que hace que los Fibroblastos sea un biomarcador adecuado para el estudio de las EN según Auburger, es la capacidad de “*reflejar el daño celular acumulativo y las mutaciones”* [22].

Habiendo abordado uno de los aspectos claves de esta investigación, continuamos con otros aspectos también relevantes, de todo aquello que se tendrá en cuenta, para evitar causar un mal en primer lugar, y poder buscar un bien, antes, durante y después de la intervención.

##### Antes de la intervención:

-La correcta información del paciente, consentimiento informado, y explicación personal e individualizada.

-El análisis individualizado de la clínica que presenta el paciente en ese momento, la cual se realizará mediante una anamnesis (historia clínica detallada) para valorar la trayectoria y evolución que ha tenido su enfermedad.

-Se realizarán unos Test iniciales, no solo por el objetivo que tiene la investigación en cuanto analizar la intervención, sino también para tener en cuenta una posible modificación y adaptación de los parámetros de la AF a desarrollar, tales como carga, volumen, intensidad, repeticiones, frecuencia, recuperación, entre otras variables.

##### Durante la intervención:

-Cada una de las actividades programadas se llevarán a cabo habiendo realizado su correspondiente entrada en calor (general y específica) en función de los objetivos de la sesión.

35

-También se llevará a cabo al final de cada sesión, la “vuelta a la calma”, con el fin de recuperar los valores fisiológicos.

-En cada sesión se dejará un tiempo “Pre-sesión” y “Post-sesión”. Los cuales estarán destinados a valorar como llega el paciente, como se ha sentido desde la pasada sesión, se resolverán dudas y también se recogerá el feedback, una vez finalizada la sesión. Lo cual será clave para una correcta retroalimentación de lo planificado. **Después de la intervención:**

-Se seguirán realizando los controles pertinentes de la clínica, se les motivará y orientará para que mantengan unos niveles de actividad física aconsejables.

-Habrá un acompañamiento terapéutico durante todas las fases de la intervención (antes-durante y después).

Aunque ya hemos ido exponiendo la fundamentación y su justificación en este documento, ahora lo hacemos respecto de una de las dimensiones “del mal” (mal físico) del Principio de Maleficencia.

Somos muy conscientes de que un aspecto clave para esta investigación es el análisis de los fibroblastos de piel. Y en el mismo sentido es también clave desde el punto de vista de los principios básicos de la Ética aplicada a la Investigación. La recolección de las muestras de fibroblastos de piel, aunque técnicamente esté bajo el nombre de biopsia, es una de las menos invasivas, aunque no por esta razón somos menos conscientes de lo que significa para el paciente. Cada una de las etapas de esta investigación han sido “pesadas” en la “balanza riesgo- beneficios”; para asegurarnos de que sean mayores los beneficios a conseguir.

En el apartado de *discusiones* de los estudios de C. Deus y S. P. Pereira (2020) [11], nos afirma que *“la medición de la respuesta mitocondrial individual, a los agentes químicos en los fibroblastos de la piel, puede generar ahorros significativos en el deterioro de la salud del paciente.”*

Es casi inviable que un paciente por su propia cuenta, pueda solicitar un estudio de esta naturaleza. Y por esto creemos que es un aporte (contraprestación) a los pacientes que participen en la investigación.

Los resultados hallados no solo servirán de cara a la investigación y a la sociedad, y al resto de personas con Parkinson en España, Europa, y en todo el mundo. Con dichos resultados podremos optimizar con cada participante (paciente) de la investigación, cuantificándole su plan de AF lo más personalizado posible. Por medio del análisis de la muestra extraída de Fibroblastos, podremos evidenciar el estado actual de su enfermedad a nivel celular. Y así, obtendrá un examen detallado de los parámetros relacionados con su salud, su función física, y su función cognitiva. Al final de la intervención le entregaremos dicho plan de entrenamiento personalizado, junto con un informe final de los resultados y pruebas realizadas (Médicas, biológicas y físicas).

Esta será una de las contraprestaciones más valiosas que podrán beneficiarse los participantes de esta investigación.

36

Somos conscientes de que el llevar a cabo la actividad física programada, más otros tipos de test, pruebas biológicas y demás; todas ellas llevan consigo un tiempo de dedicación por parte de los sujetos investigados. Pero también somos conscientes que en el otro extremo de la balanza se encuentran los beneficios de los efectos de la actividad física programada y todo lo que puede aportar esta investigación a cada sujeto en particular.

También tenemos en cuenta que con la intervención estamos separando a los pacientes de su entorno habitual (3 veces por semana) para asignarlo en un grupo, entendemos que esto puede llegar a generar algún tipo de estrés inicial. Pero también sabemos del potencial social de la actividad física, y el potencial psicológico (aportando para disminuir las dimensiones del “mal social y mal psicológico”) de poder compartir experiencias con otras personas, que también comparten diagnóstico y el vínculo social de la actividad física compartida.

Estaríamos reforzando uno de los principios fundamentales para mantener una salud mental, la sociabilización. Por fortuna la actividad física, posee la dimensión y capacidad de poder tener efectos holísticos e integradores, y que el beneficio sea en todas las áreas.

Uno de los objetivos presentes en la búsqueda de la muestra (N), es que todos los tipos de intervenciones se puedan llevar a cabo en el mismo sitio, para evitar desplazamientos a los sujetos investigados.

Así como el evitar causar un mal es un deber, el causar un bien también los es. Es así que varias de las razones que hemos citado con el fin de evitar causar un mal, también son citables en este apartado, porque en sí mismo forman parte de los beneficios que se pretenden conseguir.

En esta investigación no nos conformamos con desearlo y solo citar referencias y bibliografía, lo cual estaría bastante bien, pero el diseño que hemos creado se basa en la intervención. Lo cual entraña toda una complejidad, y todo un diseño en el que tenemos el deber y toda la responsabilidad de llevarlo hacia su máxima expresión.

Procedemos a citar los efectos beneficiosos de la AF, sobre el organismo de una persona con EP. El mismo Speelman et al. (2011) [33] lo sintetiza en un decálogo de razones por las cuales el ejercicio físico puede beneficiar a pacientes con EP, y éstas son: “prevenir complicaciones cardiovasculares, detener la osteoporosis, mejorar la función cognitiva, prevenir la depresión, mejorar el sueño, disminuir el estreñimiento, disminuir la fatiga, mejorar el rendimiento funcional, mejorar la eficacia de la medicación y optimizar el sistema dopaminérgico”.

Esta interacción en distintas áreas del conocimiento como son la biología celular, las ciencias de la actividad física y la enfermedad de Parkinson traerá consigo beneficios a la sociedad, los cuales hemos citados en el plan de recerca, y el mayor de los beneficios que aspiramos, es el poder mejorar la calidad de vida de las personas con EP e indirectamente la de sus familiares.

Unos de los criterios de inclusión será que la cognición esté preservada, no solo por el diseño y los objetivos propios de la investigación, también tiene repercusión en el correcto entendimiento y aceptación del

37

consentimiento informado; lo cual se traduce en una garantía del principio de Autonomía, reforzado por el binomio *información-comprensión.*

También se tendrá en cuenta en la evaluación inicial (dentro de la anamnesis) si el sujeto vive solo, acompañado, y/o con sus hijos, para valorar la total autonomía y confirmar la comprensión del C.I.

La forma en que se anonimizarán los datos, será mediante un Código de Identificación del Sujeto. Es un Identificador único que asigna el investigador a cada sujeto del ensayo para proteger su identidad, y que se utiliza en vez del nombre del sujeto.

También en el documento de “Información del Paciente” hemos dejado constancia de como los participantes de esta investigación verán reconocido sus derechos.

En lo personal, esta tesis me permite volcar los conocimientos adquiridos durante varios años, desde distintas especialidades (siempre sobre los pilares de la Salud y la Educación), actualizarlos, y relacionarlo con nuevos conocimientos y nuevas áreas de especialización. Y de esta forma poder canalizar el foco, en los pacientes con EP.

Este estudio será un gran aporte al grupo SAFE y a la Universidad Blanquerna-URL, la cual me ha brindado la posibilidad de un contrato Pre-doctoral de Personal Investigador en Formación.

Sin duda alguna, el conocimiento obtenido en este proyecto también será de gran aporte al Center for Neurosciencie and cell Biology-University of Coimbra (Portugal), como así también al grupo de enfermedades Neurodegenerativas del Hospital Universitario VHIR.

También es un bien para mi persona, una REALIZACIÓN…un “SERVIR PARA SERVIR”.

##### 7. Apartado de Reflexión Ética sobre la investigación que realizaré

Esta investigación cuenta con el apoyo del contrato pre-doctoral para personal investigador en formación- Blanquerna (PIF), dentro del grupo SAFE. El motor principal es la Actividad Física; y si bien, el nivel de concreción está dentro de las EN, concretamente la EP, se podría vislumbrar que este tipo de intervenciones podría sentar precedente para el resto de las EN, ya que todas ellas comparten la disfunción mitocondrial. Esta investigación está diseñada para los pacientes con EP, pero la esencia protocolar de los fibroblastos de piel (desde el diseño de grupos para controles por edad y sexo, hasta la extracción y posteriores fases) también es útil para otras EN. Por lo tanto, una parte de esta investigación trasciende las fronteras de la EP, expandiéndose hacia las EN, con lo cual sin ser este nuestro objetivo principal, no deja de ser un valor a tener en cuenta desde una dimensión científica, innovadora y social. Remarcamos la dimensión social, ya que las EN están en continuo crecimiento debido a la pirámide poblacional europea, la cual influye directamente en este tipo de enfermedades donde el factor principal de predisposición, es la edad.

Se han tenido en cuenta dentro de los criterios de inclusión, aspectos claves que se utilizan en el día a día, no solo para el diagnóstico (el cual es meramente clínico), sino también para valorar la clínica que presentan los pacientes según avanza la enfermedad. Estos pilares fundamentales del diagnóstico y valoración son los

38

estadios de H&Y [29] y escala MDS-UPDRS III [38]. Y dentro de este grupo vulnerable con EP, se ha tenido en cuenta, que los efectos beneficiosos de esta intervención puedan alcanzar al mayor número de afectados por esta enfermedad. Debido a esta eficacia y eficiencia que buscamos, fundamentamos el criterio de inclusión de H&Y del estadio I-III; ya que es en los primeros estadios cuando más efectos beneficiosos pueden aportar las intervenciones terapéuticas, y la actividad física. En estadios y edades más avanzadas, los progresos son menos probables, las medicaciones dejan de tener efecto, al igual que otras terapias; y las demencias están más avanzadas.

En cada uno de estos apartados está impregnada la esencia de la responsabilidad y del deber. Responsables para responder a un área de la investigación en la que se puede obtener beneficios para el grupo vulnerable, en este caso los enfermos de Parkinson. Y es un deber, en esta ocasión que las ciencias de la actividad física sea el motor principal para llevar adelante este camino. En donde las ciencias de la actividad física y del deporte, ya de por si es un crisol de ciencias convergentes, interrelacionadas, yuxtapuestas; y en esta ocasión deja su impronta desde los inicios del diseño de esta investigación, donde establece lazos, conexiones y unión con distintas áreas del conocimiento. Desde la biología celular y molecular, hasta la valoración motora (incluyendo la evaluación y evolución clínica) y efectos de la actividad física planteada, en distintas combinaciones de capacidades físicas y funcionales.

##### Doctorando: Juan Carlos Magaña Gallardo

**Agosto 2021**

39

Comité de Ética de la Investigación

**CONSENTIMIENTO INFORMADO GEºNERAL**

Yo,……………………………………………………….,mayor de edad, con DNI,………………………….. actuando en nombre e interés propio.

**DECLARO QUE:**

He recibido información sobre el proyecto “Valoración de la función mitocondrial, utilizando fibroblastos de piel como biomarcador, en pacientes con enfermedad de Parkinson: efectos de dos programas de Actividad Física sobre la función motora, la calidad de vida, el sueño, aspectos cognitivos y el humor.” Del que se me ha entregado la hoja informativa anexa a este consentimiento y para el que se solicita mi participación. He entendido su significado, me han sido aclaradas las dudas y me han sido expuestas las acciones que se derivan del mismo. Se me ha informado/a de todos los aspectos relacionados con la confidencialidad y protección de datos en cuanto a la gestión de datos personales que comporta el proyecto y las garantías tomadas en cumplimiento del Reglamento General de Protección de Datos y de la Ley Orgánica 3/2018, de 5 de diciembre, de protección de datos personales y garantía de los derechos digitales.

También he recibido, con antelación y de forma satisfactoria, la explicación del procedimiento de extracción de Fibroblastos (Biopsia cutánea), su finalidad, riesgos, beneficios y alternativas. Que me han respondido todas las dudas, que comprendo la información recibida y que mi decisión es voluntaria.

Mi colaboración en el proyecto es totalmente voluntaria y tengo derecho a retirarme del mismo en cualquier momento, revocando el presente consentimiento, sin que esta retirada pueda influir negativamente en mi persona en sentido alguno. En caso de retirada, tengo derecho a que mis datos identificadores sean suprimidos, exceptuando que se podrían conservar si se anonimizan de manera que no se pueden vincular a mi persona.

Así mismo, renuncio a cualquier beneficio económico, académico o de cualquier otra naturaleza que pudiera derivarse del proyecto o de sus resultados.

Por todo ello,

**DOY MI CONSENTIMIENTO A:**

1. Participar en el proyecto “Valoración de la función mitocondrial, utilizando fibroblastos de piel como biomarcador, en pacientes con enfermedad de Parkinson: efectos de dos programas de Actividad Física sobre la función motora, la calidad de vida, el sueño, aspectos cognitivos y el humor.”
2. Que el equipo de investigación SAFE (Salut, Activitat física i Esport) de la Universidad de Blanquerna, el VHIR (Vall d´Hebron Institut de Recerca) y el Centro de Neurociencia y Biología celular de la Universidad de Coimbra (CNC-UC), el Investigador Principal del Proyecto el Dr. Jorge Hernández Vara, el Dr. Joel Montané (Universidad de Blanquerna) y el doctorando Juan Carlos Magaña; puedan tratar mis datos personales y difundir la información que el proyecto genere. Se garantiza que se preservará en todo momento mi identidad e intimidad, con las garantías establecidas en el Reglamento General de Protección de Datos y en la ley Orgánica 3/2018, de 5 de diciembre, de protección de datos personales y garantía de los derechos digitales y normativa complementaria.
3. Que doy mi consentimiento para el procedimiento de extracción de Fibroblastos de piel (Biopsia cutánea) y que conozco que tengo el derecho a revocarlo cuando lo desee, con la única obligación de informar al equipo médico.

40

1. También que las Instituciones nombradas en el punto anterior y sus investigadores y colaboradores; conserven todos los registros efectuados sobre mi persona en soporte electrónico, con las garantías y los términos legalmente previstos, si estuviesen establecidos, y a falta de previsión legal, por el tiempo que fuese necesario para cumplir las funciones del proyecto para las que los datos fueron recogidos.

En Barcelona, el / /

[FIRMA PARTICIPANTE] [FIRMA DEL IP]

41

HIP-Comité de Ética de la Investigación

## HOJA DE INFORMACIÓN DEL PARTICIPANTE (HIP)

**Título del proyecto:**

### “Valoración de la función mitocondrial, utilizando fibroblastos de piel como biomarcador, en pacientes con enfermedad de Parkinson: efectos de dos programas de Actividad Física sobre la función motora, la calidad de vida, el sueño, aspectos cognitivos y el humor.”

El investigador principal (IP) Dr. Jorge Hernández Vara del Hospital Universitari Vall d´Hebrón (HUVH), será quien llevará a cabo el reclutamiento. Responsables del servicio/grupo de enfermedades neurodegenerativas, junto a los miembros de nuestro equipo de investigación **Salut, Activitat Física i Eport (SAFE)** en un proyecto coordinado por el Dr. Joel Montané, estamos llevando a cabo el estudio: **“Valoración de la función mitocondrial, utilizando fibroblastos de piel como biomarcador, en pacientes con enfermedad de Parkinson: efectos de dos programas de Actividad Física sobre la función motora, la calidad de vida, el sueño, aspectos cognitivos y el humor.”**

Nuestro equipo de investigación desea invitarlo a participar en nuestro estudio. Antes que decida hacerlo, es extremadamente importante que comprenda por qué se está llevando a cabo esta investigación. Tómese unos minutos para leer detenidamente la siguiente información y no dude en preguntarnos lo que desee. Esta hoja de información para el participante explica el propósito de este estudio con más detalle y lo que implica si desea participar.

**¿Cuál es la finalidad de la investigación?**

El objetivo del estudio es evaluar su función mitocondrial, y como los efectos del ejercicio físico puede ayudar en el estado actual de su enfermedad.

La Enfermedad de Parkinson, y el resto de enfermedades neurodegenerativas, se caracterizan por presentar déficits en la función mitocondrial. Este estudio nos permitirá valorar en qué medida sus déficits bioenergéticos celulares asociados a su enfermedad, pueden mejorar con ejercicio físico. Para este fin utilizaremos, a los fibroblastos de piel (son pequeñas células de la superficie de su piel) como biomarcadores, ya que previamente se ha identificado que en estas células se puede evidenciar los mismos cambios que se producen a nivel neuronal. Los resultados de este estudio se obtendrán mediante la comparación, de dos grupos de pacientes en dos programas distintos de actividad física, y a la vez con un tercer grupo (grupo control) que no realizará los programas de actividad física, ni rehabilitación estándar (actualmente en el HUVH solo se lleva a cabo el seguimiento médico).

Todos los pacientes (de los 3 grupos) llevarán a cabo todas las visitas, test y los distintos procedimientos propios de la investigación.

Un estudio similar, pero sin el posible efecto terapéutico de un programa de actividad física se realizó previamente en el 2019 por uno de nuestros investigadores (no dude en consultarnos para ampliarle información).

En este estudio pretendemos determinar la diferencia de un plan de entrenamiento, para comprender como el diseño del ejercicio físico-terapéutico puede mejorar los síntomas motores y no motores, en pacientes con enfermedad de Parkinson.

**“Conocerlo es el primer paso para combatirlo” Lema de la Federación Española de Parkinson**

42

HIP-Comité de Ética de la Investigación

Para ello pretendemos monitorear los diferentes parámetros que se enumeran a continuación, durante el tiempo en el que usted participe en el programa de actividad física (4 meses):

- Evaluación Funcional y test específicos de actividad física (3 evaluaciones)
- Valoración motora MDS-UPDRS III, función cognitiva, calidad de vida, sueño y Humor (al inicio y final, y a los 8 meses de la evaluación inicial)
- Parámetros de salud (frecuencia cardíaca, tensión arterial, medidas antropométricas, altura, peso, IMC)
- Valoración de biomarcadores de Fibroblastos de piel (2 extracciones).

Los participantes del grupo control continuarán con su práctica diaria habitual y serán entrevistados una vez por semana por los investigadores para comprobar que sus rutinas no se han alterado. El grupo control podrá recibir 4 meses de AF del programa que haya obtenido los mejores resultados, en cuanto a sintomatología, y calidad de vida; después de la última valoración (8 meses).

Su participación es totalmente voluntaria. Sin embargo, si decide hacerlo, se le pedirá que firme un formulario de consentimiento general, y otro específico respecto a la conservación de muestras en el Centro de Neurociencia y Biología celular de la Universidad de Coimbra (CNC-UC). Puede retirarse de la investigación en cualquier momento y no necesita proporcionar las causas por las cuales lo hace. También, en caso de retirada, tiene derecho a que sus datos identificadores sean suprimidos si así lo desea, exceptuando que se podrían conservar si se anonimizan de manera que no se puedan vincular a su persona.

**Si Usted decide participar en nuestra investigación …**

En primer lugar, nos aseguraremos que comprenda lo que implica la investigación y le pediremos que complete un cuestionario de salud con la colaboración de nuestro investigador (Juan Carlos), para verificar su estado actual y firme un formulario de consentimiento. Todos los participantes tendrán asignado un código que evitará la identificación directa del participante con las muestras, con las respuestas dadas en los cuestionarios, y con los Test realizados, garantizando totalmente la confidencialidad.

Luego pasaremos a las evaluaciones iniciales. La **evaluación inicial** se compone de tests funcionales y cuestionarios de salud. Estas medidas también se volverán a realizar luego de 16 semanas (4 meses) para el mejor control de su evolución. Cuatro meses después de haber terminado el estudio, continuaremos registrando sus parámetros (a los 8 meses de la evaluación inicial). El número de visitas presenciales de seguimiento serán 3: inicial, 4 meses y 8 meses).

Los resultados de sus evaluaciones se registrarán, para analizar cualquier cambio que se deba implementar, en la planificación de las actividades programadas, ejercicios, volumen e intensidad.

Las evaluaciones se realizarán en las instalaciones del VHIR, y las específicas de Actividad Física en la Universidad Blanquerna-FCS, y se mantendrá igual para el resto de las evaluaciones (final y a los 8 meses). Le enviaremos un informe a su mail de contacto.

##### FCS-Blanquerna VHIR

|  |  |
| --- | --- |
|  |  |
| Día 1.C: Cuestionarios- VHIR | |
|  |  |
| Día 1.D: Valoración motora MDS-UPDRS III- VHIR | |
|  |  |
| Día 1.E: Extracción Fibroblastos-VHIR | |

|  |  |
| --- | --- |
|  |  |
| Día 1.A: Evaluación funcional y Test de AF- FCS-Blanquerna | |
|  |  |
| Día 1.B: Evaluación de la salud-FCS-Blanquerna | |

**“Conocerlo es el primer paso para combatirlo” Lema de la Federación Española de Parkinson**

43

##### Evaluación inicial

HIP-Comité de Ética de la Investigación

| **Evaluación funcional Y Test AF** | **Valoración motora MDS-UPDRS III** | **Evaluación de la salud** | **Extracción Fibroblastos** | **Cuestionarios** |
| --- | --- | --- | --- | --- |
| - Prueba de caminata 6´ - 1´(veces que se levanta y se sienta, en una silla) - Tiempo en levantarse de una silla, caminar   3 m, girar, caminar hacia atrás y sentarse  - Fuerza de prensión | -Valoración motora MDS-UPDRS III  (subescala motora) | - Presión arterial, FC - Altura-peso-IMC - Medidas Antropométricas   -Exploración Neurológica (Fuerza, reflejos, sensibilidad)   - Tests ortopédicos y neurodinámicos de extremidad superiores e inferiores   (EESS-EEII). | -Extracción Fibroblastos cutáneos | - (MoCA)  -Inventario de Depresión de Beck-IDB  -PD-CRS, Calidad de Vida, Humor y Sueño (SCOPA-AUT, NMS, PDSS, PDQ39,  Test de BERG, TINETTI y un cuestionario diario de caídas) |

##### FASE ESPAÑA

**PROGRAMAS DE ACTIVIDAD FÍSICA+EXTRACCIÓN DE FIBROBLASTOS MACROCICLO DE LOS PROGRAMAS DE ACTIVIDAD FÍSICA**

| *Evaluación Inicial* | *Semana 1* | 2 | 3 | *4* | *5* | *6* | *7* | *8* | *9* | *10* | *11* | *12* | *13* | *14* | *15* | *Semana 16* | *Evaluación Final* |
| --- | --- | --- | --- | --- | --- | --- | --- | --- | --- | --- | --- | --- | --- | --- | --- | --- | --- |


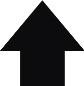


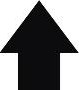
 Evaluación Inicial y Final: Extracciones de muestras+cuestionarios+evaluaciones

Esto se realizará a los 3 grupos (Todos los pacientes)

Control interno de la evolución de los Programas de Actividad Física.

Mesociclo 1: Periodo de adaptación individual a la carga, al volumen y a los ejercicios funcionales. Continuo Feedback Paciente-Investigador. Seguimiento semanal de la salud de los participantes.

Mesociclo 2: Primer aumento del binomio carga-volumen. Aumento de la ejecución motora en el programa (dual task training). Continuo Feedback Paciente-Investigador. Seguimiento semanal de la salud de los participantes.

|  |
| --- |
|  |
|  |
|  |

Mesociclo 3: Posibles modificaciones en base a los tests realizados en la semana 8 y 9. Continuo Feedback Paciente-Investigador. Seguimiento semanal de la salud de los participantes.

Mesociclo 4: último mesociclo de aumento en todas las dimensiones (teniendo en cuenta el principio de individualidad del entrenamiento) físicas, coordinativa y funcional Continuo Feedback Paciente-Investigador. Seguimiento semanal de la salud de los participantes.

**“Conocerlo es el primer paso para combatirlo” Lema de la Federación Española de Parkinson**

44

**¿Cuáles son las medidas que deseamos tomar?**

En primer lugar, le pediremos que complete algunos cuestionarios de uso común para evaluar la calidad de vida (utilizaremos el cuestionario específico para pacientes con Parkinson-PDQ39), el estado de ánimo (Escala de Humor de Brunel (BRUMS)), la función cognitiva (mental) utilizando la Montreal Cognitive Assessment (MoCA) y otras que hemos citado, y también valoraremos aspectos del sueño. Esto nos permitirá investigar si los ejercicios que realizaremos generan un efecto positivo, además de la función motora, también en la función cognitiva, el estado de ánimo, el sueño y en la calidad de Vida.

A continuación, tomaremos diferentes medidas de salud: presión arterial, frecuencia cardíaca en reposo, altura, peso y medidas antropométricas como la circunferencia de la cintura (preferiblemente debajo de la ropa).

Luego, también nos gustaría medir su **función física** pidiéndole que complete los siguientes tests funcionales: prueba de marcha de **6 minutos** (mediremos la distancia total recorrida en metros (m) utilizando una distancia previamente fijada de 10 m, 1 minuto de **Sit-to-Stands** (contaremos el número de veces que se levanta y se sienta, en una silla, durante 1 minuto), **Timed Up and Go** (calcularemos cuánto tiempo le lleva levantarse de una silla, caminar 3 m, girar, caminar hacia atrás y sentarse - usted hará 3 intentos de esta prueba) y **fuerza de agarre** (medida máxima de fuerza de la mano izquierda y derecha). Dentro de la función física, dejaremos constancia de su valoración motora específica de la enfermedad de Parkinson (MDS-UPDRS III) realizada por los neurólogos de nuestro equipo de investigación.

Nos gustaría seguir repitiendo las medidas de la valoración funcional (Test de AF), a las 16 y a las 32 semanas.

Finalmente, hemos de extraer una muestra muy pequeña de células de la piel, para investigar su función mitocondrial (la cual se encuentra afectada en todas las enfermedades neurodegenerativas), y así poder constatar el efecto (su función y/o estructura) de la aplicación de un programa de actividad física.

**¿Qué centros/Instituciones participan y qué haremos con los fibroblastos cutáneos? Participan las siguientes instituciones:**

- **Facultad de Ciencias de la Salud-Blanquerna-Universidad Ramón Llull.**
- **Facultad de Psicología y Ciencias de la Educación y del Deporte-Blanquerna-URL.**
- **Centro de Neurociencia y Biología Celular (CNC)-Universidad de Coimbra-Portugal.**
- **Vall d’Hebron Institut de Recerca (VHIR)**

Una vez que hayamos recolectado las muestras de los fibroblastos de piel se ampliarán y congelarán en el Vall d’Hebron Institut de Recerca. Una vez finalizado el estudio, todas las muestras congeladas, serán enviadas al laboratorio de Neurociencia (CNC) de la Universidad de Coimbra Portugal, para ser analizadas.

También le pedimos su consentimiento para que autorice almacenar una parte de las muestras en la sección de Colecciones (ref C.0006794) del Registro Nacional de Biobanco para que puedan ser utilizadas en otros proyectos de investigación biomédica, nacionales o internacionales. Cualquier estudio de investigación para el que se solicite la utilización de estas muestras deberá disponer de la aprobación del Comité de Ética de Investigación con medicamentos (CEIm) del Hospital Universitario Vall d’Hebron u otros comités que se establezcan según la legislación vigente. Este comité vela para que los investigadores desarrollen sus estudios siguiendo siempre las más estrictas normas éticas y legales. La cesión de muestras y datos asociados se realizará de manera que el investigador receptor no pueda conocer su identidad.

Los datos que se obtengan de su participación, no se utilizarán con ningún otro fin distinto del explicitado en esta investigación, siendo tratadas de manera confidencial bajo la responsabilidad del investigador principal. Dichos datos quedarán protegidos mediante clave de seguridad, y únicamente tendrán acceso a los mismos, los integrantes del equipo de investigación que se detallan en este documento. Si necesita más información puede contactar con la responsable de la colección la Dra. Marta Martínez-Vicente (marta.martinez@vhir.org).

**¿Cuánto tiempo llevará esto?**

Las evaluaciones funcionales y de salud tardan aproximadamente 25 minutos en completarse. Cognitivo, calidad de vida y los cuestionarios sobre el estado de ánimo tomarán aproximadamente 20-25 minutos. Y la función física otros 20-25 minutos. La extracción de los fibroblastos se hará en el mismo día.

Toda la evaluación inicial se completará durante 1 visita. Participar en todos los elementos del estudio en general puede demandar aproximadamente tres horas.

**“Conocerlo es el primer paso para combatirlo” Lema de la Federación Española de Parkinson**

45

HIP-Comité de Ética de la Investigación

**¿Qué debo hacer antes de las evaluaciones y qué debo usar? ¿Existe algún riesgo potencial?**

La extracción de las muestras de fibroblastos cutáneos, pueden causar una leve molestia durante algunas horas. Los riesgos son mínimos, además seguimos estrictos procedimientos de salud y seguridad (incluida la esterilización el área antes de tomar una muestra). Dicha extracción será realizada por uno de nuestros Neurólogos del equipo de investigación (Grupo de enfermedades neurodegenerativas) y personal experimentado del Vall d’Hebron Institut de Recerca (VHIR), utilizando procedimientos estándar de salud y seguridad. Dicha extracción será supervisada por uno de los IP del VHIR el Dr Jorge Hernández Vara, y también sumamos la experiencia de la Dra. Marta Martínez-Vicente que en otros estudios de investigación también ha obtenido biopsias de piel de pacientes.

**¿Qué pasará con los resultados de este estudio de investigación?**

Los datos recopilados se utilizarán para evaluar la eficacia de la clase de ejercicio para mejorar los parámetros de salud y la capacidad funcional. Se le proporcionarán informes sobre sus resultados personales, en un informe final. Los datos serán utilizados para publicar artículos con fines científicos, para presentaciones, conferencias nacionales e internacionales o para la elaboración de proyectos de disertación y tesis doctoral. Sin embargo, los datos personales serán anónimos y en ningún caso será posible identificar a las personas en los resultados informados para fines académicos, de evaluación o de publicación.

**¿Cuáles son los beneficios de participar en este estudio?**

En primer lugar, la información recopilada ayudará a desarrollar futuros programas de ejercicios para pacientes con EP. En segundo lugar, su participación también ayudará a arrojar luz, sobre los mecanismos por los cuales el ejercicio podría desempeñar un papel clave en la desaceleración de la progresión de la enfermedad de Parkinson. Por medio del análisis de la muestra extraída de Fibroblastos, podremos evidenciar el estado actual de su enfermedad a nivel celular. Y así, obtendrá un examen detallado de los parámetros relacionados con su salud, su función física, y su función cognitiva. Al final de la intervención, le diseñaremos un plan de entrenamiento personalizado, en base a los resultados de las pruebas realizadas (Médicas, biológicas y físicas).

Si decide participar en el estudio, es posible que del análisis de sus muestras biológicas se obtenga información relevante para su salud o la de su familia. Le entregaremos todo, junto a un informe final sobre sus resultados.

Es posible que de su participación en este estudio no se obtenga un beneficio directo. Sin embargo, la identificación de posibles factores, relacionados con la valoración de la función mitocondrial en pacientes con enfermedad de Parkinson que realizan actividad física; podría beneficiar en un futuro a otros pacientes con esta patología, y contribuir a un mejor conocimiento y tratamiento de esta enfermedad. De acuerdo con la legislación vigente, tiene derecho a ser informado de los datos que se obtengan en el curso del estudio. En el caso de que usted lo solicite, se le podrá facilitar información acerca de los estudios de investigación en los que hayan utilizado las muestras.

**Implicaciones de la información obtenida con el estudio:**

Si quiere conocer los resultados de investigación relevantes para su salud que se obtengan, infórmese a través de su médico sobre las implicaciones que esta información puede tener para su persona y su familia. Esta información se le comunicará si lo desea; en el caso de que prefiera no ser informado, su decisión se respetará. No obstante, cuando esta información, según criterio del médico responsable, sea necesaria para evitar un grave perjuicio para su salud o la de sus familiares biológicos, se informará a un familiar próximo o a un representante.

**“Conocerlo es el primer paso para combatirlo” Lema de la Federación Española de Parkinson**

46

HIP- Comité de Ética de la Investigación

**¿Cómo se mantendrá la confidencialidad de la información recopilada?**

Cualquier información sobre usted permanecerá confidencial, anónima y resguardada por el equipo de investigación. Todos los participantes tendrán asignado un código que evitará la identificación directa del participante con las respuestas dadas en cada cuestionario, tests realizados y pruebas médicas-biológicas, garantizando totalmente la confidencialidad.

Los datos que se obtengan de su participación no se utilizarán con ningún otro fin distinto del explicitado en esta investigación, siendo tratados de manera confidencial bajo la responsabilidad del IP y de todo el Grupo de Investigación. El Investigador Principal (IP) del VHIR el Dr. Jorge Hernández Vara (HUVH), el Dr. Joel Montané (Universidad de Blanquerna) y el doctorando Juan Carlos Magaña, serán los responsables del fichero de datos personales y de realizar la codificación de datos; y el resto de los investigadores accederán solo a datos codificados. Se garantiza que se preservará en todo momento su identidad e intimidad, con las garantías establecidas en el Reglamento General de Protección de Datos. Dichos datos quedarían protegidos mediante clave de seguridad.

Toda la documentación que contenga información personal se almacenará de acuerdo con la Ley de Protección de datos. En cualquier momento se podrá dirigir al IP (Investigador Principal), a cualquiera de los miembros del equipo de investigación que se especifica más adelante en este documento, o a la institución a la cual pertenecen, para ejercer sus derechos que reconoce la Ley Orgánica 3/2018, de 5 de diciembre, de Protección de Datos Personales y garantía de los derechos digitales.

La información recopilada solo se utilizará en relación con este proyecto y no se divulgará a terceros, y únicamente tendrán acceso a los mismos el Personal Investigador (los cuales se identifican en la próxima tabla de este documento) En el anexo de protección de datos encontrará más información sobre su política de protección de datos y la forma de ejercer sus derechos.

Nos ponemos a su disposición para resolver cualquier duda. Puede contactar con nosotros a través de los mails de contacto que citamos más abajo.

**¿Quién ha aprobado este estudio?**

Este estudio ha sido revisado y aprobado por el Comité Académico del Programa de Doctorado (URL-Blanquerna) y el Comité de Ética en Investigación de la Facultad de Psicología y Ciencias de la Educación y del Deporte (FPCEE-Universidad de Blanquerna). Y también ha sido aprobado por un Comité de Ética de la Investigación con medicamentos.

**¿Y si tengo una pregunta?**

Si tiene alguna pregunta sobre cualquier aspecto de este proyecto de investigación, puede hablar con:

| Personal Investigador | Área | Correo electrónico |
| --- | --- | --- |
| IP-Dr. Jorge Hernández Vara | Hospital Universitario-VHIR | jorhernandez@vhebron.net |
| Dra. Samaniego Toro, Daniela | Hospital Universitario-VHIR |  |
| Dra. Laguna Tuset, Ariadna | Hospital Universitario-VHIR |  |
| Dra. Martínez-Vicente, Marta | Hospital Universitario-VHIR |  |
| Dra. Maria Giné | Coordinadora del Grupo SAFE (Salut, Activitat Fisica i Esport) | [mariagg@blanquerna.url.edu](mailto:mariagg@blanquerna.url.edu) |
| Dr. Joel Montané | Co-Director de esta Investigación- Fase España | [joelmm@blanquerna.url.edu](mailto:joelmm@blanquerna.url.edu) |
| Dra. Susana P. Pereira | Co-Directora de esta Investigación- Fase Portugal | [pereirasusan@gmail.com](mailto:pereirasusan@gmail.com) |
| Ldo. Juan Carlos Magaña | Personal Investigador en formación- Blanquerna | [juancarlosmg@blanquerna.url.edu](mailto:juancarlosmg@blanquerna.url.edu)  607815095 |

**“Conocerlo es el primer paso para combatirlo” Lema de la Federación Española de Parkinson**

47

HIP-Comité de Ética de la Investigación

**En el contexto de esta investigación le pedimos su colaboración**

Ya que usted cumple los siguientes criterios de inclusión.

**Criterios de inclusión:**

-Pacientes que presenten un diagnóstico médico de EP idiopática, que se encuentren dentro de uno de los estadíos de la escala modificada de Hoehn y Yahr del I al III (incluido).

-Pacientes que tengan un buen estado cognitivo (puntuación en el Montreal Cognitive Assessment (MoCA) ≥26 puntos).

-Pacientes que hayan firmado el Consentimiento Informado.

-Pacientes con capacidad de caminar independientemente durante seis minutos.

-Edad entre 50 y 70 años.

-Pacientes con medicación estable (que no hayan tenido cambios en la medicación durante el último mes).

**Su colaboración implica participar durante 16 semanas en los programa de Actividad Física, con 3 sesiones por semana (sesiones de 1 hora), en la Universidad Blanquerna-Facultad de Ciencias de la Salud; ubicada en la** **C/ de Padilla, 326, 08025 Barcelona.**

**La Universidad (FCS)** **posee una cobertura de la póliza de responsabilidad civil, para diferentes proyectos que se llevan a cabo en la institución.**

**Y el número de visitas presenciales de seguimiento en el HUVH (Grupo de enfermedades Neurodegenerativas) serán 3: inicial (1 semana antes de comenzar los programas de AF), a los 4 meses (en la semana siguiente de haber terminado los programas de AF) y a los 8 meses.**

**Nota aclaratoria sobre el proceso de aleatorización de los distintos grupos.**

Esta investigación pretende comparar unos grupos de pacientes, en relación a los efectos de la actividad física (AF) en la función mitocondrial.

En este estudio se pretenden comparar dos procedimientos (2 programas de actividad física) y un grupo control.

La asignación vendrá determinada por el azar. Su médico no intervendrá en este proceso. Usted tendrá una probabilidad del 4,1% de recibir cada uno de los procedimientos contemplados en este estudio. Para llevar a cabo esta investigación necesitamos un mínimo de 24 pacientes diagnosticados con EP. La aleatorización de los veinticuatro pacientes, estará basada en la asignación a tres grupos (8 pacientes en cada grupo): 2 grupos realizarán programas de actividad física distintos (programa 1 y programa 2), y un tercer grupo no realizará ningún programa de actividad, conformando así un grupo de control. El grupo control podrá recibir 4 meses de AF del programa que haya obtenido los mejores resultados, una vez finalizada la intervención a los otros 2 grupos.

**“Conocerlo es el primer paso para combatirlo” Lema de la Federación Española de Parkinson**

48

HIP-Comité de Ética de la Investigación

**Información sobre la extracción de los Fibroblastos de piel**

1. Descripción y objetivos de la biopsia cutánea

El objetivo de la técnica es el estudio de la función mitocondrial y estrés oxidativo en la Enfermedad de Parkinson (EP).

La extracción de los fibroblastos consiste en una biopsia de piel mínimamente invasiva:

- Para obtener una muestra muy pequeña de piel (aproximadamente 3 mm2) de la cara interna del brazo.

- Para la toma de biopsia de piel se le aplicará por vía tópica de un anestésico local.

- La toma de biopsias de piel puede ocasionar dolor puntual en el momento de la punción y ligeras molestias después del procedimiento.

Esta muestra de la piel, es para analizarla en el Laboratorio de Biología Celular.

Durante el procedimiento, también es posible que se hagan fotografías o se obtengan imágenes exclusivamente para fines del presente estudio; y se pixelará la cara para que los sujetos no sean identificables. Le garantizamos que su intimidad será estrictamente respetada y que las imágenes obtenidas de este procedimiento nunca irán acompañadas de datos o informaciones que puedan revelar su identidad a terceros.

2. Alternativas razonables a la biopsia cutánea

En su caso particular, se ha considerado que éste es el medio más adecuado para valorar su función mitocondrial y sus déficits bioenergéticos celulares asociados antes y después de la realización de un programa de ejercicio físico. Y que, una vez establecido este, se le indicarán las opciones terapéuticas.

En este caso no existen otras alternativas para evaluar la remodelación metabólica del estrés oxidativo, el control de calidad mitocondrial, y de proteínas (y otros parámetros) en fibroblastos cutáneos de pacientes con EP.

3. Riesgos generales y/o específicos de la biopsia cutánea

A pesar de la adecuada elección de la técnica y de su correcta realización pueden presentarse efectos poco frecuentes, en ocasiones pueden aparecer otras molestias como sangrado, o infección de la herida, y reacción alérgica a los anestésicos empleados.

Se harán todas las pruebas y tratamientos necesarios para que los riesgos de la intervención se reduzcan al mínimo.

4. Riesgos personalizados de la biopsia cutánea

Otros riesgos o complicaciones que pueden aparecer teniendo son los relacionados con sus circunstancias personales (estado previo de salud, edad, profesión, creencias, medicaciones, etc.}.

En su caso concreto, los riesgos son: ……………………………………………………………………………………………

………………………………………………………………………………………………………………………………………..

5. Conservación de muestras en el Centro de Neurociencia y Biología celular de la Universidad de Coimbra (CNC-UC)

Las muestras biológicas obtenidas en el presente estudio se conservarán durante 6 años para la realización de estudios futuros. En el laboratorio de la Universidad de Coimbra, las muestras se usarán para profundizar en los efectos del secretoma del ejercicio físico en la función mitocondrial de pacientes con EP. Además, en función de los resultados obtenidos en esta investigación, las muestras biológicas sobrantes, también podrían utilizarse como base para conseguir financiación en otros proyectos de I+D.

En Portugal, la identificación de muestras e información de datos se realizará de acuerdo con la normativa general de protección de datos, y de la misma manera que en España, se utilizarán en investigaciones que hayan recibido el informe favorable de un Comité de Ética para la Investigación; de forma que no contradiga las preferencias expresadas por usted en el consentimiento firmado y en virtud de lo establecido en los art. 6, 7, 13 y/o 14 del Reglamento (UE) 2016/279 del Parlamento Europeo y del Consejo de 27 de abril de 2016 (RGPD) relativo a la protección de las personas físicas en lo que respecta al tratamiento de datos personales y a la libre circulación de estos datos.

En Barcelona, el / / .

[FIRMA PARTICIPANTE] [FIRMA DEL IP]

***“EN INVESTIGACIÓN, Y EN LA LUCHA CONTRA EL PARKINSON...***

***…CADA PASO CUENTA”***

***Juan Carlos Magaña Gallardo***

***Personal Investigador en Formación-Blanquerna***

**“Conocerlo es el primer paso para combatirlo”**

**Lema de la Federación Española de Parkinson**

49

| ***Consentimiento informado específico***  *(Muestras biológicas para la investigación biomédica, y muestras biológicas sobrantes)* | **Etiqueta** |
| --- | --- |
| **Núm. SAP:** |  |
| **Unidad / Servicio:** |  |

50

| ***Utilización de muestras biológicas y datos clínicos obtenidos durante el proceso asistencial para investigación biomédica y conservación de muestras biológicas sobrantes en el Biobanco del HUVH y en el Centro de Neurociencia y Biología celular de la Universidad de Coimbra Portugal (CNC-UC)*** |
| --- |
| *Después de haber recibido la hoja de información del paciente y comprendido su contenido, firmo este documento y autorizo al Biobanco del Hospital Universitario Vall d’Hebron (HUVH) y al Centro de Neurociencia y Biología celular de la Universidad de Coimbra Portugal(CNC-UC):*   - *A que las muestras biológicas sobrantes de las pruebas que me han realizado o me van a realizar* - *y la información clínica y asistencial associada*   *Se utilicen con la finalidad de llevar a cabo proyectos de investigación biomédica, nacionales o Internacionales, siempre que éstos cuenten con la obligada aprobación del Comité de Ética de Investigación competente. Cualquier estudio de investigación para el que se solicite la utilización de estas muestras deberá disponer de la aprobación del Comité de Ética de Investigación con medicamentos (CEIm) del Hospital Universitario Vall d’Hebron u otros comités que se establezcan según la legislación vigente.*  *1. Autorizo que las muestras biológicas sobrantes de los fibroblastos de piel y la información clínica asociada se utilicen para investigación, en los términos recogidos en la hoja de información.*  🞏 SÍ 🞏 NO  *2. Deseo que se me comunique la información derivada de la investigación que realmente sea relevante y aplicable para mi salud o la de mi família.*  🞏 SÍ 🞏 NO  *3. Autorizo a ser contactado en el caso de necesitar más información o muestras biológicas adicionales.*  🞏 SÍ 🞏 NO |

| *PACIENTE* | PERSONA QUE INFORMA | |  |
| --- | --- | --- | --- |
| *Nombre* | *Nombre* | |  |
| *Apellidos* | *Apellidos* | |  |
| DNI  *Edad* | DNI | |  |
| *Firma*  *Fecha* | *Firma*  *Fecha* | |  |
| *REPRESENTANTE:* | | |  |
| *Nombre* | *Relación con el donante:* | |  |
| *Apellidos* |  |  |  |
| DNI | *Tipo de representante:* | |  |
| *Firma*  *Fecha* | 🞏 | (1) *Autorizado por el donante* | |
|  | 🞏 | (2) *Legalmente autorizado* | |
|  | 🞏 | (3) *Autorizado por la familia* | |
|  |  | |  |

Comité de Ética de la Investigación

***Anexo: Reglamento General de Protección de Datos (RGPD)***

*En virtud de lo establecido en los art. 6, 7, 13 y/o 14 del Reglamento (UE) 2016/279 del Parlamento Europeo del Consejo, de 27 de abril de 2016 (RGPD), el Vall d`Hebron Institut de recerca (VHIR), el Servicio de enfermedades neurodegenerativas del Hospital Universitari Vall d`Hebron (HUVH), la Facultat de Psicología, Ciències de l`Educació i de l`Esport de Blanquerna, actuando como co-encargados del tratamiento, informan que los datos obtenidos a través del presente formulario serán captados y tratados (previamente codificados) por el Centro de Neurociencia y Biología celular de la Universidad de Coimbra Portugal (CNC-UC), en la forma y modo que se reflejan en el presente formulario, así como, también informarle que la base del presente tratamiento es el consentimiento expreso de usted proporcionado en el presente documento. Los datos personales serán utilizados con las únicas finalidades descritas en el presente documento. Los mismos serán conservados de acuerdo las exigencias legales hasta la finalización de las finalidades que hayan podido motivar su captación, y/o prescripción de las acciones legales que se pudieran derivar. Los datos personales captados en el presente documento y la información facilitada podrán ser comunicados a los investigadores acreditados, así como por obligación legal a las Administraciones competentes. La falta de autorización para el tratamiento de datos y el consentimiento para la realización del procedimiento descrito comportaran la imposibilidad de realizar las tareas descritas.*

*Le informamos que tiene derecho a solicitar el acceso, rectificación, portabilidad y supresión de los datos aportados y la limitación y oposición a su tratamiento de datos.*

***¿Con quién contacto?***

***-Investigador principal/colaborador del estudio:*** *Nombre y apellido. Dr. Jorge Hernández Vara*

*Teléfono +34932746235, Correo electrónico:* [jorhernandez@vhebron.net](mailto:jorhernandez@vhebron.net)

***-Investigador Coordinador:*** *Joel Montané*

***-Datos de contacto del DPD del Centro:*** *En cumplimiento del Reglamento (UE) 2016/679, el VHIR ha designado un delegado de Protección de datos, siendo sus datos de contacto* [*dpd@ticsalutsocial.cat*](mailto:dpd@ticsalutsocial.cat)

***-Datos de contacto del DPD del Promotor:*** [*dpd@blanquerna.url.edu*](mailto:dpd@blanquerna.url.edu)

*Facultad de Psicología y Ciencias de la Educación y del Deporte-Blanquerna (Grupo de Recerca SAFE)* *El Responsable del tratamiento de los datos es la Fundación Blanquerna.*

***-Datos de contacto del promotor por correo postal:*** *Passeig de Sant Gervasi, 47 de Barcelona (CP08022)*

51

Barcelona, ………………………………………………..

Sirva la presente para solicitar **permiso a su Centro/Asociación/Fundación**

……………………………………………………………………………………. al doctorando Juan Carlos Magaña Gallardo, Personal Investigador en Formación adscripto al grupo de investigación SAFE (Salut, Activitat Física i Esport),

de la Facultat de Psicologia, Ciències de l’Educació i de l’Esport-Blanquerna. Universitat Ramon Llull, para poder realizar cuestionarios y búsqueda de información, con el fin de su investigación.

Les rogamos, colaboración de su centro, y poder así formar parte de esta investigación.

Para cualquier consulta al respecto no duden en ponerse en contacto con nosotros en el siguiente correo electrónico: [joelmm@blanquerna.url.edu](mailto:joelmm@blanquerna.url.edu)

Agradeciéndole de antemano su colaboración,

reciba un cordial saludo.

Fdo

Barcelona, ………………………………………………..

52

54
