# Supplementary material for: Investigating the impact of physical activity on mitochondrial function in Parkinson’s disease (PARKEX): Study protocol for A randomized controlled clinical trial
Source: PLoS One. 2023 Nov 22;18(11):e0293774. doi: 10.1371/journal.pone.0293774 (PMC10664890; doi:10.1371/journal.pone.0293774)
Supplement: Peer review history

# Editor report: Decision Letter 0

**DOI:** 10.1371/journal.pone.0293774.r001

**Authors:** Vanessa Carels

27 Oct 2023

Investigating the impact of physical activity on mitochondrial function in Parkinson's disease (PARKEX): Study protocol for a randomised controlled clinical trial

PONE-D-23-33784

Dear Dr. Montane,

We’re pleased to inform you that your manuscript has been judged scientifically suitable for publication and will be formally accepted for publication once it meets all outstanding technical requirements.

Within one week, you’ll receive an e-mail detailing the required amendments. When these have been addressed, you’ll receive a formal acceptance letter and your manuscript will be scheduled for publication.

An invoice for payment will follow shortly after the formal acceptance. To ensure an efficient process, please log into Editorial Manager at http://www.editorialmanager.com/pone/, click the 'Update My Information' link at the top of the page, and double check that your user information is up-to-date. If you have any billing related questions, please contact our Author Billing department directly at authorbilling@plos.org.

If your institution or institutions have a press office, please notify them about your upcoming paper to help maximize its impact. If they’ll be preparing press materials, please inform our press team as soon as possible -- no later than 48 hours after receiving the formal acceptance. Your manuscript will remain under strict press embargo until 2 pm Eastern Time on the date of publication. For more information, please contact onepress@plos.org.

Kind regards,

Vanessa Carels

Staff Editor

PLOS ONE

Journal requirements:

When submitting your revision, we need you to address these additional requirements.

1. Please ensure that your manuscript meets PLOS ONE's style requirements, including those for file naming. The PLOS ONE style templates can be found at 

https://journals.plos.org/plosone/s/file?id=wjVg/PLOSOne_formatting_sample_main_body.pdf and 

https://journals.plos.org/plosone/s/file?id=ba62/PLOSOne_formatting_sample_title_authors_affiliations.pdf

2. Thank you for stating the following financial disclosure: 

"This work was supported by research funds provided by the Ajuts a l’Activitat de Recerca del Personal Docent i Investigador de la Universitat Ramon Llull (2021-URL-Proj-004), the Funding program PGRiD 2019–2021 of the Faculty of Psychology, Education, and Sport Sciences (APR-FPCEE2122/04) and by the European Regional Development Fund (ERDF), through the Centro 2020 Regional Operational Programme under project CENTRO-01-0246-FEDER-000010 (Multidisciplinary Institute of Ageing in Coimbra) through the COMPETE 2020 - Operational Programme for Competitiveness and Internationalisation and Portuguese national funds via FCT - Fundação para a Ciência e a Tecnologia, under projects 2022.01232.PTDC, UIDB/04539/2020, UIDP/04539/2020 and LA/P/0058/2020. S.P.P. was supported by FCT Pos-Doctoral fellowship SFRH/BPD/116061/2016. The authors would also like to acknowledge the support of the Faculty of Psychology, Education, and Sport Sciences, Blanquerna in providing funding for Predoctoral Researchers in Training (PFU) for J.C.M."

Please state what role the funders took in the study.  If the funders had no role, please state: ""The funders had no role in study design, data collection and analysis, decision to publish, or preparation of the manuscript."" 

If this statement is not correct you must amend it as needed. 

Please respond by return e-mail so that we can amend your financial disclosure and competing interests on your behalf.

# Editor report: Acceptance letter

**DOI:** 10.1371/journal.pone.0293774.r002

**Authors:** Vanessa Carels

10 Nov 2023

PONE-D-23-33784 

Investigating the impact of physical activity on mitochondrial function in Parkinson's disease (PARKEX): Study protocol for a randomized controlled clinical trial 

Dear Dr. Montane:

I'm pleased to inform you that your manuscript has been deemed suitable for publication in PLOS ONE. Congratulations! Your manuscript is now with our production department. 

If your institution or institutions have a press office, please let them know about your upcoming paper now to help maximize its impact. If they'll be preparing press materials, please inform our press team within the next 48 hours. Your manuscript will remain under strict press embargo until 2 pm Eastern Time on the date of publication. For more information please contact onepress@plos.org.

If we can help with anything else, please email us at plosone@plos.org. 

Thank you for submitting your work to PLOS ONE and supporting open access. 

Kind regards, 

PLOS ONE Editorial Office Staff

on behalf of

Dr. Vanessa Carels 

Staff Editor

PLOS ONE
